# Supplementary material for: Investigation on Metabolites in Structural Diversity from the Deep-Sea Sediment-Derived Bacterium Agrococcus sp. SCSIO 52902 and Their Biosynthesis
Source: Mar Drugs. 2022 Jun 29;20(7):431. doi: 10.3390/md20070431 (PMC9323897; doi:10.3390/md20070431)

Supplementary data for

Investigation on Metabolites in Structural Diversity from The Deep-Sea Sediment-Derived Bacterium *Agrococcus* sp. SCSIO 52902 and Their Biosynthesis

Wenping Ding [1,3], Yanqun Li [1,3], Xinpeng Tian [1], Min Chen [1,3], Zhihui Xiao [1], Rouwen Chen [1], Hao Yin [1,2,*], Si Zhang [1,2,*]

[1] *CAS Key Laboratory of Tropical Marine Bio-resources and Ecology, South China Sea Institute of Oceanology, Chinese Academy of Sciences, Guangzhou 510301, China*

[2] *Southern Marine Science and Engineering Guangdong Laboratory (Guangzhou), Guangzhou 511458, China*

[3] *University of Chinese Academy of Sciences, Beijing 100049, China*

Content

Table S1 Compositions of eleven liquid media 5
Fig. S1 HPLC analysis profiles of eleven fermentation conditions at 202 nm 6
Fig. S2 HPLC analysis profiles of monitoring different time sample in fermenter at 202 nm 8
Fig. S3 Variation in pH and DO of the large-scale fermentation 9
Fig. S4 Sets of fragmentation ions obtained in ESI-MS/MS of compounds 2 and 3 10
Fig. S5 HPLC analysis profiles of Marfey's derivatives of 2 at 340 nm 11
Fig. S6 HPLC analysis profiles of hydrolysate of 2 at 254 nm 12
Fig. S7 HPLC analysis profiles of 3 at 280 nm 12
Table S2 The retention times of Marfey's derivatives of 8 and 10-13 13
Fig. S8 Circular genome map of SCSIO 52902 13
Table S3 The putative biosynthetic gene clusters from SCSIO 52902 using antiSMASH platform 14
Fig. S9 Partial biosynthetic gene clusters from SCSIO 52902 15
Table S4 The key genes information related to the shikimate and OBS pathway. 15
Table S5 Inhibition rate against three human tumor cell lines 16
Fig. S10 HRESIMS spectrum of compound 1 17
Fig. S11 ^{1}H NMR spectrum (CD$_3$OD, 700 MHz) of compound 1 18
Fig. S12 ^{13}C NMR spectrum (CD$_3$OD, 176 MHz) of compound 1 18
Fig. S13 HSQC spectrum of compound 1 19
Fig. S14 HMBC spectrum of compound 1 19
Fig. S15 ^{1}H–^{1}H COSY spectrum of compound 1 20
Fig. S16 NOESY spectrum of compound 1 20

Crystallographic data of compound 1 21
Fig. S17 UV spectrum of compound 1 22
Fig. S18 ECD spectrum of compound 1 23
Fig. S19 IR spectrum of compound 1 24
Fig. S20 HRESIMS spectrum of compound 2 25
Fig. S21 HRESI-MS/MS-positive spectrum of compound 2 26
Fig. S22 ^{1}H NMR spectrum (CD_3OD, 700 MHz) of compound 2 27
Fig. S23 ^{13}C NMR spectrum (CD_3OD, 176 MHz) of compound 2 27
Fig. S24 HSQC spectrum of compound 2 28
Fig. S25 HMBC spectrum of compound 2 28
Fig. S26 ^{1}H–^{1}H COSY spectrum of compound 2 29
Fig. S27 NOESY spectrum of compound 2 29
Fig. S28 UV spectrum of compound 2 30
Fig. S29 ECD spectrum of compound 2 31
Fig. S30 IR spectrum of compound 2 32
Fig. S31 HRESIMS spectrum of compound 3 33
Fig. S32 HRESI-MS/MS-positive spectrum of compound 3 34
Fig. S33 ^{1}H NMR spectrum (CD_3OD, 700 MHz) of compound 3 (1) 35
Fig. S34 ^{13}C NMR spectrum (CD_3OD, 176 MHz) of compound 3 (1) 35
Fig. S35 ^{1}H NMR spectrum (CD_3OD, 700 MHz) of compound 3 36
Fig. S36 ^{13}C NMR spectrum (CD_3OD, 176 MHz) of compound 3 36
Fig. S37 HSQC spectrum of compound 3 37
Fig. S38 HMBC spectrum of compound 3 37
Fig. S39 ^{1}H–^{1}H COSY spectrum of compound 3 38
Fig. S40 NOESY spectrum of compound 3 38
Fig. S41 UV spectrum of compound 3 39
Fig. S42 IR spectrum of compound 3 39
Fig. S43 HRESIMS spectrum of compound 4 40
Fig. S44 ^{1}H NMR spectrum (CD_3Cl, 700 MHz) of compound 4 41
Fig. S45 ^{13}C NMR spectrum (CD_3Cl, 176 MHz) of compound 4 41
Fig. S46 HSQC spectrum of compound 4 42
Fig. S47 HMBC spectrum of compound 4 42
Fig. S48 ^{1}H–^{1}H COSY spectrum of compound 4 43
Fig. S49 UV spectrum of compound 4 43
Fig. S50 IR spectrum of compound 4 44
Fig. S51 HRESIMS spectrum of compound 5 45
Fig. S52 ^{1}H NMR spectrum (CD_3OD, 700 MHz) of compound 5 46
Fig. S53 ^{13}C NMR spectrum (CD_3OD, 176 MHz) of compound 5 46
Fig. S54 HSQC spectrum of compound 5 47
Fig. S55 HMBC spectrum of compound 5 47
Fig. S56 ^{1}H–^{1}H COSY spectrum of compound 5 48
Fig. S57 ESI-MS spectrum of compound 6 49
Fig. S58 ^{1}H NMR spectrum (CD_3OD, 500 MHz) of compound 6 50
Fig. S59 ^{13}C NMR spectrum (CD_3OD, 126 MHz) of compound 6 50

Crystallographic data of compound 6 51
Fig. S60 ESI-MS spectrum of compound 7 51
Fig. S61 ^{1}H NMR spectrum (CD_3OD, 500 MHz) of compound 7 52
Fig. S62 ^{13}C NMR spectrum (CD_3OD, 126 MHz) of compound 7 52
Crystallographic data of compound 7 53
Fig. S63 ESI-MS spectrum of compound 8 53
Fig. S64 ^{1}H NMR spectrum (CD_3OD, 500 MHz) of compound 8 54
Fig. S65 ^{13}C NMR spectrum (CD_3OD, 126 MHz) of compound 8 54
Crystallographic data of compound 8 55
Fig. S66 ESI-MS spectrum of compound 9 55
Fig. S67 ^{1}H NMR spectrum (CD_3OD, 500 MHz) of compound 9 56
Fig. S68 ^{13}C NMR spectrum (CD_3OD, 126 MHz) of compound 9 56
Crystallographic data of compound 9 57
Fig. S69 ESI-MS spectrum of compound 10 57
Fig. S70 ^{1}H NMR spectrum (CD_3OD, 700 MHz) of compound 10 58
Fig. S71 ^{13}C NMR spectrum (CD_3OD, 176 MHz) of compound 10 58
Fig. S72 ESI-MS spectrum of compound 11 59
Fig. S73 ^{1}H NMR spectrum (CD_3OD, 700 MHz) of compound 11 60
Fig. S74 ^{13}C NMR spectrum (CD_3OD, 176 MHz) of compound 11 60
Fig. S75 ESI-MS spectrum of compound 12 61
Fig. S76 ^{1}H NMR spectrum (CD_3OD, 700 MHz) of compound 12 62
Fig. S77 ^{13}C NMR spectrum (CD_3OD, 176 MHz) of compound 12 62
Fig. S78 ESI-MS spectrum of compound 13 63
Fig. S79 ^{1}H NMR spectrum (CD_3OD, 700 MHz) of compound 13 64
Fig. S80 ^{13}C NMR spectrum (CD_3OD, 176 MHz) of compound 13 64
Fig. S81 ESI-MS spectrum of compound 14 65
Fig. S82 ^{1}H NMR spectrum (CD_3Cl, 700 MHz) of compound 14 66
Fig. S83 ^{13}C NMR spectrum (CD_3Cl, 176 MHz) of compound 14 66
Fig. S84 ESI-MS spectrum of compound 15 67
Fig. S85 ^{1}H NMR spectrum (CD_3Cl, 700 MHz) of compound 15 68
Fig. S86 ^{13}C NMR spectrum (CD_3Cl, 176 MHz) of compound 15 68
Fig. S87 ESI-MS spectrum of compound 16 69
Fig. S88 ^{1}H NMR spectrum (CD_3OD, 700 MHz) of compound 16 70
Fig. S89 ^{13}C NMR spectrum (CD_3OD, 176 MHz) of compound 16 70
Fig. S90 ESI-MS spectrum of compound 17 71
Fig. S91 ^{1}H NMR spectrum (CD_3OD, 700 MHz) of compound 17 72
Fig. S92 ^{13}C NMR spectrum (CD_3OD, 176 MHz) of compound 17 72
Fig. S93 ESI-MS spectrum of compound 18 73
Fig. S94 ^{1}H NMR spectrum (CD_3OD, 500 MHz) of compound 18 74
Fig. S95 ^{13}C NMR spectrum (CD_3OD, 126 MHz) of compound 18 74
Fig. S96 ESI-MS spectrum of compound 19 75
Fig. S97 ^{1}H NMR spectrum (CD_3OD, 500 MHz) of compound 19 76
Fig. S98 ^{13}C NMR spectrum (CD_3OD, 126 MHz) of compound 19 76
Fig. S99 ESI-MS spectrum of compound 20 77

Fig. S100 ^{1}H NMR spectrum (CD_3OD, 500 MHz) of compound 20 78
Fig. S101 ^{13}C NMR spectrum (CD_3OD, 126 MHz) of compound 20 78
Fig. S102 ESI-MS spectrum of compound 21 79
Fig. S103 ^{1}H NMR spectrum (CD_3OD, 500 MHz) of compound 21 80
Fig. S104 ^{13}C NMR spectrum (CD_3OD, 126 MHz) of compound 21 80
Fig. S105 ESI-MS spectrum of compound 22 81
Fig. S106 ^{1}H NMR spectrum (CD_3OD, 700 MHz) of compound 22 82
Fig. S107 ^{13}C NMR spectrum (CD_3OD, 176 MHz) of compound 22 82
Fig. S108 ESI-MS spectrum of compound 23 83
Fig. S109 ^{1}H NMR spectrum (CD_3OD, 500 MHz) of compound 23 84
Fig. S110 ^{13}C NMR spectrum (CD_3OD, 126 MHz) of compound 23 84
Fig. S111 Integrated cluster-node diagram of Molecular Networking 85

Table S1 Compositions of eleven liquid media

Media	Compositions
1 mA	Glucose (10 g/L), Yeast extract (5 g/L), Soluble starch (12 g/L), Bacterial peptone (5 g/L), NaCl (4 g/L), KH_2PO_4 (0.5 g/L), $MgSO_4$•$7H_2O$ (0.5 g/L), $CaCO_3$ (2 g/L), Sea salt (30 g/L).
2 mK	Yeast extract (2 g/L), Bacterial peptone (2 g/L), Glucose (2 g/L), Mannitol (3 g/L), Malt extract (5 g/L), Peptone from soybean (5 g/L), Soluble starch (5 g/L), Sea salt (30 g/L).
3 m2216E	2216E (37.4 g/L), Glucose (15 g/L), Sea salt (30 g/L).
4 mAm2ab	Soluble starch (5 g/L), Peptone from soybean (5 g/L), Glucose (20 g/L), Yeast extract (2 g/L), Bacterial peptone (2 g/L), KH_2PO_4 (1 g/L), $MgSO_4$•$7H_2O$ (0.5 g/L), $CaCO_3$ (2 g/L), Sea salt (30 g/L).
5 mBHI	BHI broth (38.5 g/L), Sea salt (30 g/L).
6 mISP2	Yeast extract (4 g/L), Malt extract (10 g/L), Glucose (4 g/L), Sea salt (30 g/L).
7 mISP4	Soluble starch (10 g/L), KH_2PO_4 (2 g/L), $MgSO_4$•$7H_2O$ (1 g/L), NaCl (1 g/L), $(NH_4)_2SO_4$ (2 g/L), $CaCO_3$ (3 g/L), TS (1 mL/L), Yeast extract (0.5 g/L), Bacterial peptone (1 g/L), Sea salt (30 g/L).
8 mRA	Soluble starch (20 g/L), Glucose (10 g/L), Malt extract (10 g/L), Maltose (10 g/L), Corn steep liquor (5 g/L), $CaCO_3$ (2 g/L), Sea salt (30 g/L).
9 mJNP1A	Soluble starch (20 g/L), Peptone of fish powder (2 g/L), Trehalose Dihydrate (2 g/L), Beef extract (3 g/L), Sea salt (30 g/L).
10 mM20	Soybean flour (15 g/L), Soluble starch (20 g/L), Yeast extract (5 g/L), Bacterial peptone (2 g/L), NaCl (4 g/L), $CaCO_3$ (2 g/L), Sea salt (30 g/L).
11 mMCQ1	Soluble starch (10 g/L), Glucose (10 g/L), Bacterial peptone (5 g/L), Yeast extract (3 g/L), Beef extract (2 g/L), KH_2PO_4 (0.5 g/L), $MgSO_4$•$7H_2O$ (0.5 g/L), KBr (1 g/L), $CaCO_3$ (2 g/L), Sea salt (30 g/L).

TS: Trace salts solution, $FeSO_4$•$7H_2O$ 0.1 g, $MnCl_2$•$4H_2O$ 0.1 g, $ZnSO_4$•$7H_2O$ 0.1 g, in H_2O 100 mL.

Fig. S1 HPLC analysis profiles of eleven fermentation conditions at 202 nm

Experimental group

1 mA

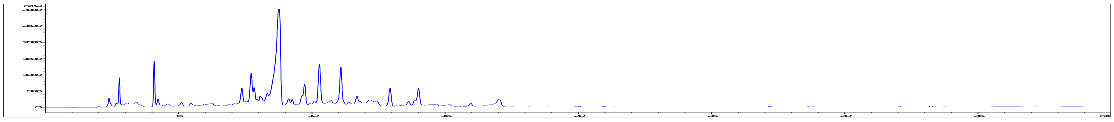

2 mK

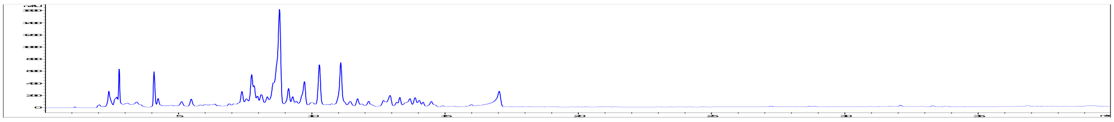

3 m2216E

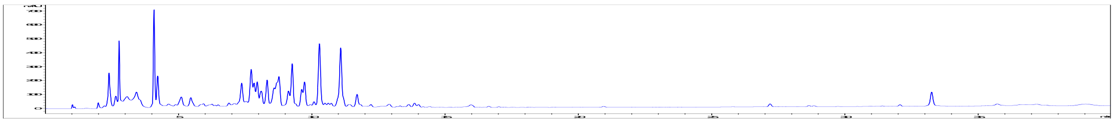

4 mAm2ab

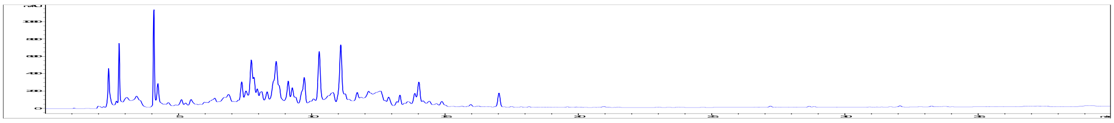

5 mBHI

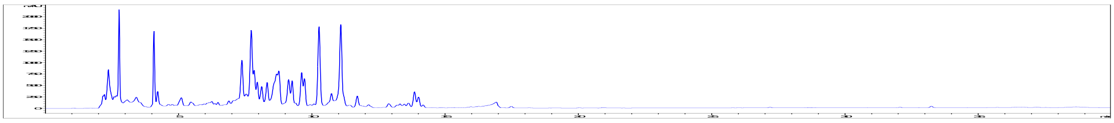

6 mISP2

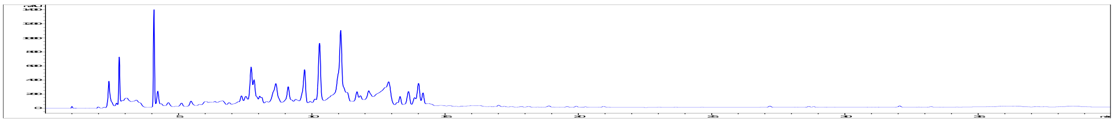

7 mISP4

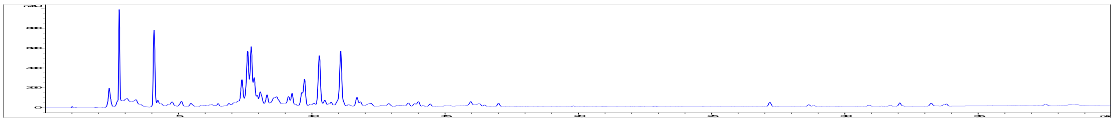

8 mRA

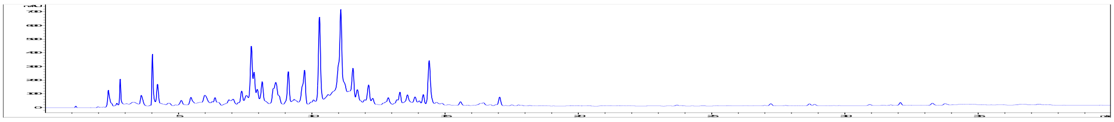

9 mJNP1A

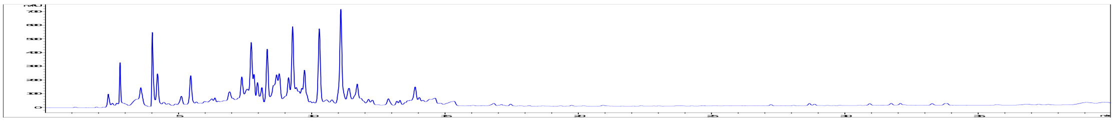

10 mM20

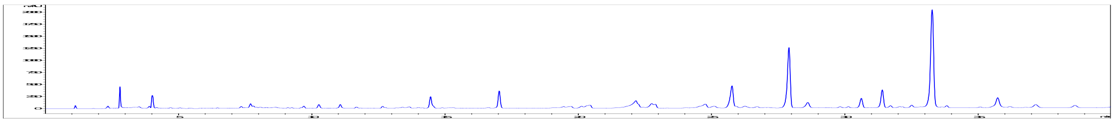

11 mMCQ1

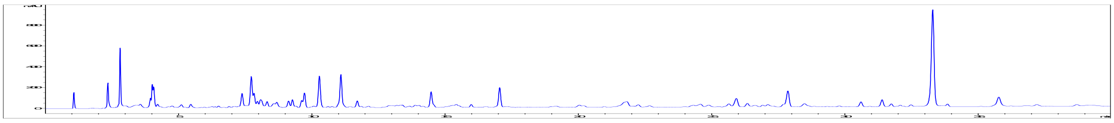

Blank group

1 mA

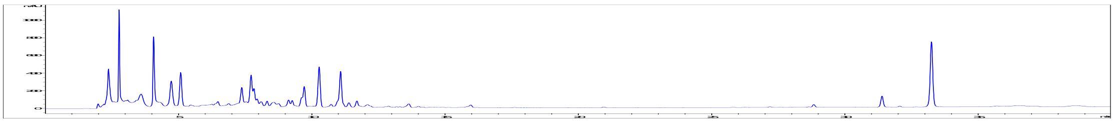

2 mK

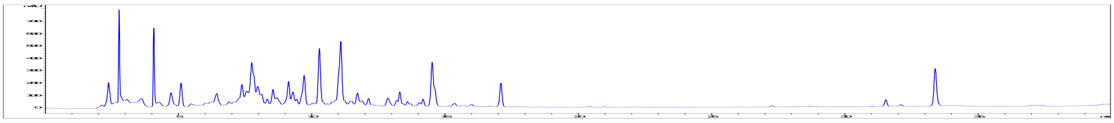

3 m2216E

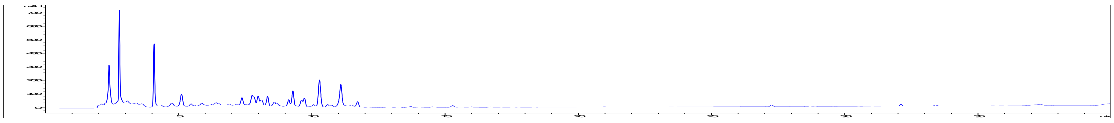

4 mAm2ab

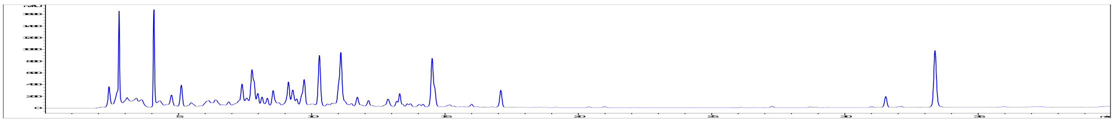

5 mBHI

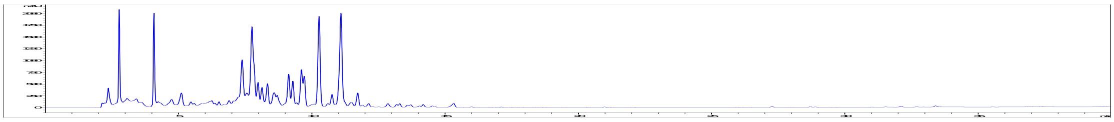

6 mISP2

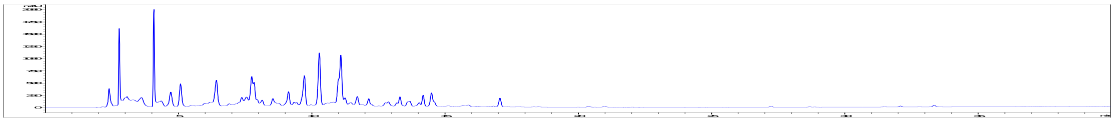

7 mISP4

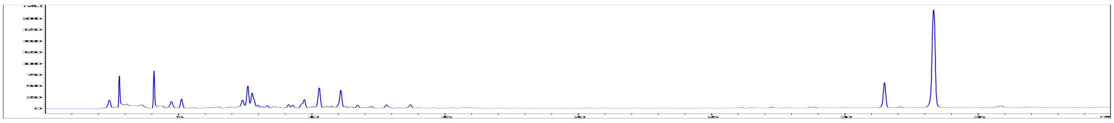

8 mRA

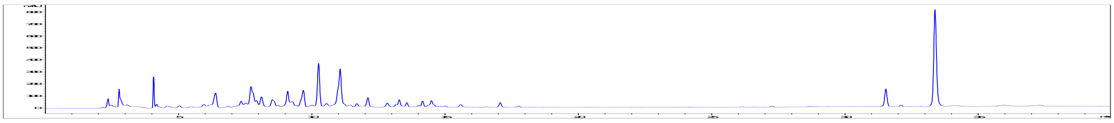

9 mJNP1A

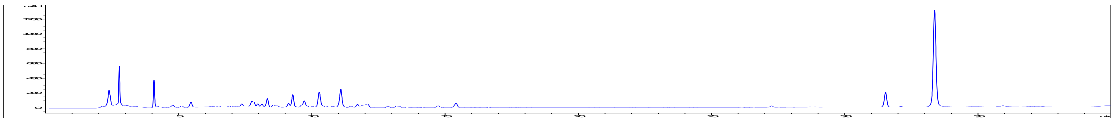

10 mM20

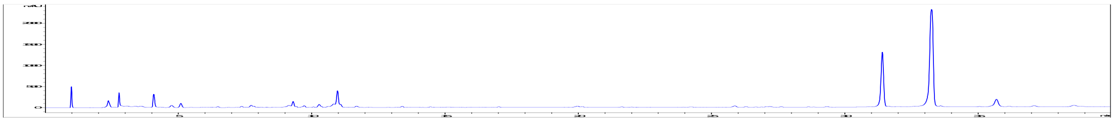

11 mMCQ1

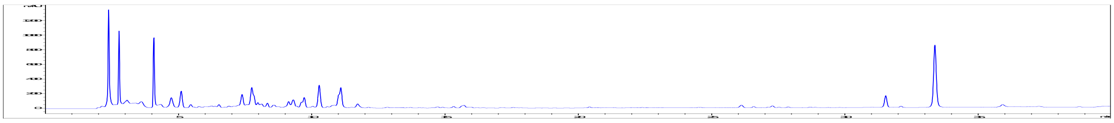

Fig. S2 HPLC analysis profiles of monitoring different time sample in fermenter at 202 nm

23 h

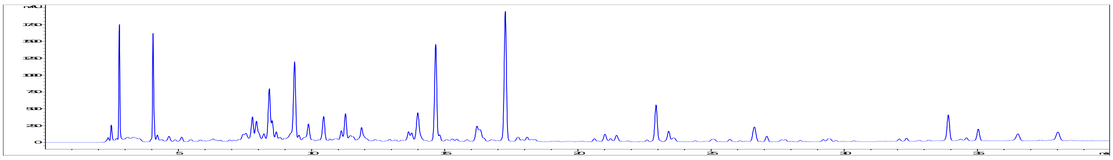

48 h

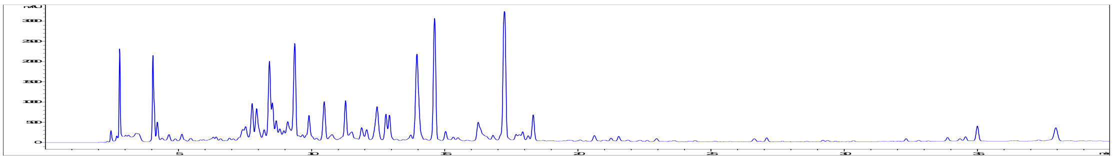

72 h

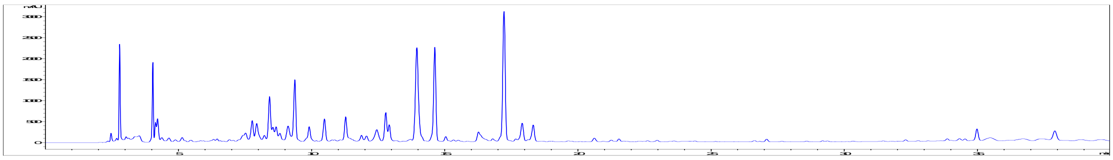

96 h

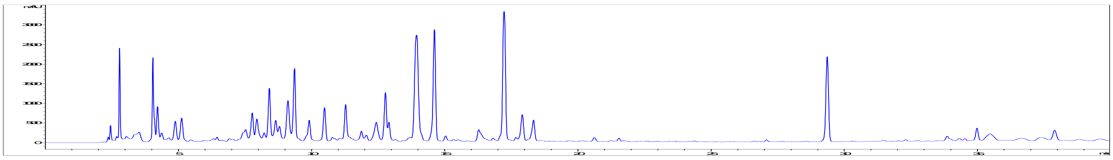

115 h

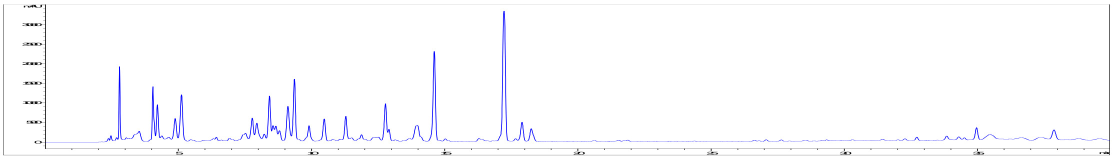

Fig. S3 Variation in pH and DO of the large-scale fermentation

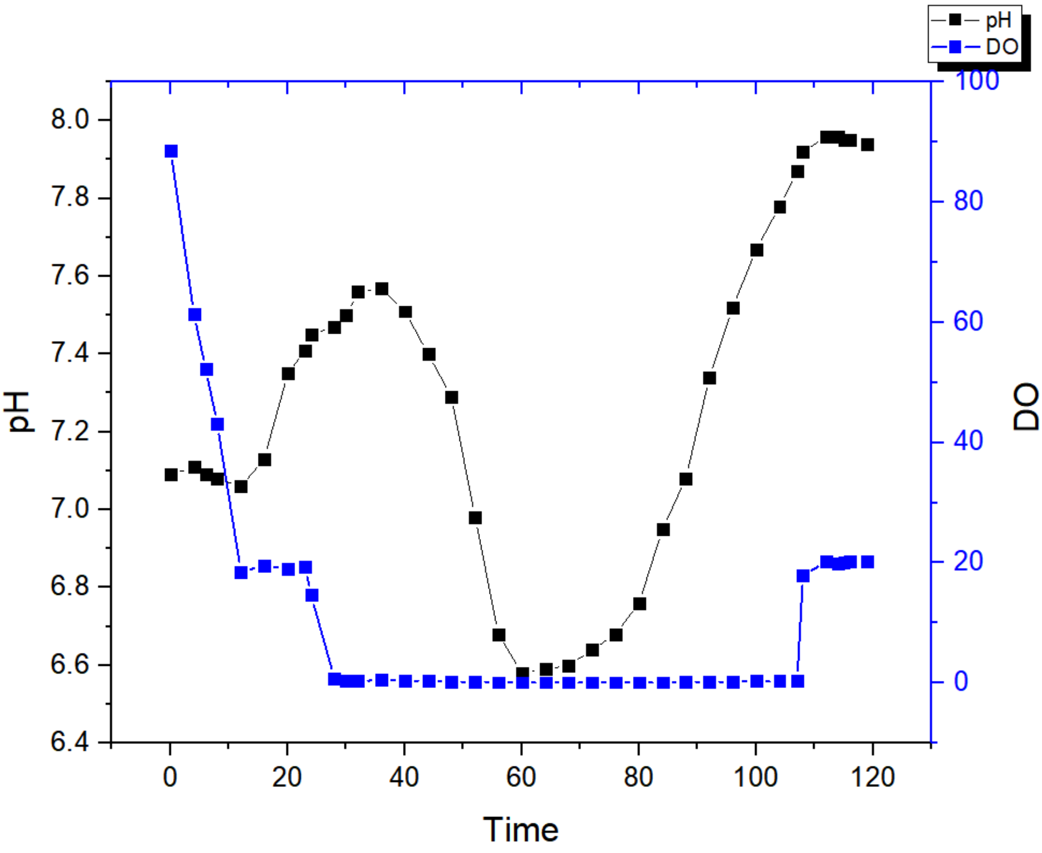

Fig. S4 Sets of fragmentation ions obtained in ESI-MS/MS of compounds **2** and **3**

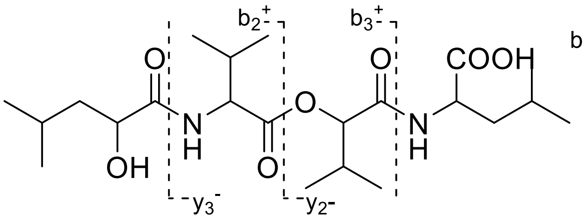

2

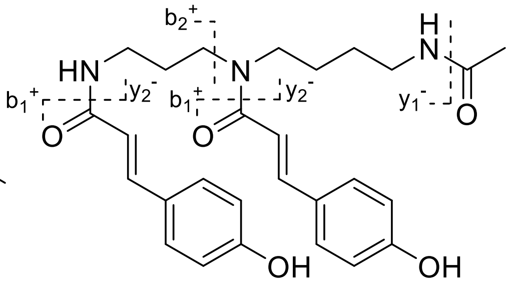

3

**2**		**3**	
fragmentation ions	*m*/*z*	fragmentation ions	*m*/*z*
precursor	445.2818	precursor	480.2358
b_2^+	214.14	b_1^+	147.04
$[b_2\text{-CO}]^+$	186.15	b_2^+	204.1
b_3^+	314.2	$[y_2\text{+2H}]^+$	334.21
$[y_2\text{+2H}]^+$	232.16	$[y_2\text{+2H-H}_2\text{O}]^+$	316.2
$[y_3\text{+2H}]^+$	331.22	$[y_2\text{+3H-b}_1]^+$	188.18
		$[y_1\text{+3H-b}_1\text{-NH}_3]^+$	275.18

Fig. S5 HPLC analysis profiles of Marfey's derivatives of **2** at 340 nm

Marfey's derivatives of **2**

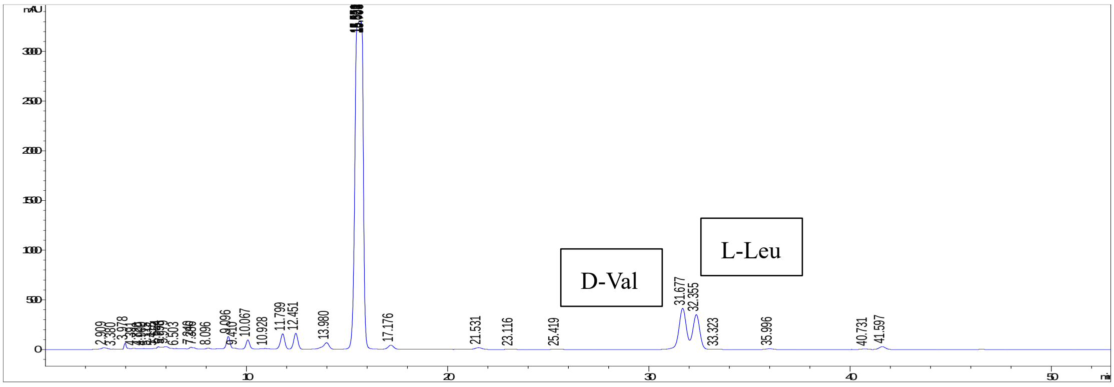

L-FDAA

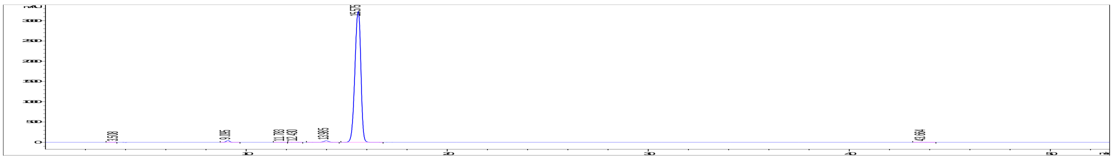

L-Val-FDAA

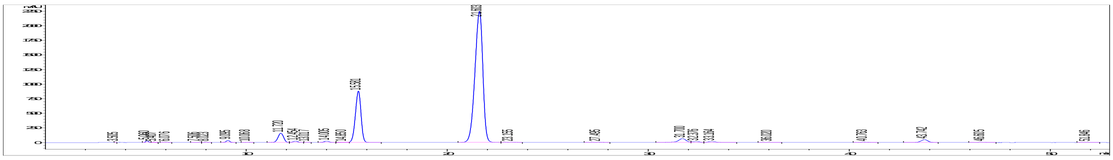

D-Val-FDAA

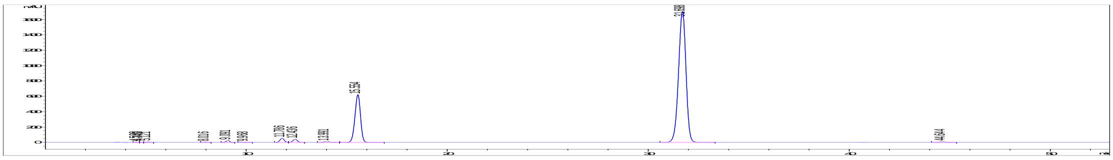

L- Leu -FDAA

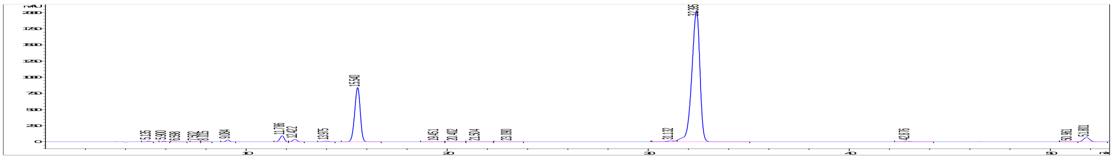

D-Leu-FDAA

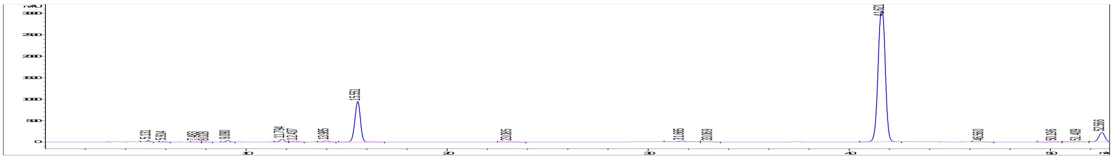

Fig. S6 HPLC analysis profiles of hydrolysate of **2** at 254 nm

Hydrolysate of **2**

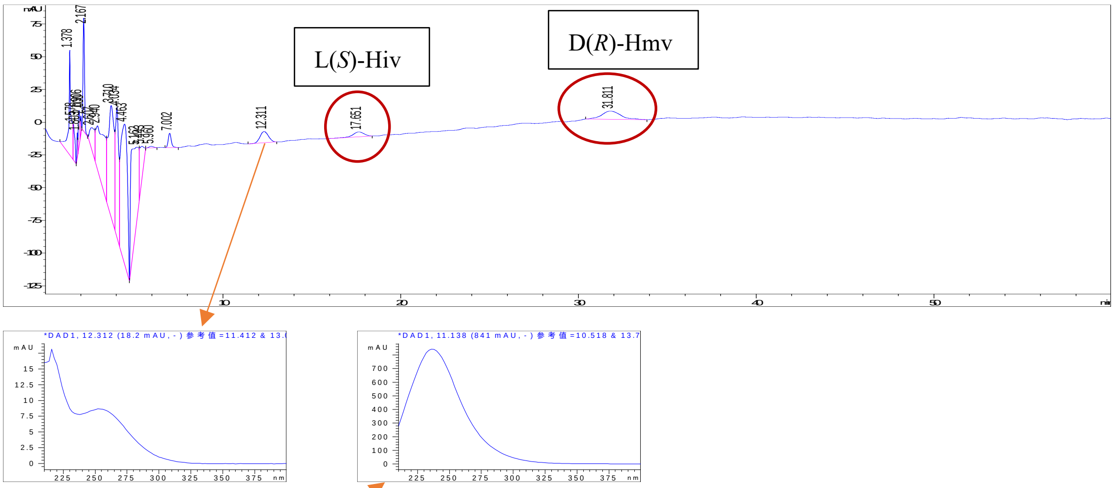

Four standard *α*-hydroxy acids

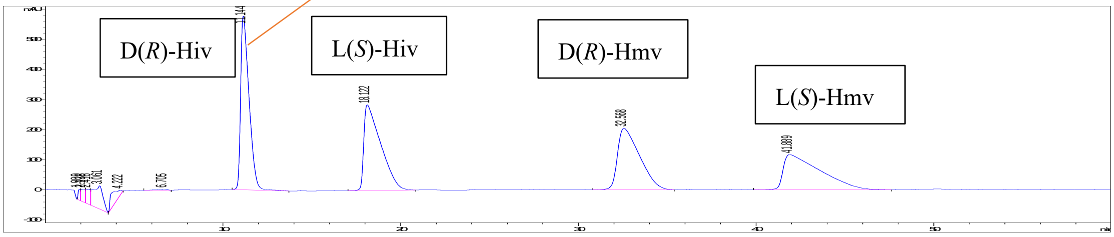

Fig. S7 HPLC analysis profiles of **3** at 280 nm

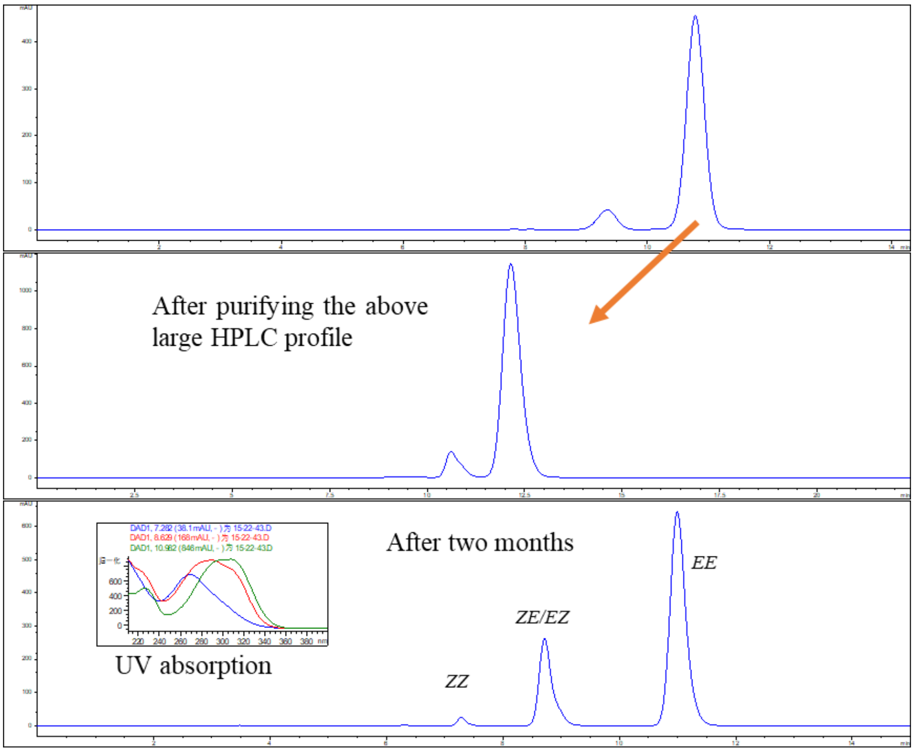

Using different analysis conditions

Table S2 The retention times of Marfey's derivatives of **8** and **10-13**

Sample	t_{RL}[a]					
	Pro	Ala	Val	Phe	Ile	Leu
standard	25.277	21.721	33.574	44.178	40.167	41.202
**8**	12.309[d]					41.156
**10**					40.049	
**11**	12.137[d]				40.052	
**12**		21.243			40.077	
**13**			33.036	43.545		

Sample	t_{RD}[b]						t_R[c]
	Pro	Ala	Val	Phe	Ile	Leu	L-FDAA
standard	27.718	37.388	40.945	49.916	47.595	47.926	25.648
**8**							25.532
**10**	27.18						25.286
**11**							25.311
**12**							25.332
**13**							25.386

[a] The retention times of L-amino acid derivatives. [b] The retention times of D- L-amino acid derivatives. [c] The retention time of L-FDAA. [d] The retention time of 3(*R*)-OH-Pro derivative.

Fig. S8 Circular genome map of SCSIO 52902

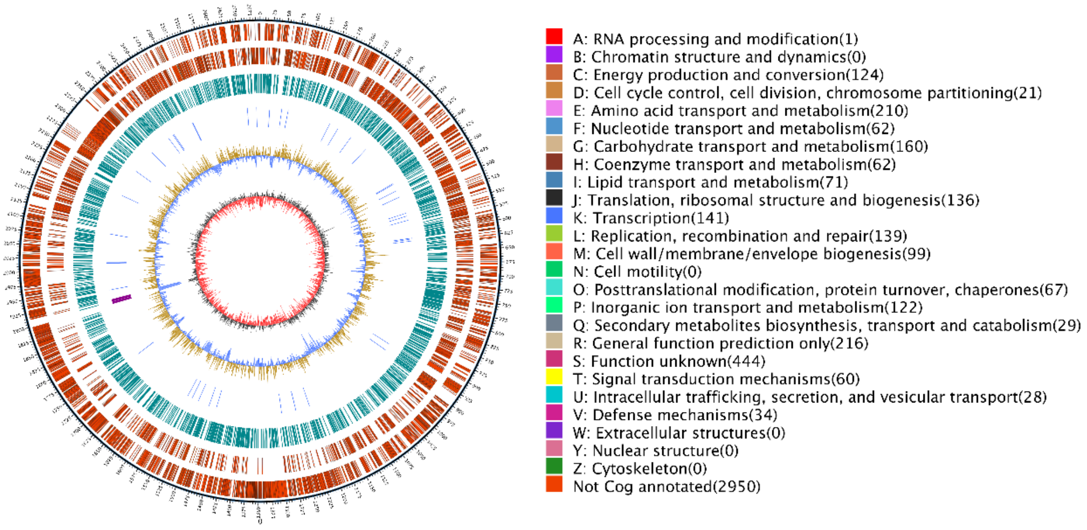

Table S3 The putative biosynthetic gene clusters from SCSIO 52902 using antiSMASH platform

Settings	Regions	Types	Location	Total
with the “relaxed strictness” setting	1.1	terpene	839,348 - 860,262 nt	20,915 nt
	1.2	T3PKS	1,327,415 - 1,368,557 nt	41,143 nt
	1.3	oligosaccharide	1,548,516 - 1,600,123 nt	51,608 nt
	1.4	betalactone	1,949,843 - 1,976,478 nt	26,636 nt
	1.5	redox-cofactor	2,027,514 - 2,055,420 nt	27,907 nt
with the “loose strictness” setting	2.1	saccharide	3 - 21,446 nt	21,444 nt
	2.2	saccharide, fatty_acid	247,226 - 289,189 nt	41,964 nt
	2.3	saccharide	314,706 - 377,916 nt	63,211 nt
	2.4	saccharide	398,747 - 420,622 nt	21,876 nt
	2.5	saccharide	485,313 - 527,212 nt	41,900 nt
	2.6	saccharide	528,766 - 565,722 nt	36,957 nt
	2.7	saccharide	669,928 - 691,728 nt	21,801 nt
	2.8	terpene	839,348 - 860,262 nt	20,915 nt
	2.9	saccharide	977,063 - 1,009,209 nt	32,147 nt
	2.10	saccharide	1,217,315 - 1,244,224 nt	26,910 nt
	2.11	T3PKS	1,327,415 - 1,368,557 nt	41,143 nt
	2.12	saccharide	1,437,602 - 1,498,602 nt	61,001 nt
	2.13	saccharide, oligosaccharide	1,544,536 - 1,600,123 nt	55,588 nt
	2.14	betalactone, saccharide	1,949,843 - 1,992,667 nt	42,825 nt
	2.15	redox-cofactor	2,027,514 - 2,055,420 nt	27,907 nt
	2.16	saccharide	2,168,764 - 2,231,223 nt	62,460 nt
	2.17	fatty_acid	2,413,831 - 2,436,419 nt	22,589 nt
	2.18	saccharide	2,537,533 - 2,567,647 nt	30,115 nt
	2.19	saccharide	2,599,638 - 2,652,795 nt	53,158 nt

Fig. S9 Partial biosynthetic gene clusters from SCSIO 52902

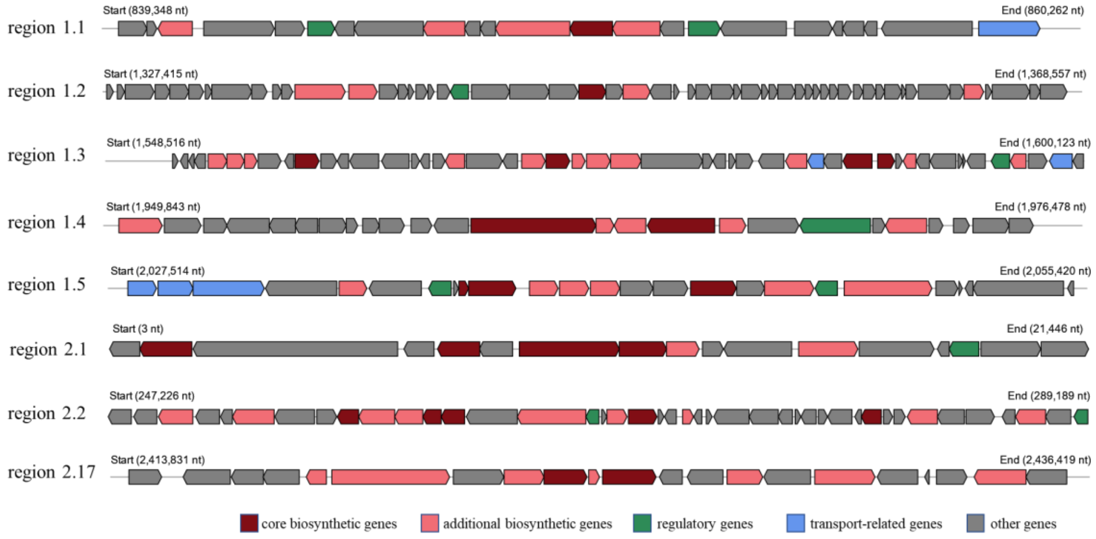

Table S4 The key genes information related to the shikimate and OBS pathway.

Gene	Orientation	Location	Swissprot_target_ID	E-value	Identity	Cover
aroH	(+)	1967385-1968767 (1383 nt)	P80574(AROF_STRCO)	3.00E-157	61.24%	94.57%
aroB	(-)	2329535-2328474 (1062 nt)	Q6AF95(AROB_LEIXX)	1.00E-128	65.72%	99.43%
aroK	(-)	2330095-2329556 (540 nt)	Q9KXQ5(AROK_STRCO)	8.30E-23	45.45%	83.24%
aroC	(-)	2331279-2330092 (1188 nt)	A5CRZ1(AROC_CLAM3)	2.20E-169	77.92%	99.49%
aroE	(-)	2332133-2331282 (852 nt)	Q39VU8(AROE_GEOMG)	8.30E-17	40.11%	61.84%
trpG	(-)	928266-927607 (660 nt)	P9WN34(TRPG_MYCTO)	2.20E-57	58.08%	88.58%
pchA	(+)	564511-565722 (1212 nt)	Q51508(PCHA_PSEAE)	5.60E-35	31.84%	89.58%
menD	(-)	570394-568727 (1668 nt)	Q6AHC2(MEND_LEIXX)	1.90E-118	48.48%	98.38%
menC	(+)	579653-580621 (969 nt)	A0QRG0(MENC_MYCS2)	7.60E-83	57.78%	97.52%
menE	(-)	574492-573491 (1002 nt)	P9WQ38(MENE_MYCTO)	7.20E-36	35.03%	90.09%
menB	(-)	575427-574528 (900 nt)	A0QRD3(MENB_MYCS2)	6.30E-124	74.33%	97.99%

Table S5 Inhibition rate against three human tumor cell lines

**Inhibition rate (30 *μ*M)**			
Compounds	A-549	HL-60	HCT-116
**1**	3.93%	-2.36%	2.00%
**2**	3.24%	-10.65%	1.42%
**3**	2.79%	-1.82%	5.74%
**4**	1.84%	2.63%	1.14%
**5**	24.01%	77.12%	5.34%
**6**	3.65%	4.42%	1.72%
**7**	3.25%	1.38%	0.97%
**8**	4.24%	3.54%	1.07%
**9**	7.17%	-1.55%	0.82%
**10**	8.02%	-1.38%	1.05%
**11**	-0.88%	-1.61%	2.41%
**12**	-2.04%	-2.89%	0.48%
**13**	-1.37%	-1.67%	1.60%
**14**	-1.22%	-3.48%	-0.09%
**15**	-1.98%	-6.37%	1.62%
**16**	-2.63%	5.86%	-0.40%
**17**	-1.59%	-7.34%	-3.47%

Fig. S10 HRESIMS spectrum of compound **1**

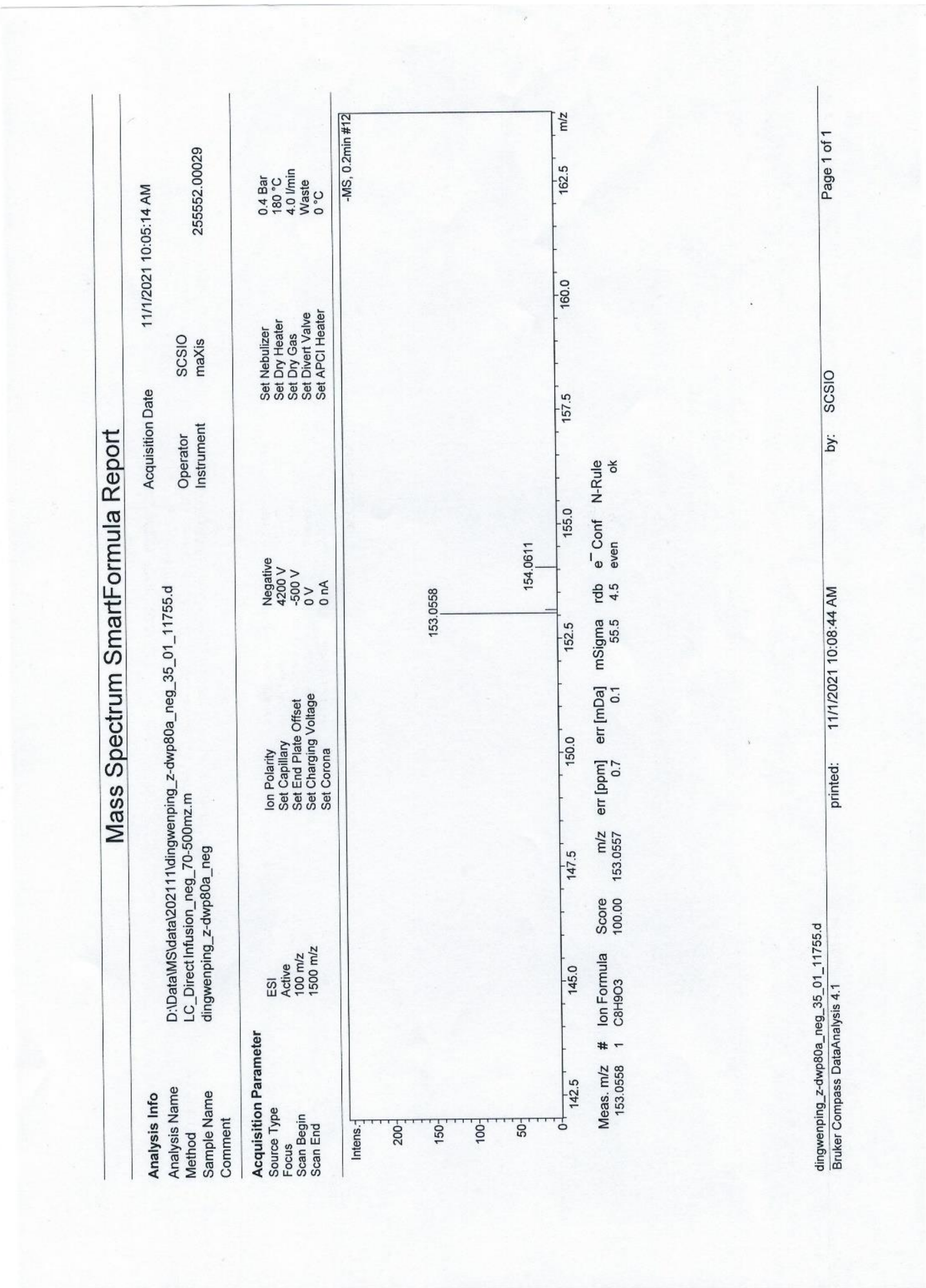
Mass Spectrum SmartFormula Report

**Analysis Info**		Acquisition Date	11/1/2021 10:05:14 AM
Analysis Name	D:\Data\MS\data\202111\dingwenping_z-dwp80a_neg_35_01_11755.d		
Method	LC_Direct Infusion_neg_70-500mz.m	Operator	SCSIO
Sample Name	dingwenping_z-dwp80a_neg	Instrument	maXis 255552.00029
Comment			

**Acquisition Parameter**					
Source Type	ESI	Ion Polarity	Negative	Set Nebulizer	0.4 Bar
Focus	Active	Set Capillary	4200 V	Set Dry Heater	180 °C
Scan Begin	100 m/z	Set End Plate Offset	-500 V	Set Dry Gas	4.0 l/min
Scan End	1500 m/z	Set Charging Voltage	0 V	Set Divert Valve	Waste
		Set Corona	0 nA	Set APCI Heater	0 °C

Meas. m/z	#	Ion Formula	Score	m/z	err [ppm]	err [mDa]	mSigma	rdb	e⁻ Conf	N-Rule
153.0558	1	C8H9O3	100.00	153.0557	0.7	0.1	55.5	4.5	even	ok

dingwenping_z-dwp80a_neg_35_01_11755.d
Bruker Compass DataAnalysis 4.1 printed: 11/1/2021 10:08:44 AM by: SCSIO Page 1 of 1

Fig. S11 ^{1}H NMR spectrum (CD_3OD, 700 MHz) of compound **1**

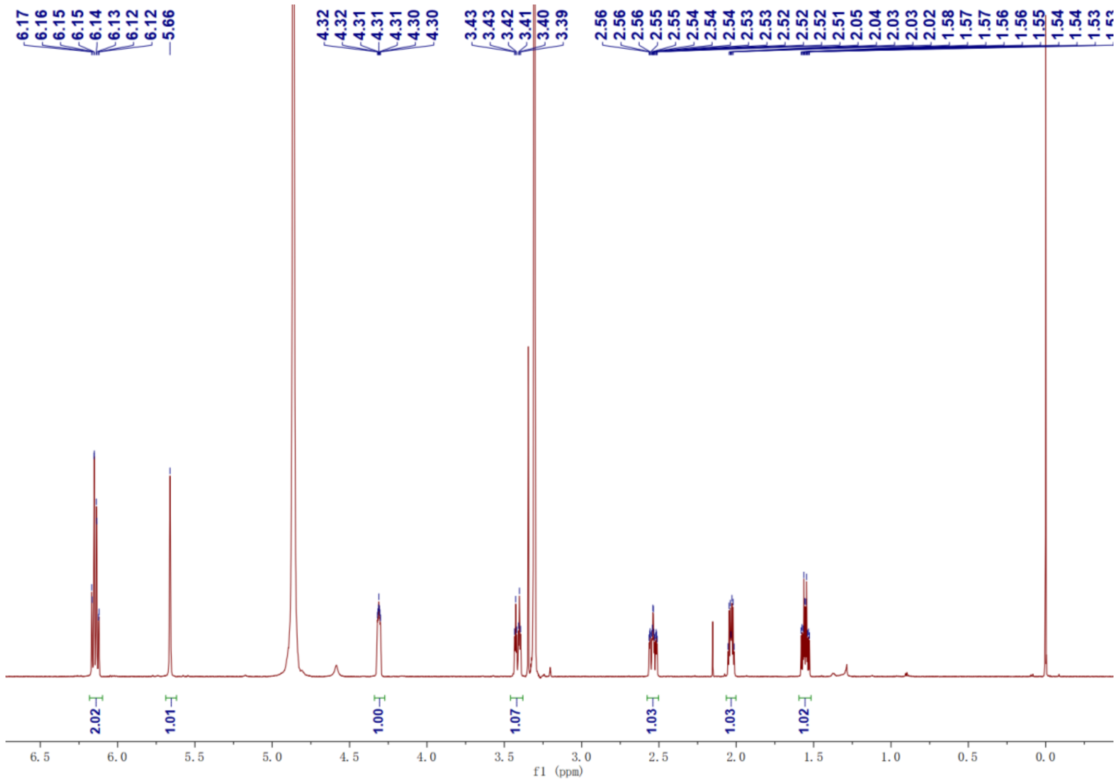

Fig. S12 ^{13}C NMR spectrum (CD_3OD, 176 MHz) of compound **1**

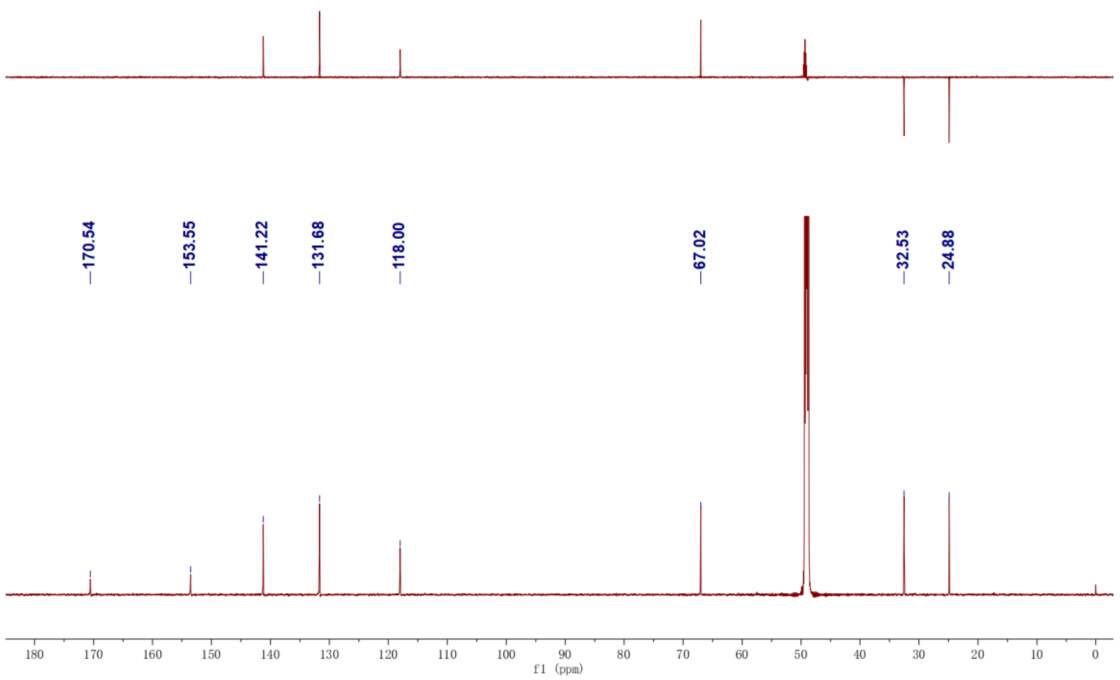

Fig. S13 HSQC spectrum of compound **1**

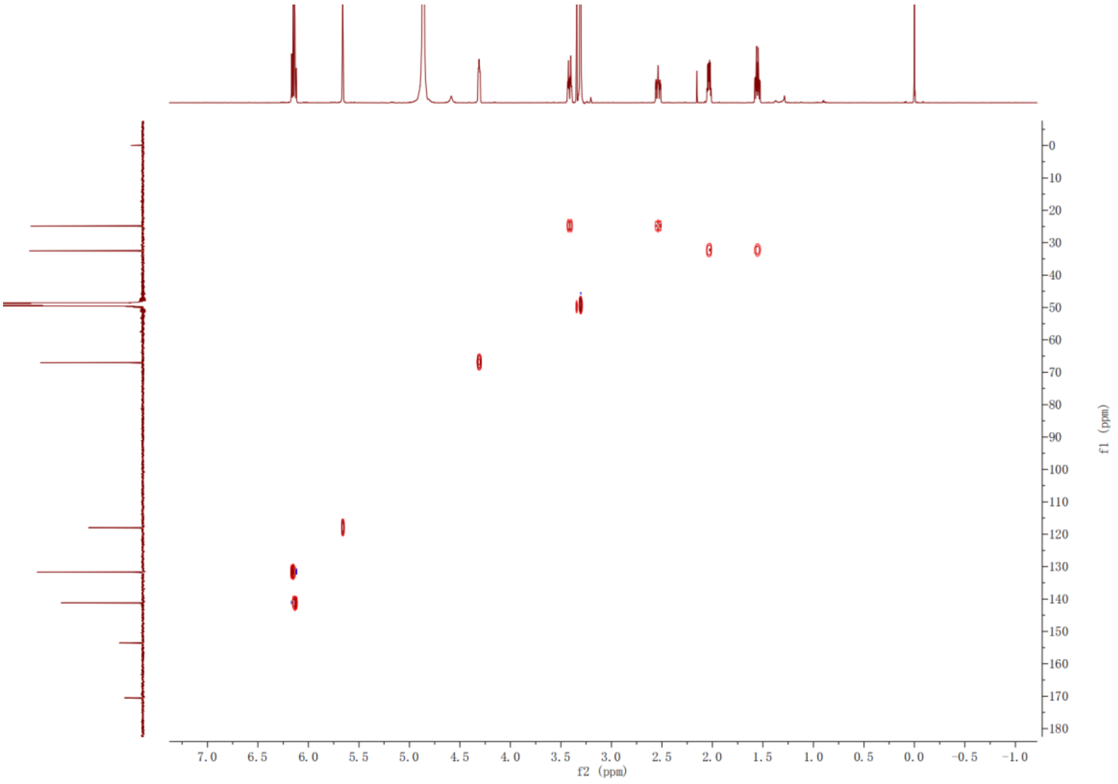

Fig. S14 HMBC spectrum of compound **1**

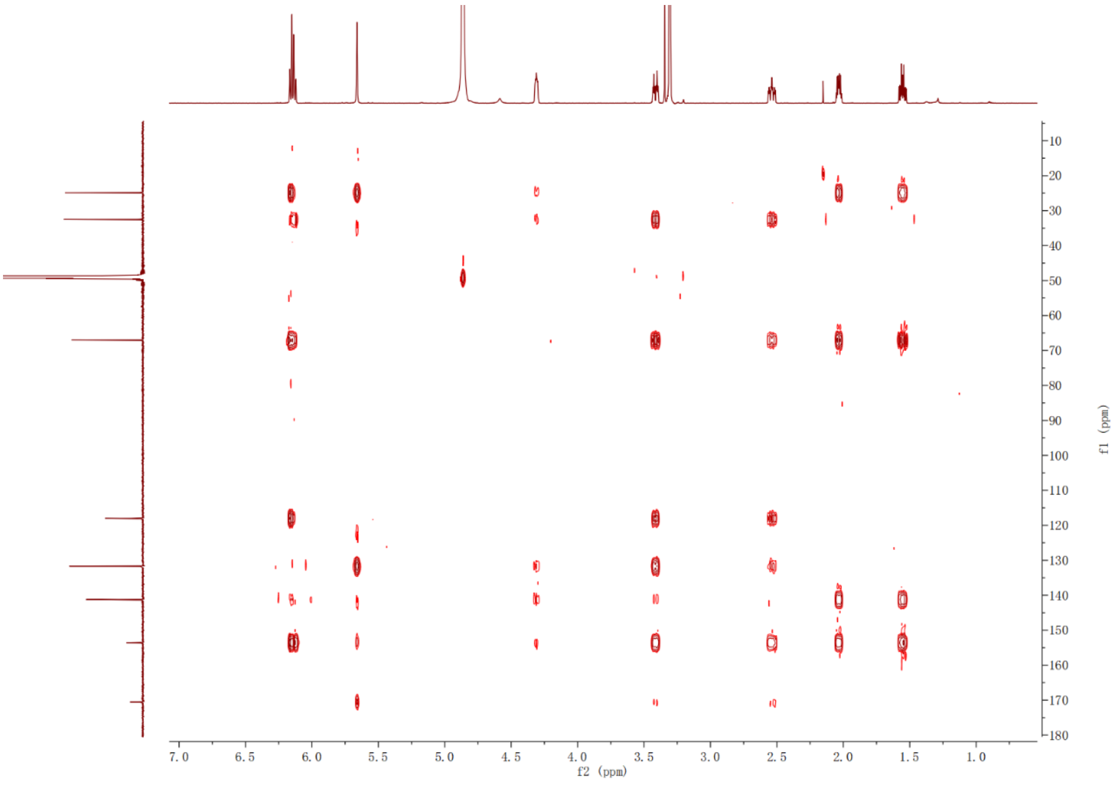

Fig. S15 ^{1}H–^{1}H COSY spectrum of compound **1**

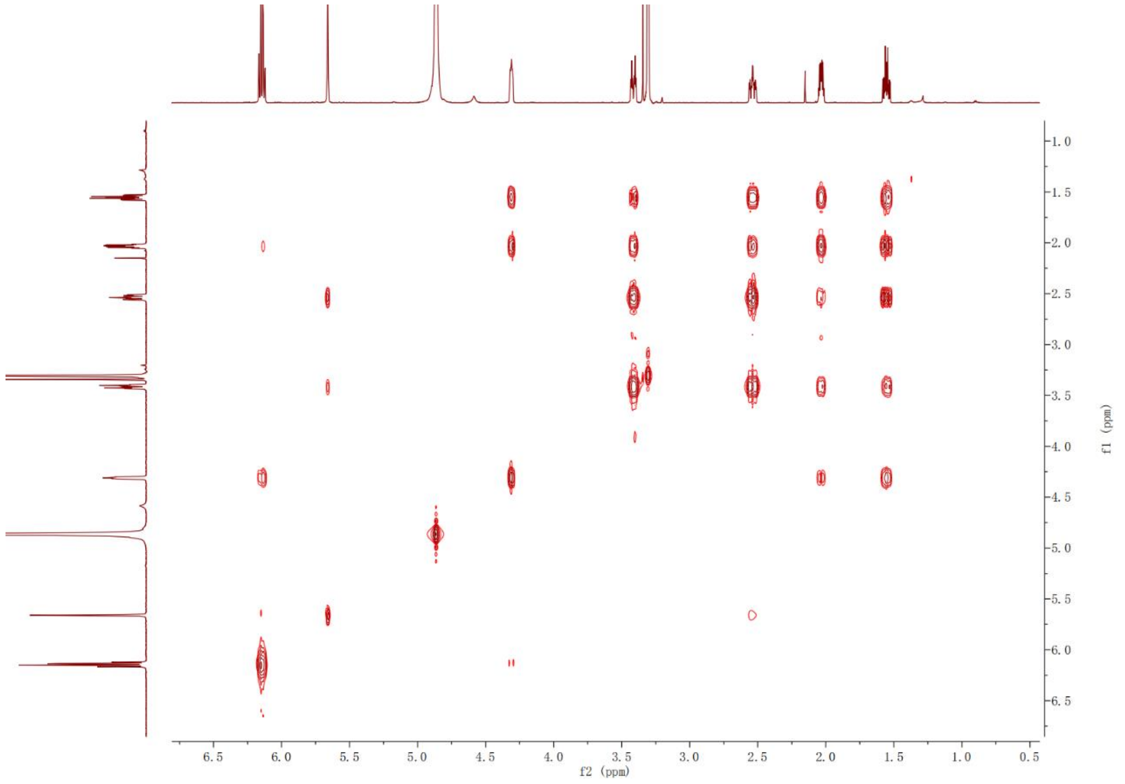

Fig. S16 NOESY spectrum of compound **1**

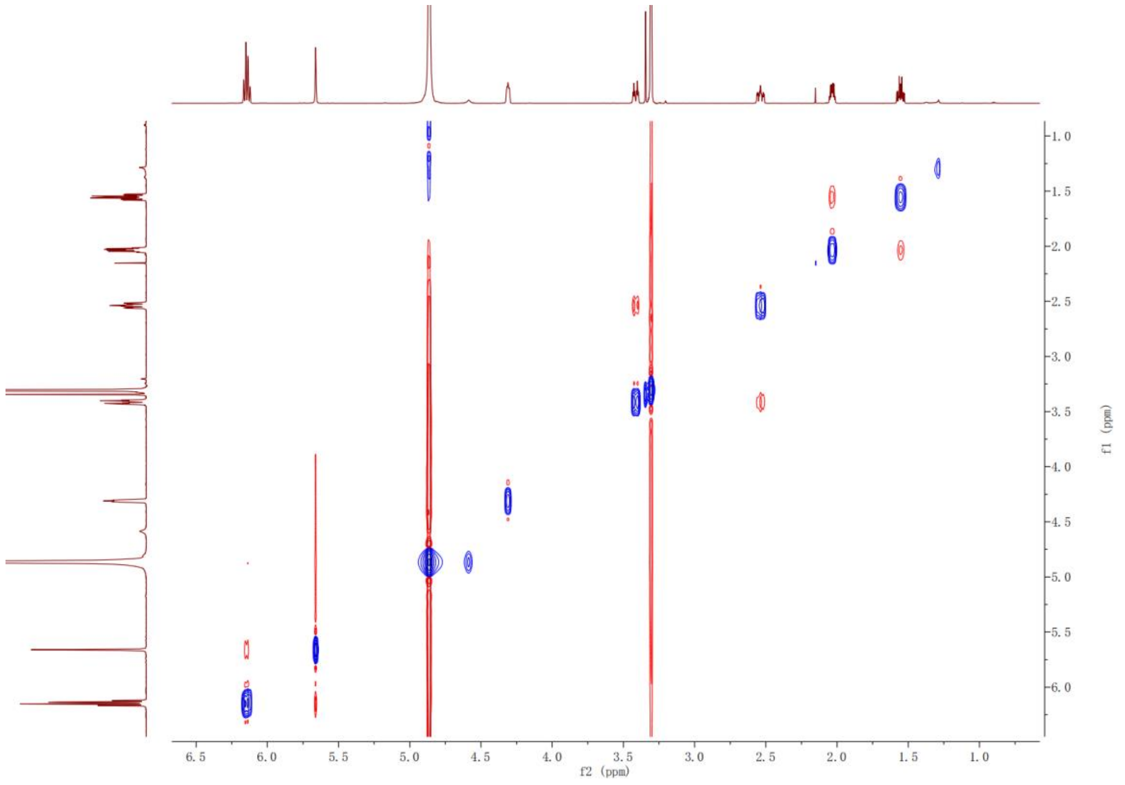

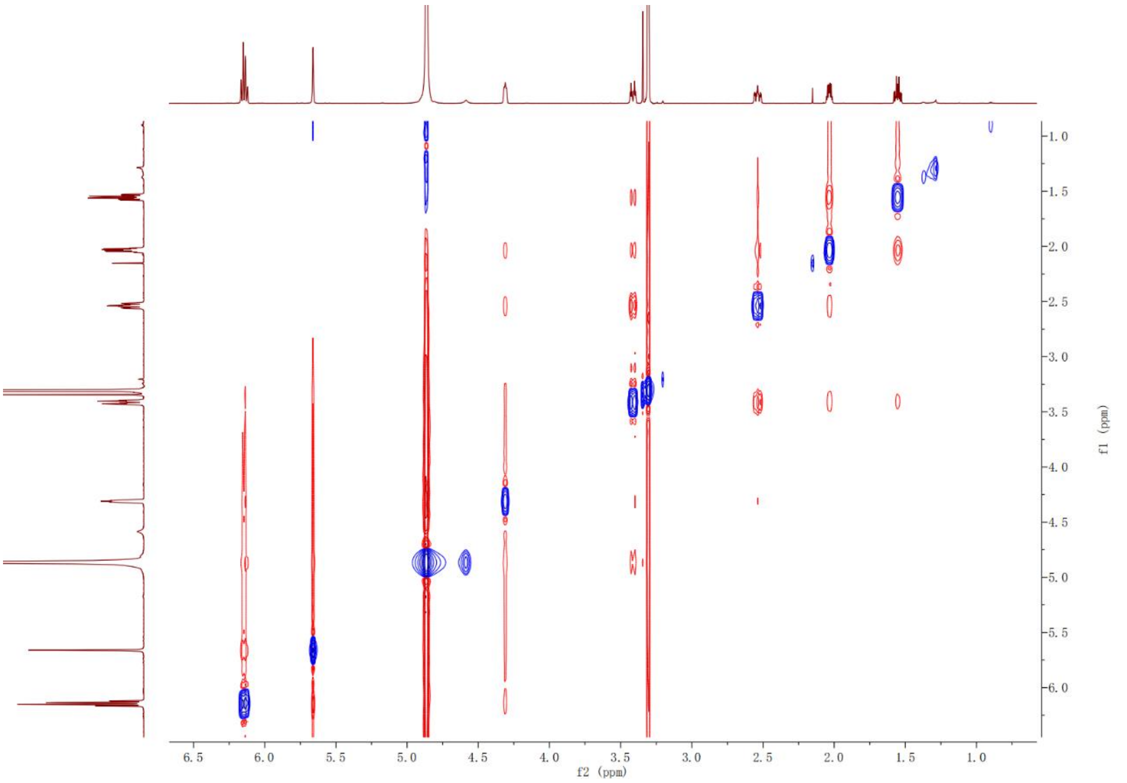

Crystallographic data of compound **1**

Crystal Data for $C_{16}H_{20}O_6$ (*M* =308.32 g/mol): triclinic, space group P1 (no. 1), a = 6.0982(3) Å, b = 7.5360(3) Å, c = 9.0518(3) Å, α = 71.293(3)°, β = 70.575(4)°, γ = 80.927(4)°, V = 370.97(3) Å^3, Z = 1, T = 100.00(10) K, μ(Cu Kα) = 0.883 mm^{-1}, *Dcalc* = 1.380 g/cm^3, 6840 reflections measured (10.822° ≤ 2Θ ≤ 149.022°), 2732 unique (R_{int} = 0.0350, R_{sigma} = 0.0384) which were used in all calculations. The final R_1 was 0.0724 (I > 2σ(I)) and wR_2 was 0.1809 (all data).

Fig. S17 UV spectrum of compound **1**

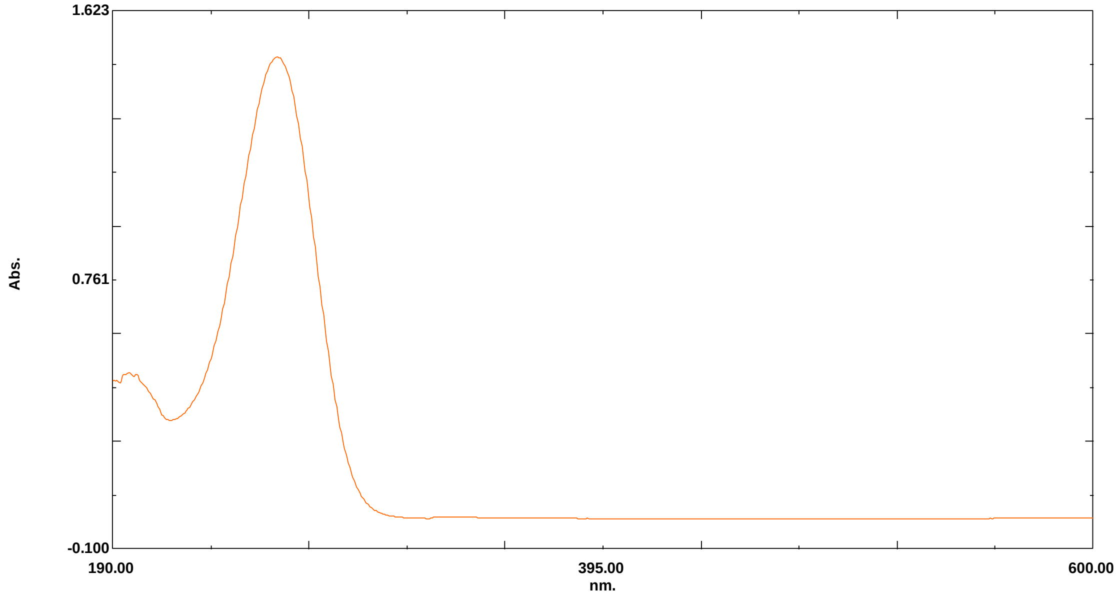

No.	wavelength (nm)	Abs
1	259.00	1.475
2	197.00	0.465

Fig. S18 ECD spectrum of compound **1**

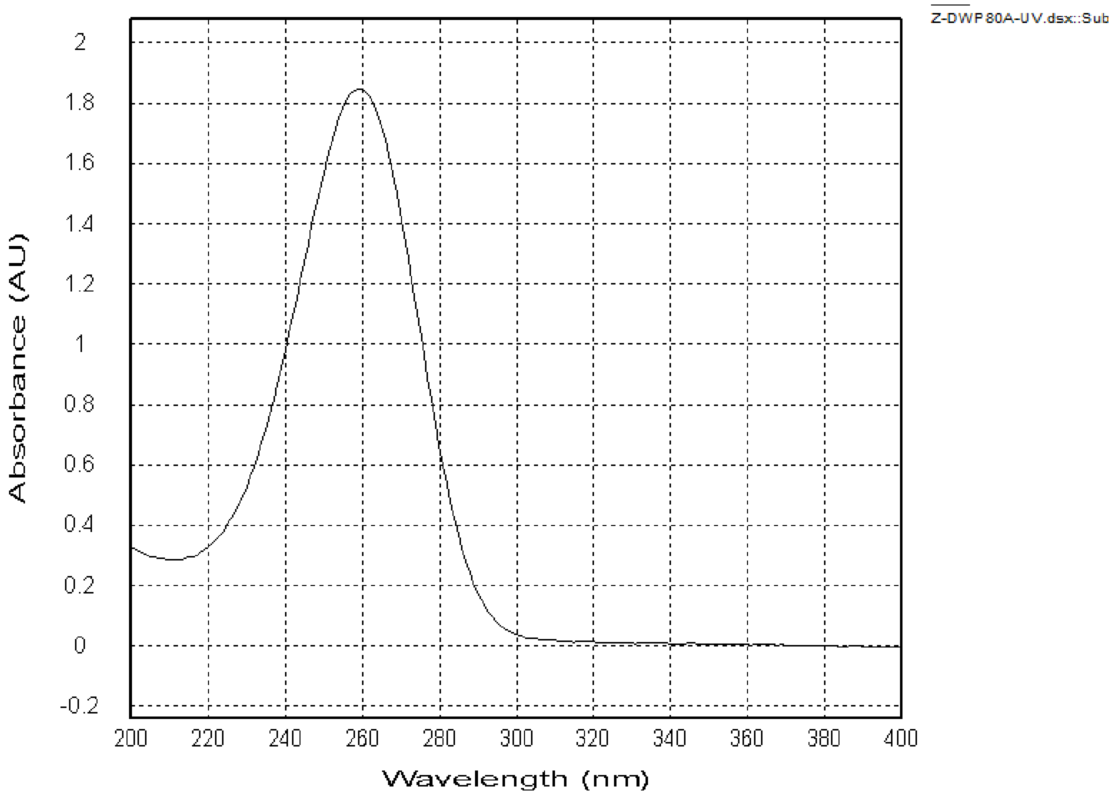

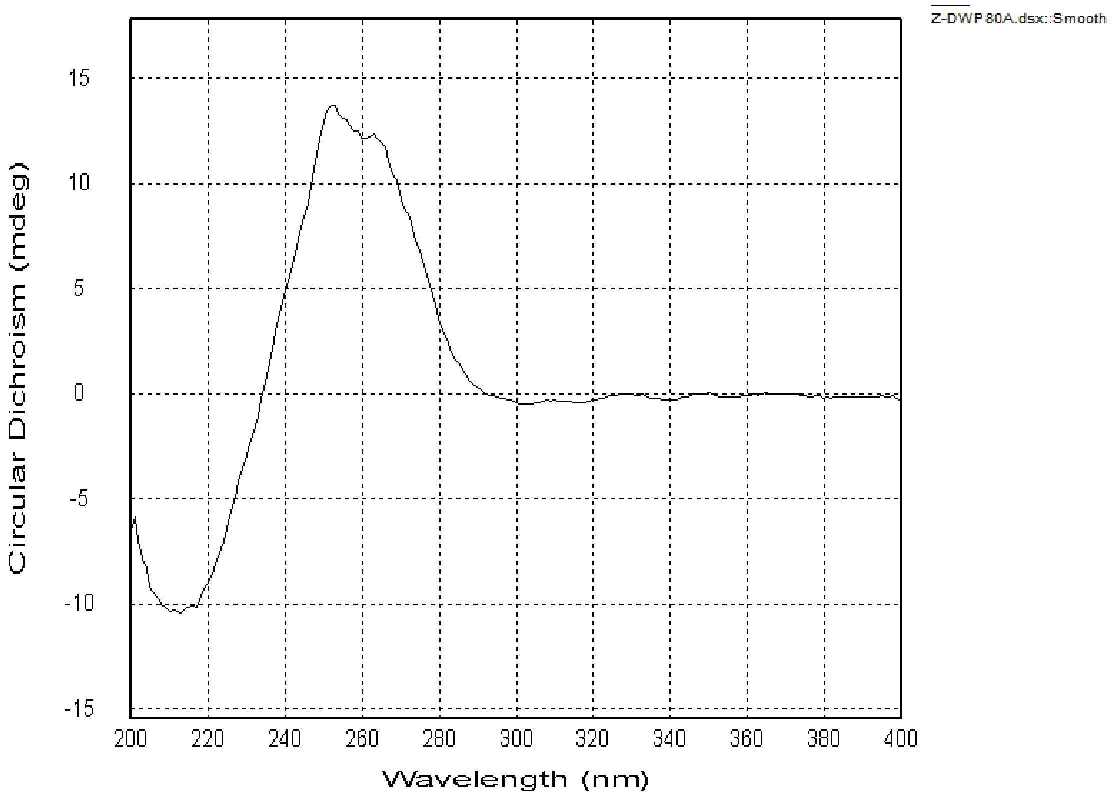

Fig. S19 IR spectrum of compound **1**

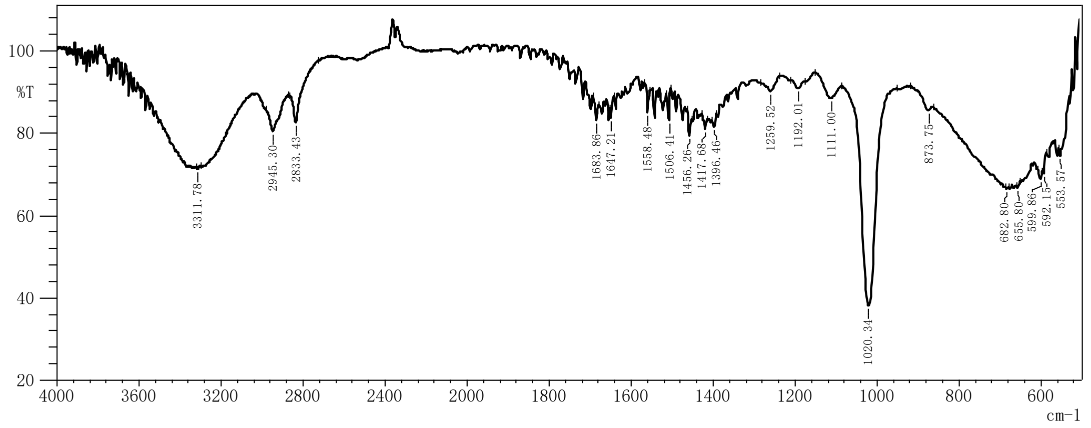

Fig. S20 HRESIMS spectrum of compound **2**

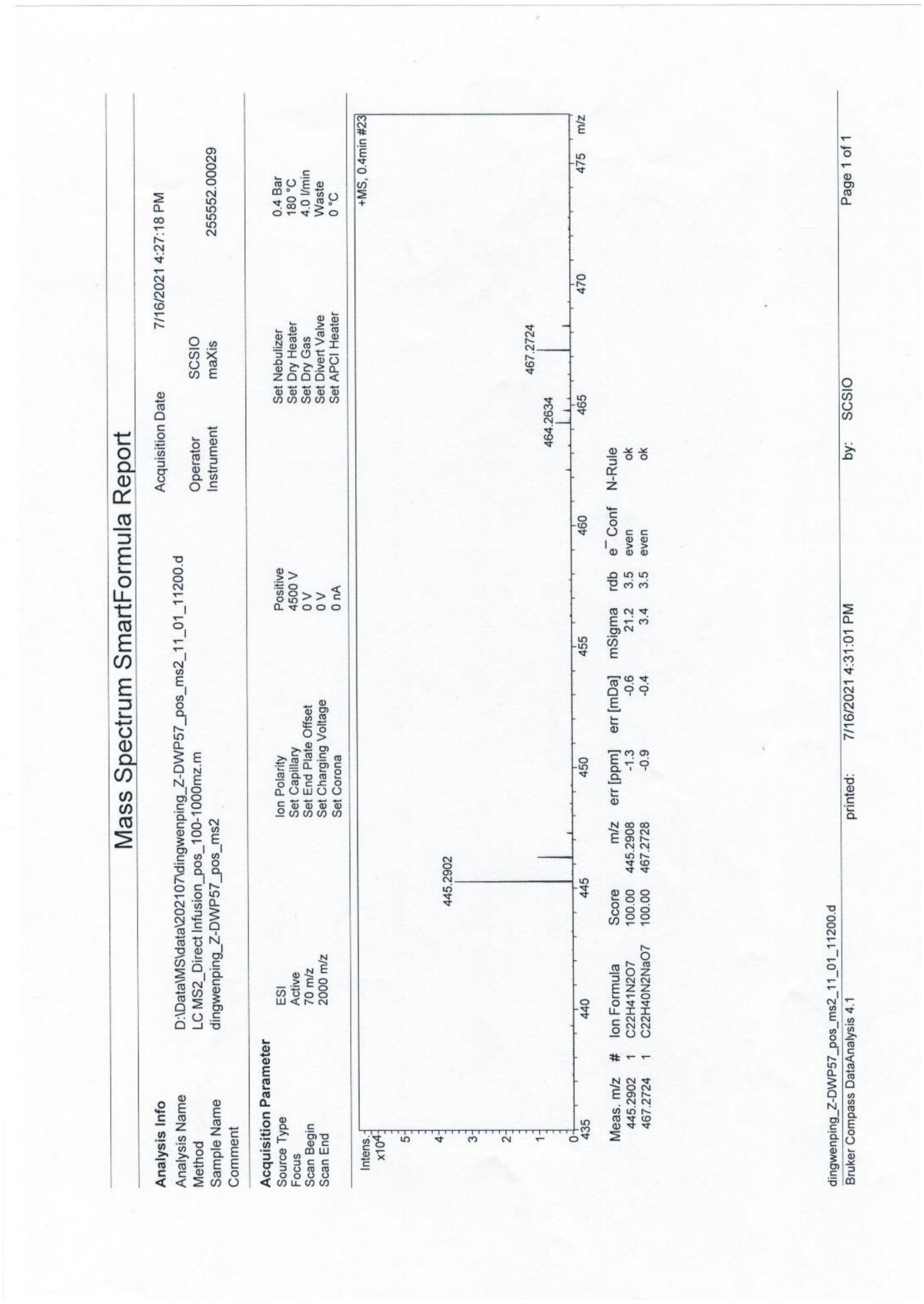

Fig. S21 HRESI-MS/MS-positive spectrum of compound **2**

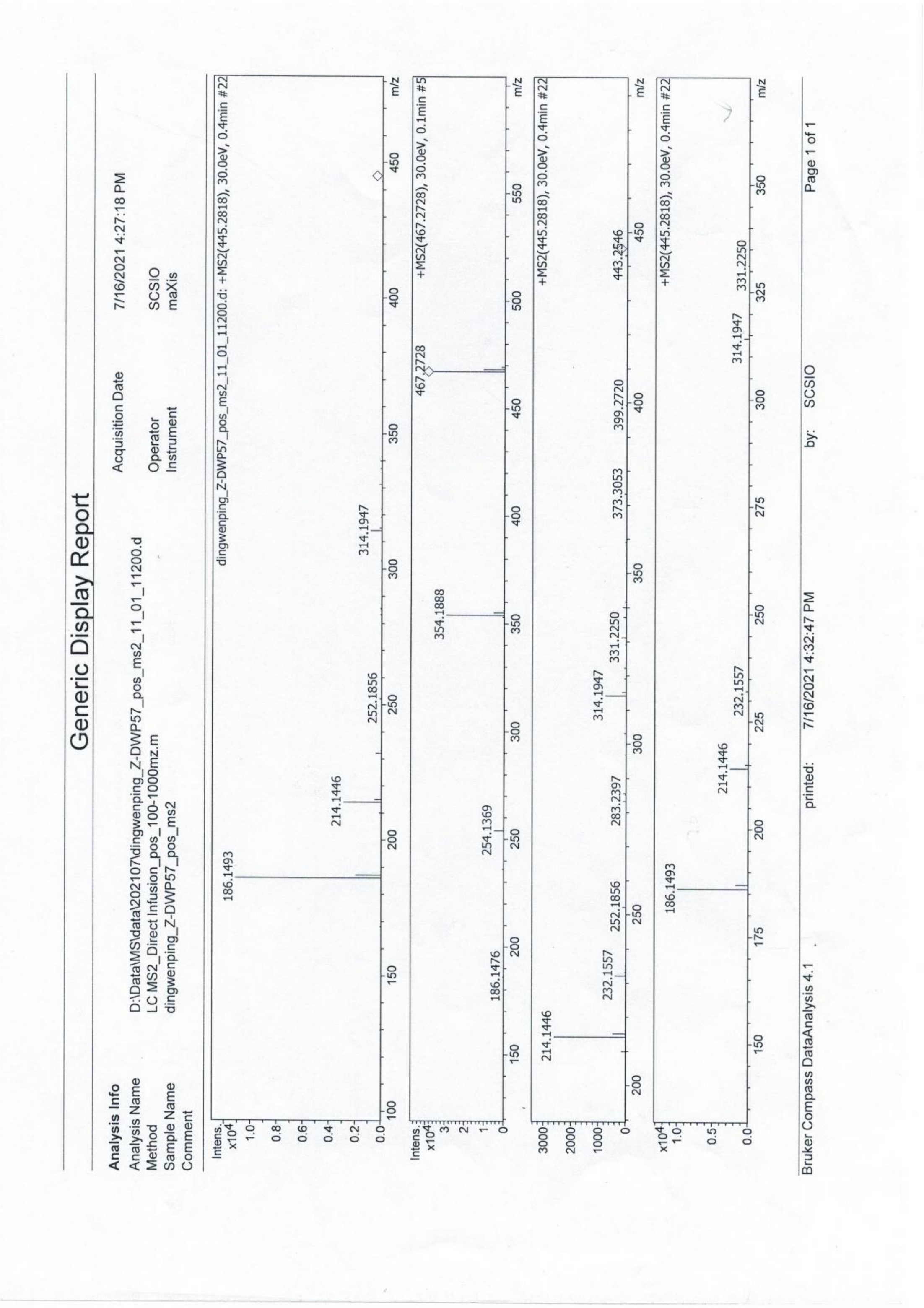

Fig. S22 ^{1}H NMR spectrum (CD_3OD, 700 MHz) of compound **2**

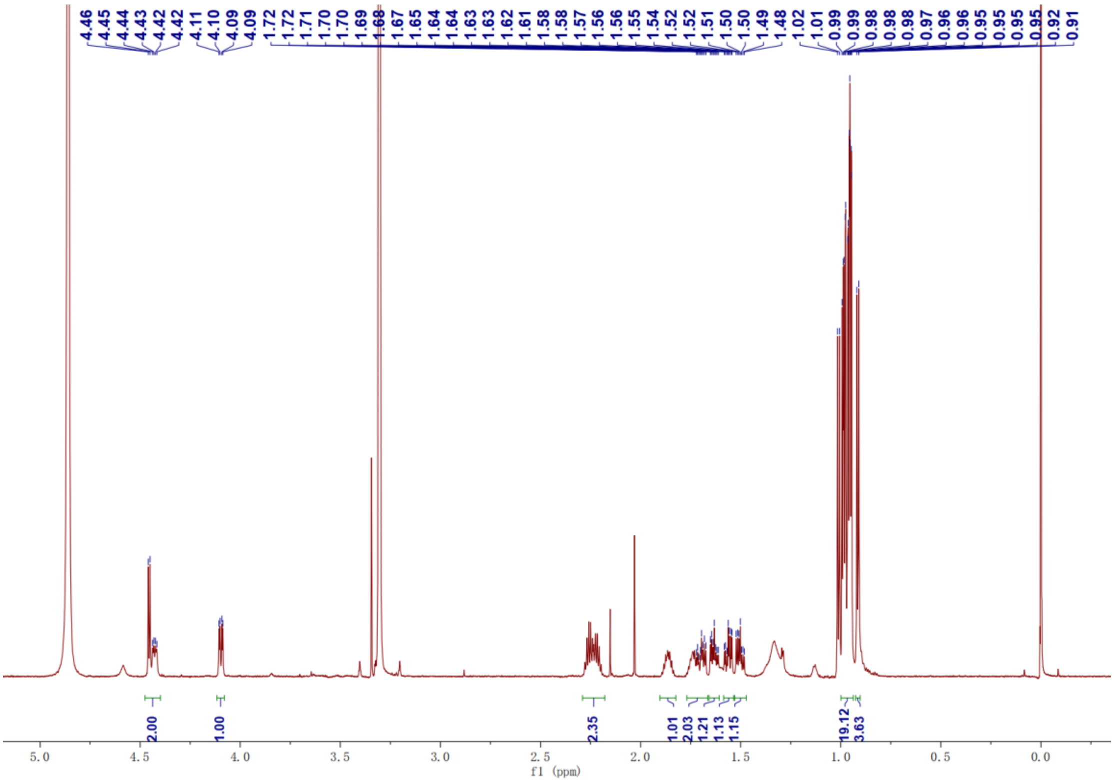

Fig. S23 ^{13}C NMR spectrum (CD_3OD, 176 MHz) of compound **2**

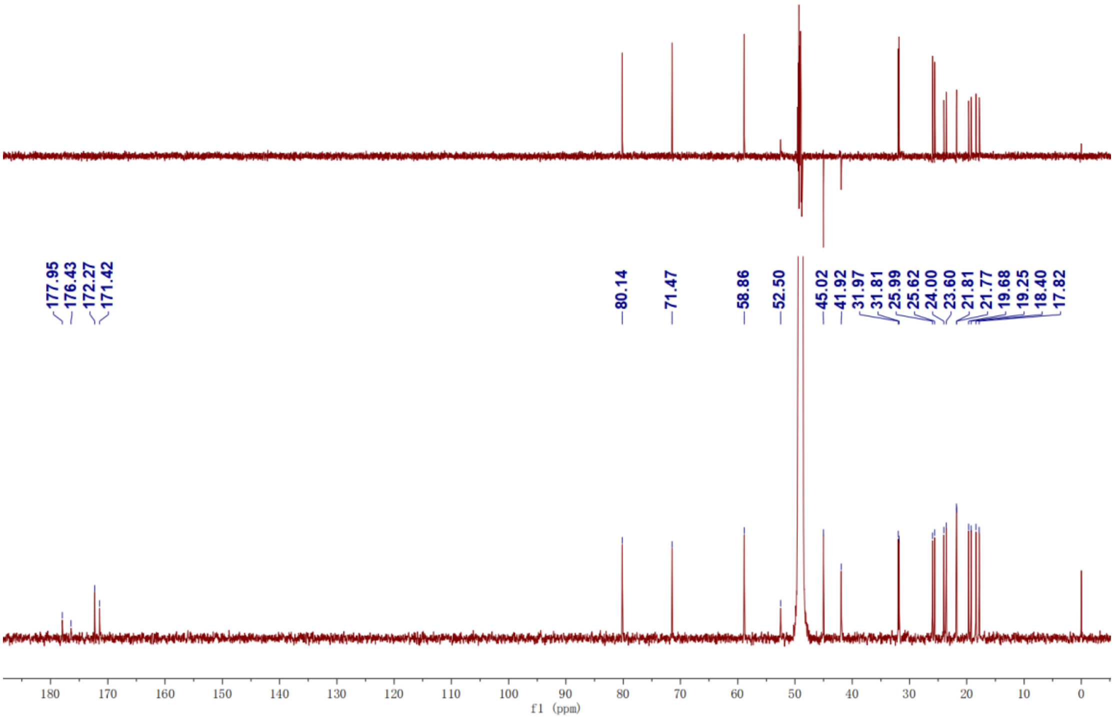

Fig. S24 HSQC spectrum of compound **2**

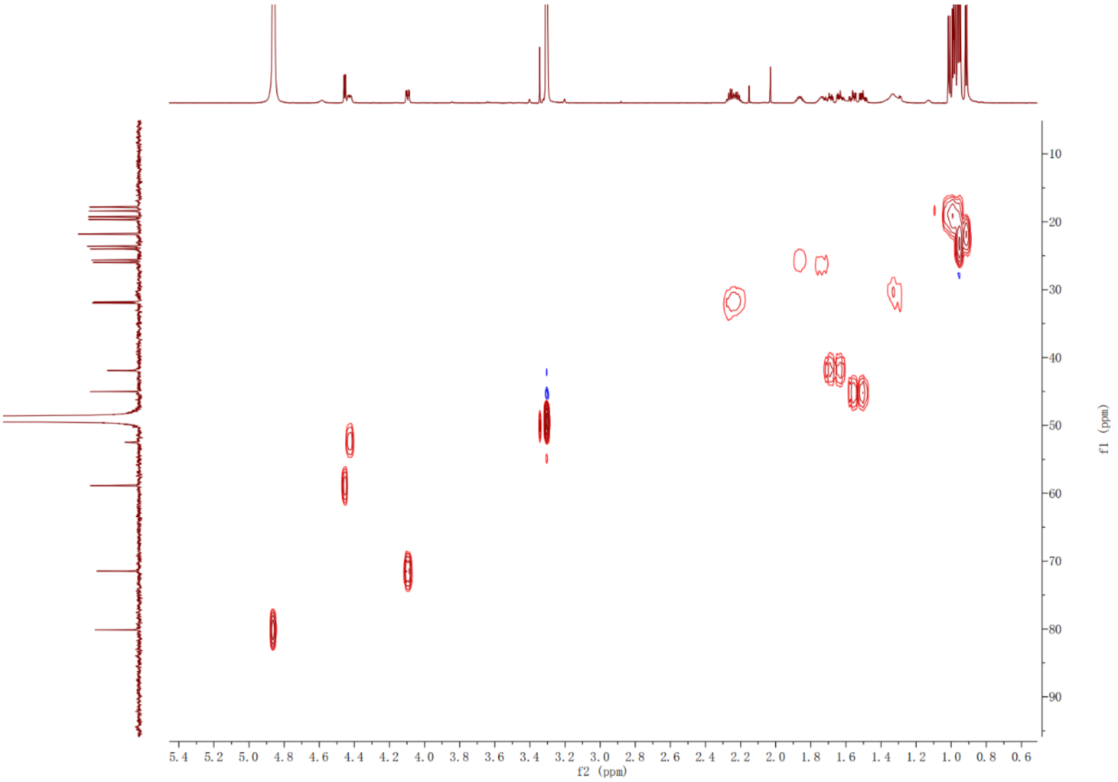

Fig. S25 HMBC spectrum of compound **2**

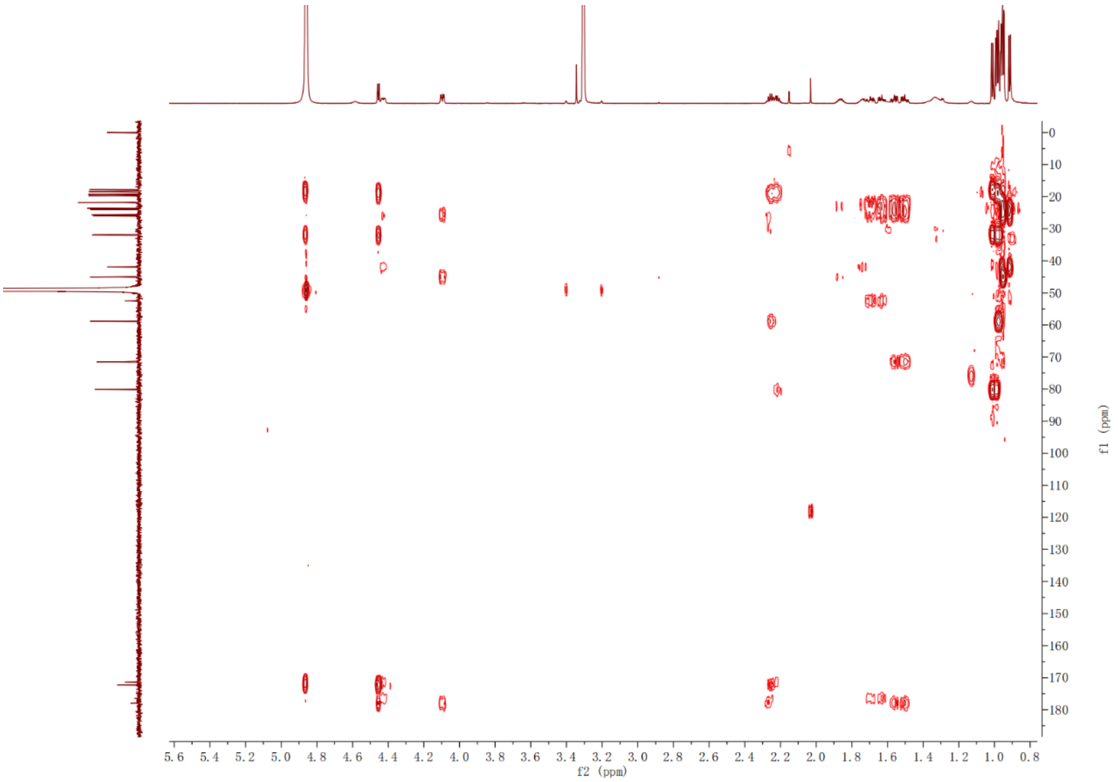

Fig. S26 ^{1}H–^{1}H COSY spectrum of compound **2**

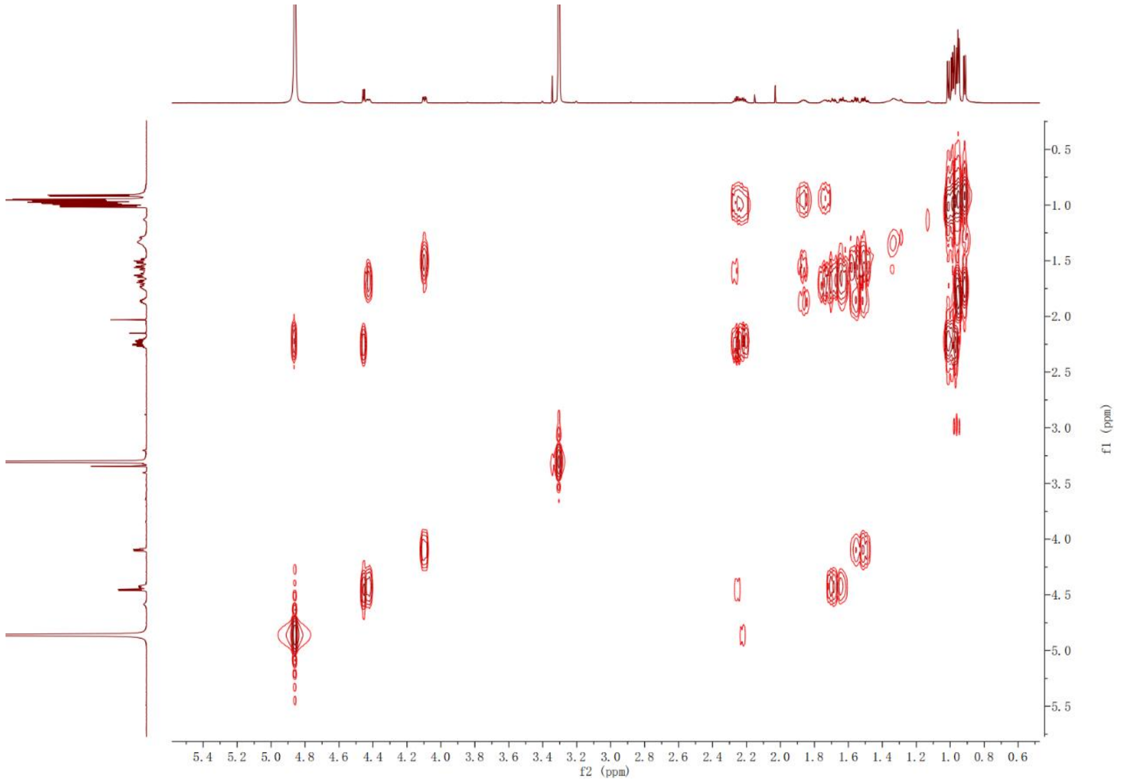

Fig. S27 NOESY spectrum of compound **2**

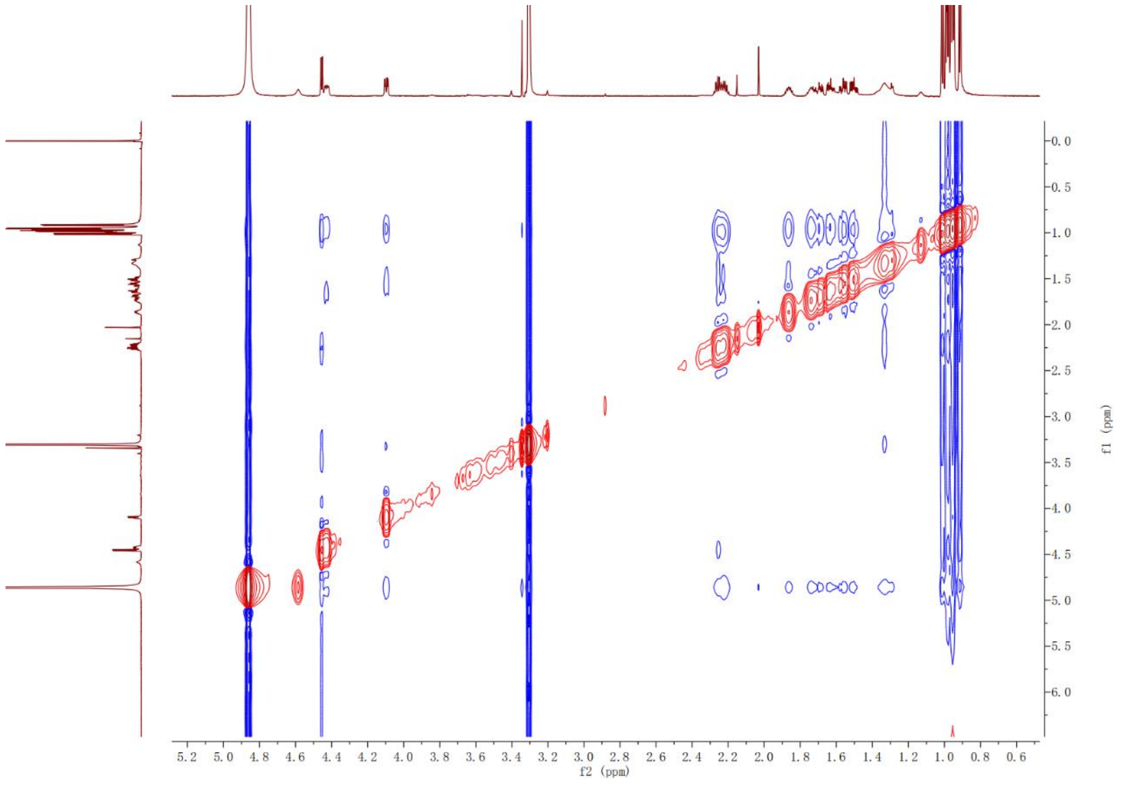

Fig. S28 UV spectrum of compound **2**

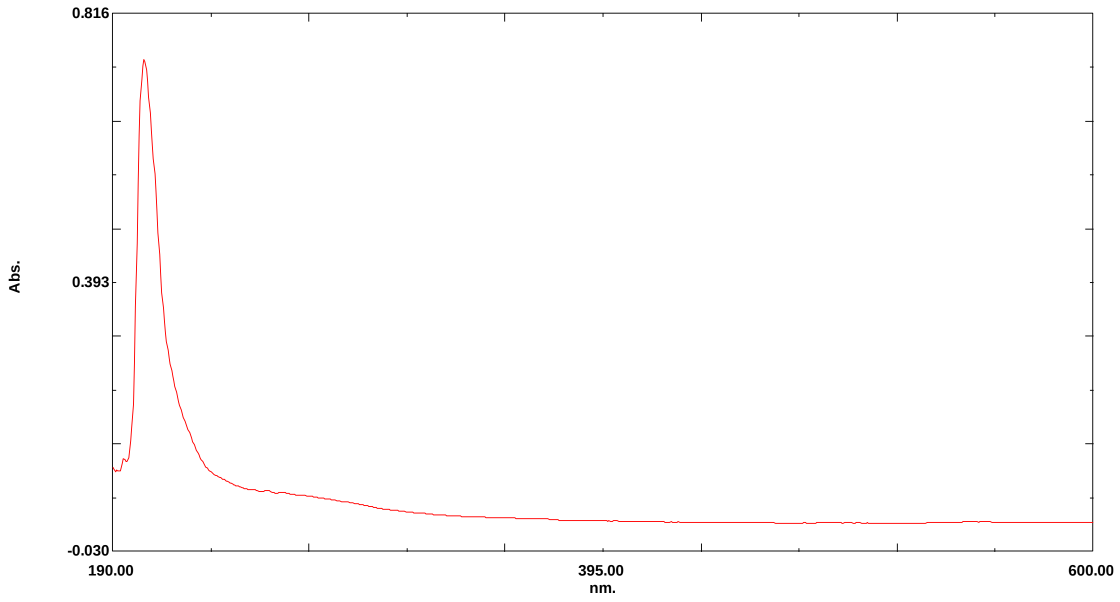

No.	wavelength (nm)	Abs
1	203.00	0.743

Fig. S29 ECD spectrum of compound **2**

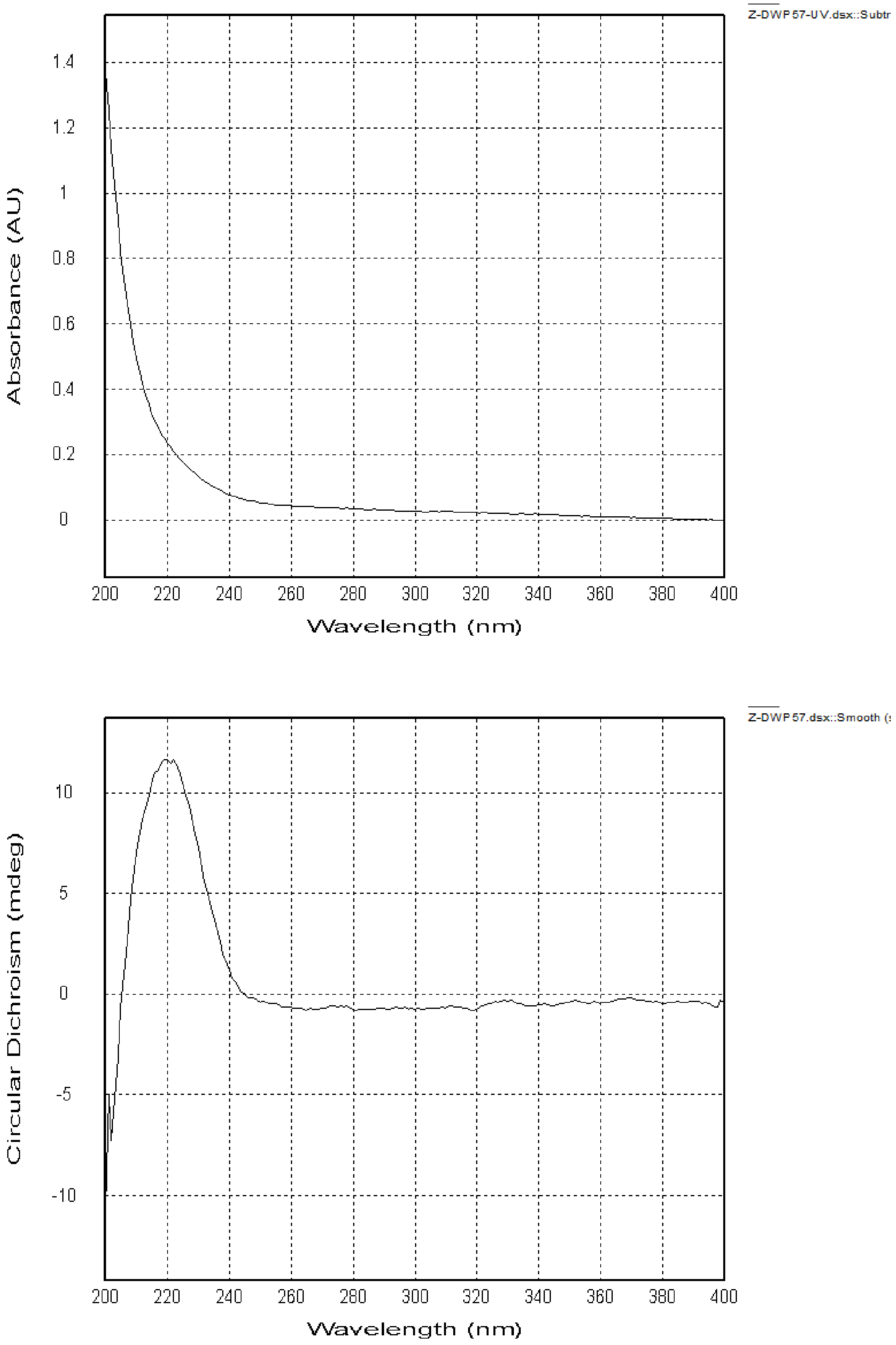

Fig. S30 IR spectrum of compound **2**

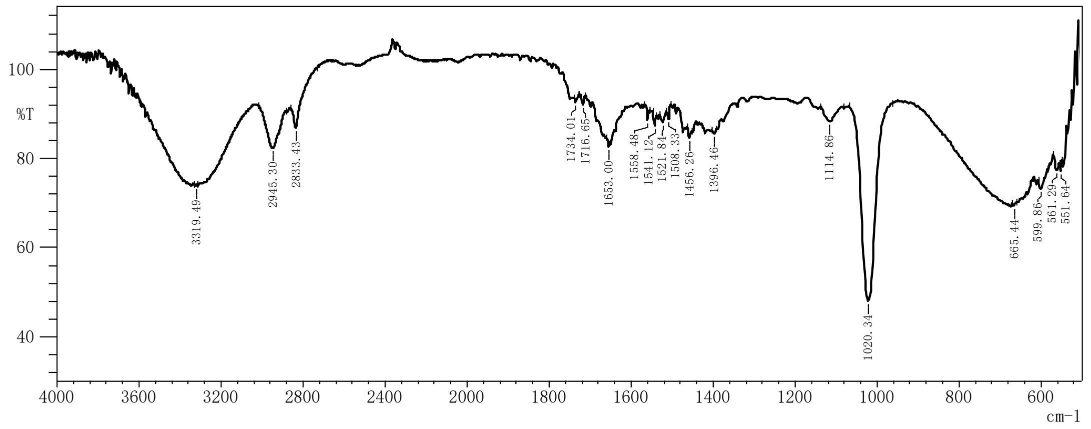

Fig. S31 HRESIMS spectrum of compound **3**

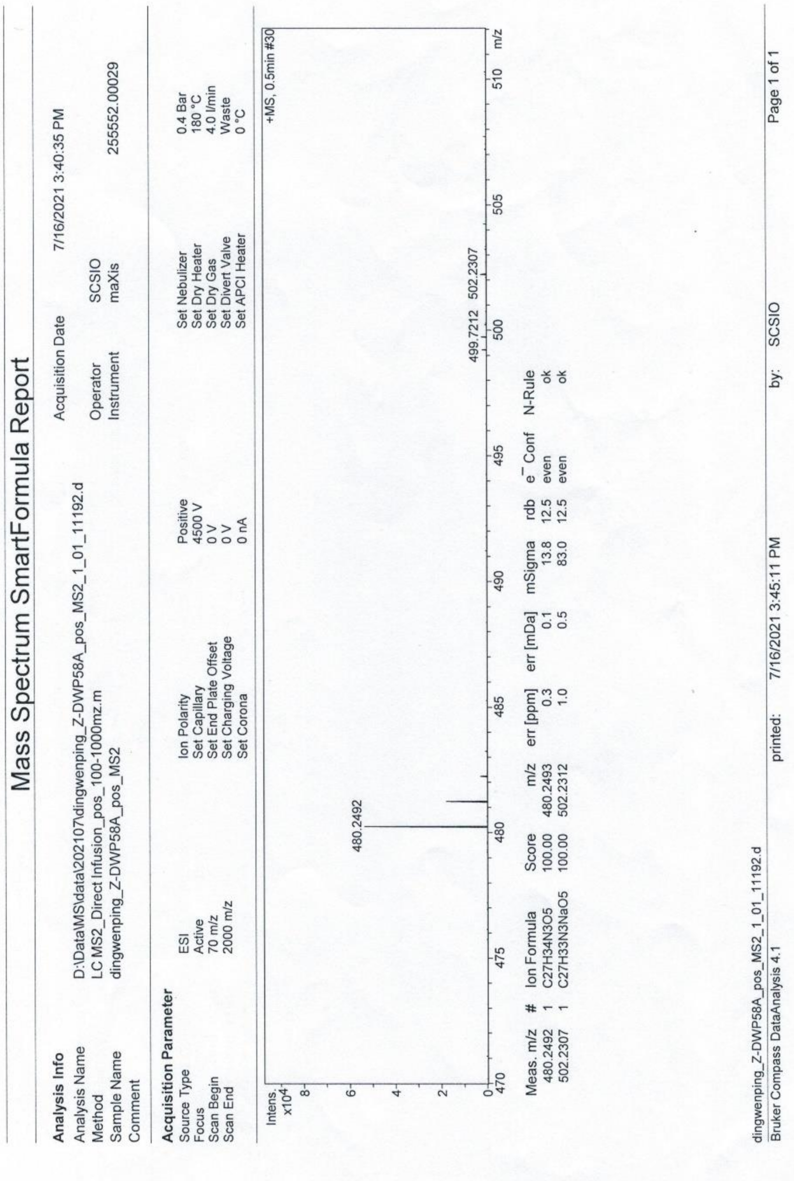

Meas. m/z	#	Ion Formula	Score	m/z	err [ppm]	err [mDa]	mSigma	rdb	e⁻ Conf	N-Rule
480.2492	1	C27H34N3O5	100.00	480.2493	0.3	0.1	13.8	12.5	even	ok
502.2307	1	C27H33N3NaO5	100.00	502.2312	1.0	0.5	83.0	12.5	even	ok

dingwenping_Z-DWP58A_pos_MS2_1_01_11192.d
Bruker Compass DataAnalysis 4.1 printed: 7/16/2021 3:45:11 PM by: SCSIO Page 1 of 1

Fig. S32 HRESI-MS/MS-positive spectrum of compound **3**

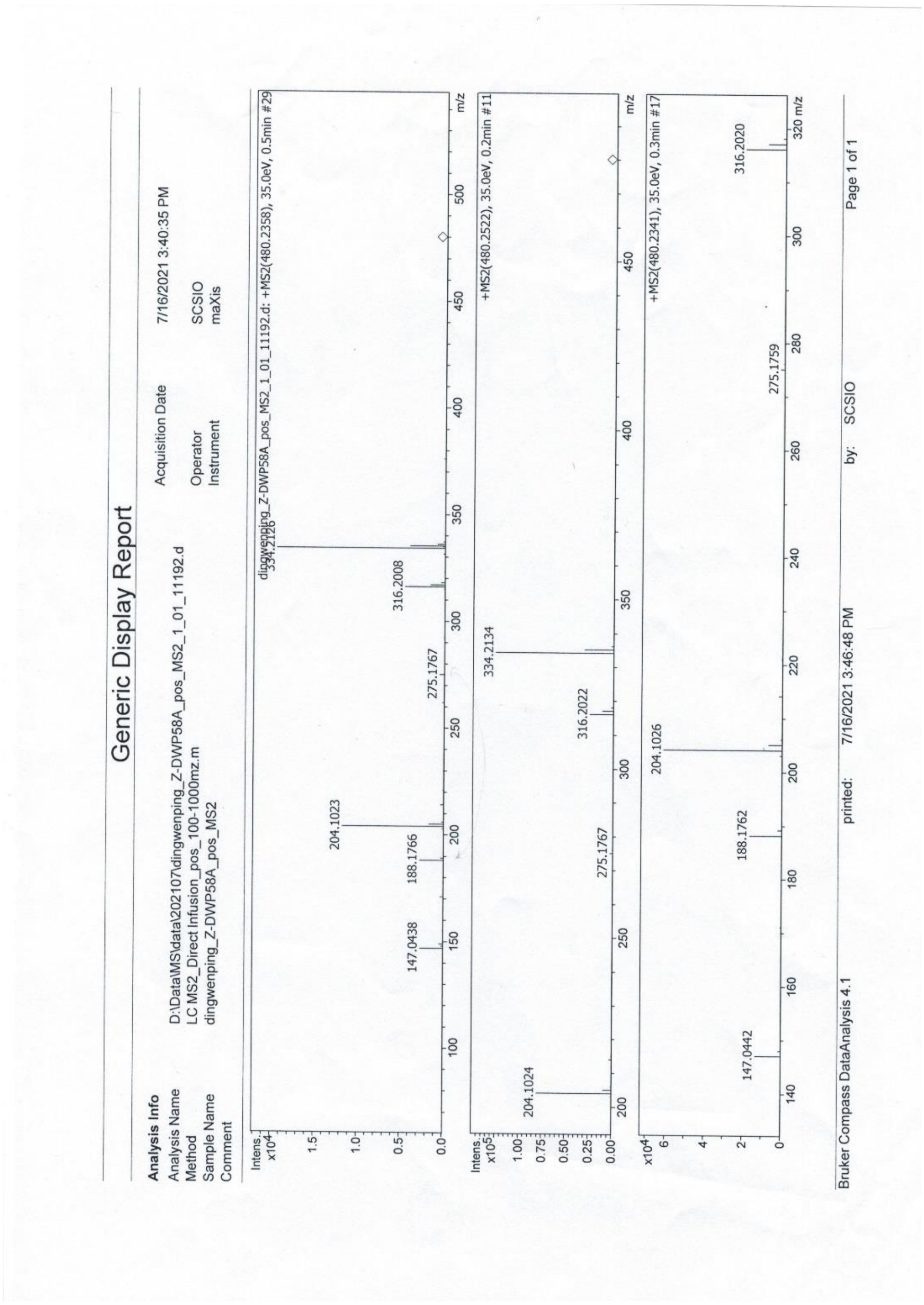

Fig. S33 ^{1}H NMR spectrum (CD_3OD, 700 MHz) of compound **3** (1)

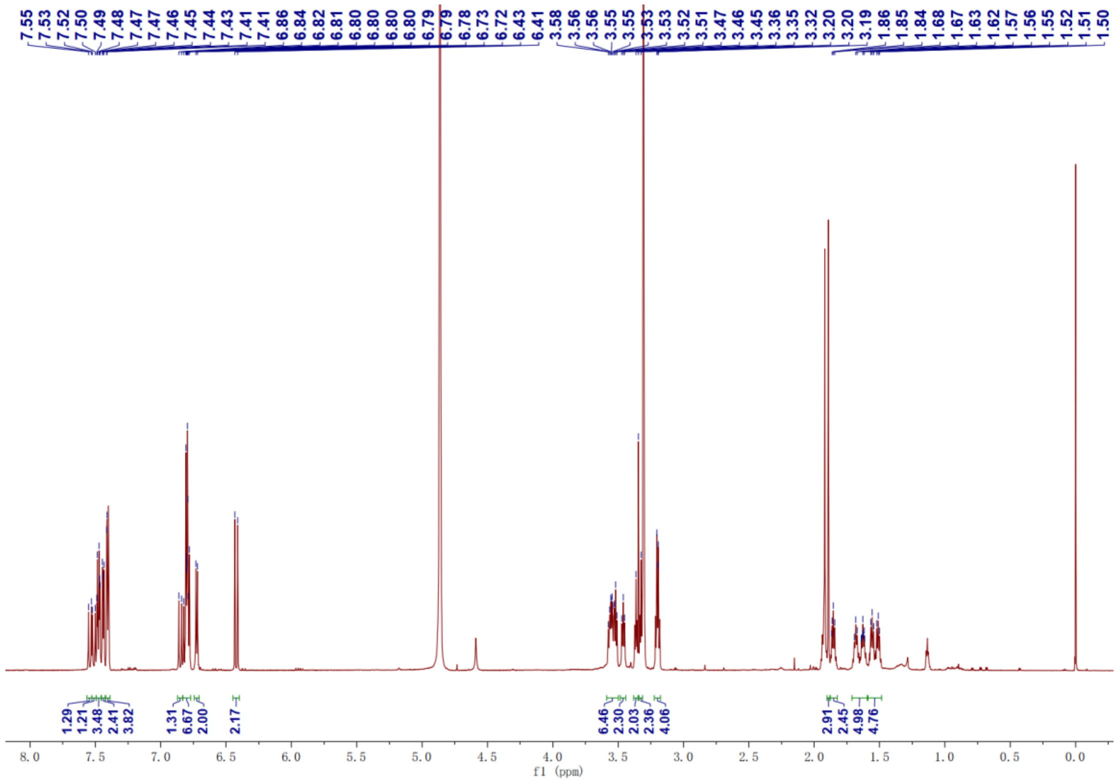

Fig. S34 ^{13}C NMR spectrum (CD_3OD, 176 MHz) of compound **3** (1)

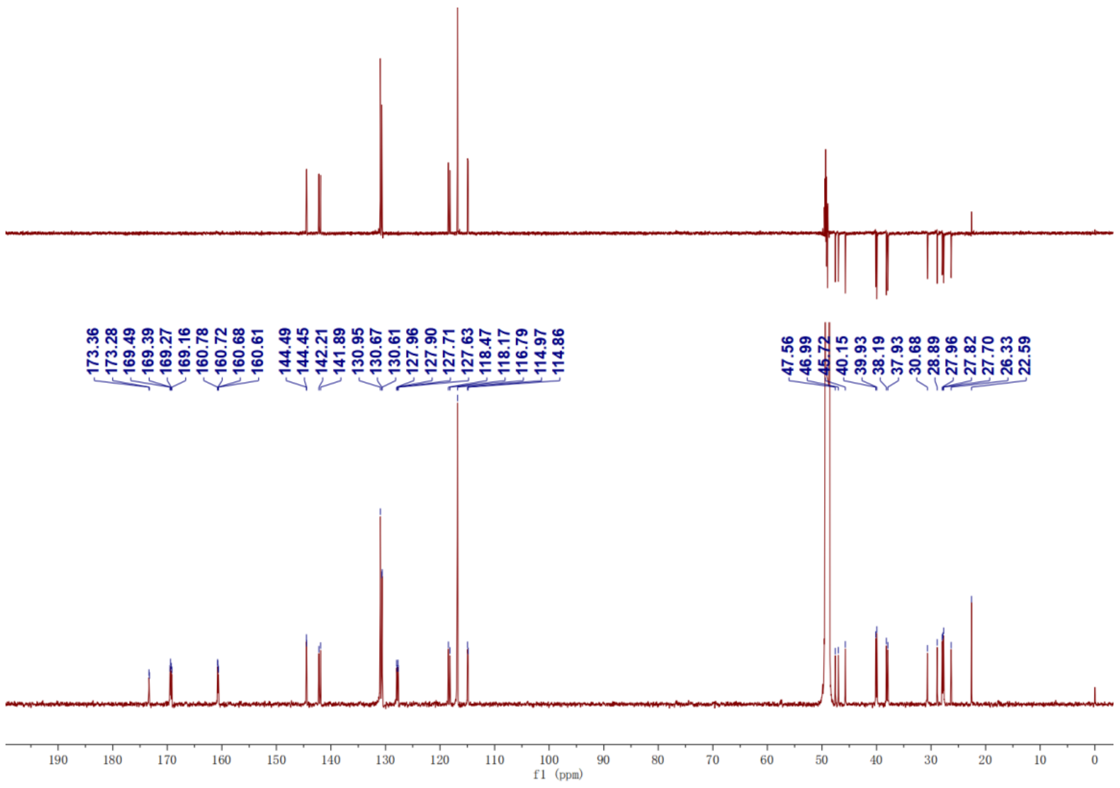

Fig. S35 ^{1}H NMR spectrum (CD_3OD, 700 MHz) of compound **3**

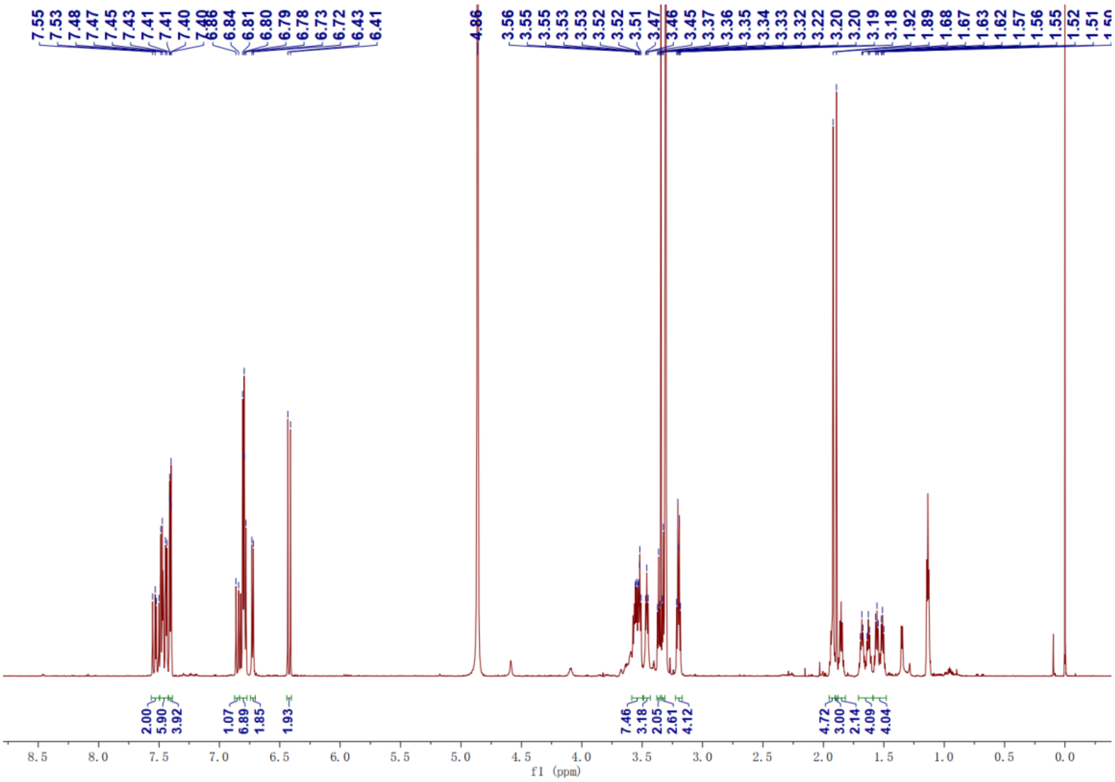

Fig. S36 ^{13}C NMR spectrum (CD_3OD, 176 MHz) of compound **3**

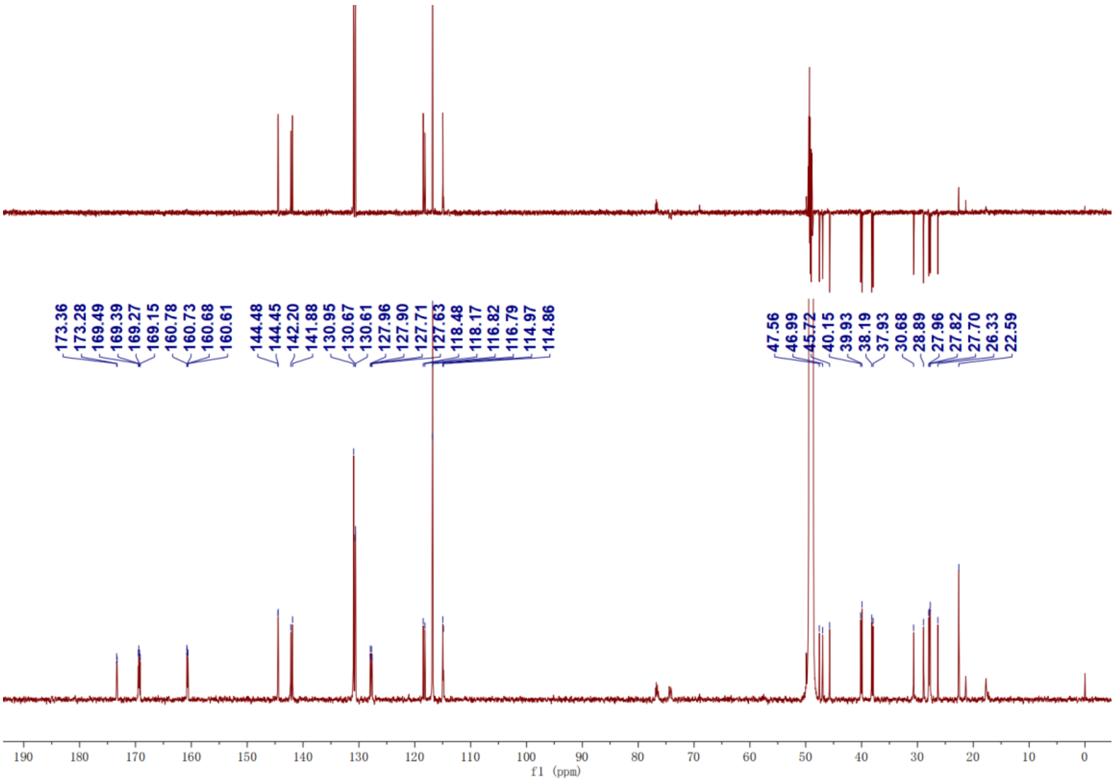

Fig. **S37** HSQC spectrum of compound **3**

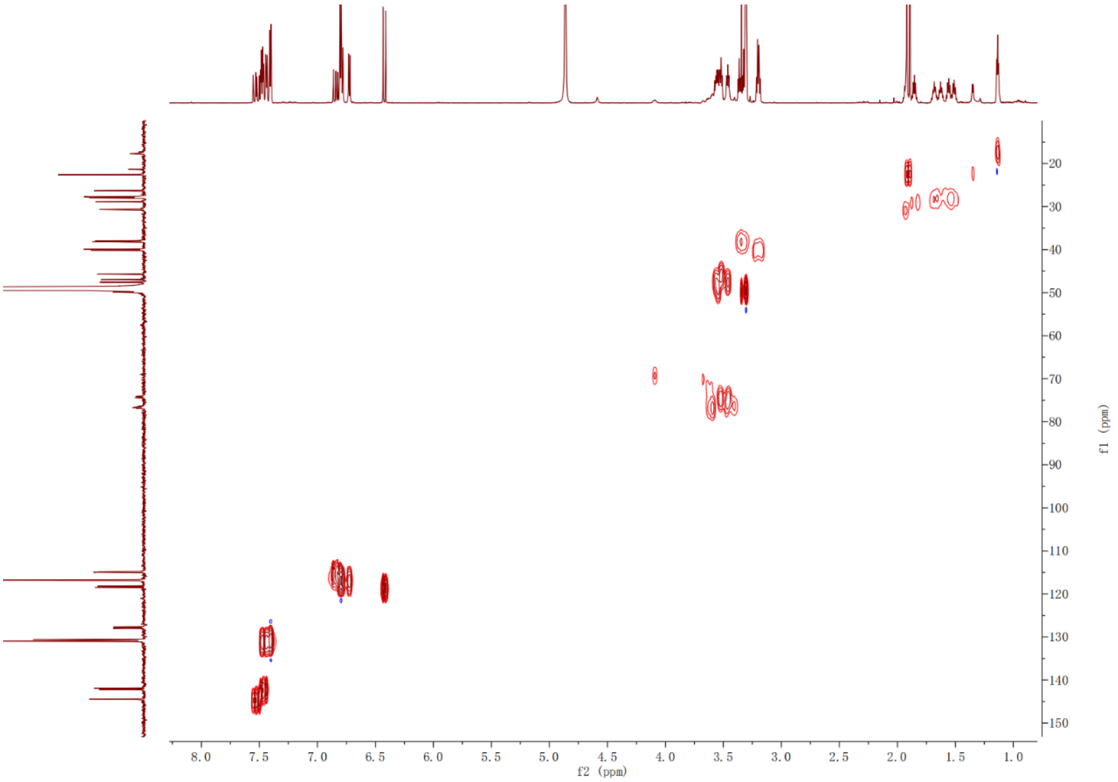

Fig. **S38** HMBC spectrum of compound **3**

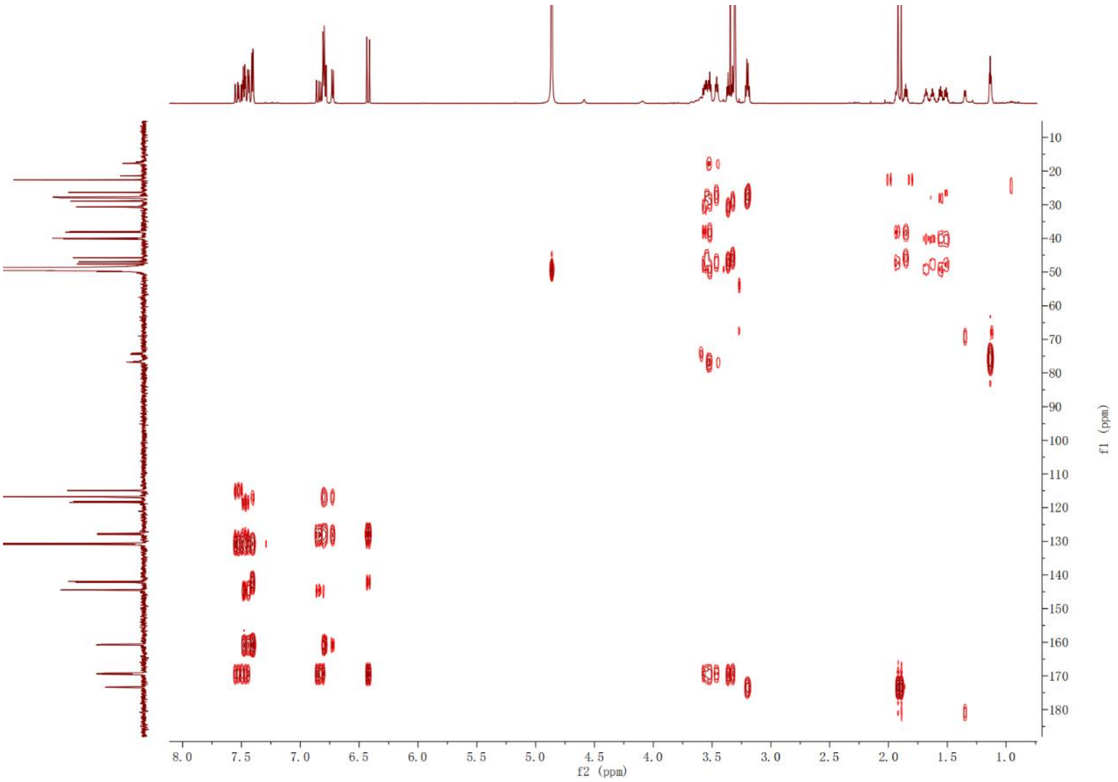

Fig. S39 ^{1}H–^{1}H COSY spectrum of compound **3**

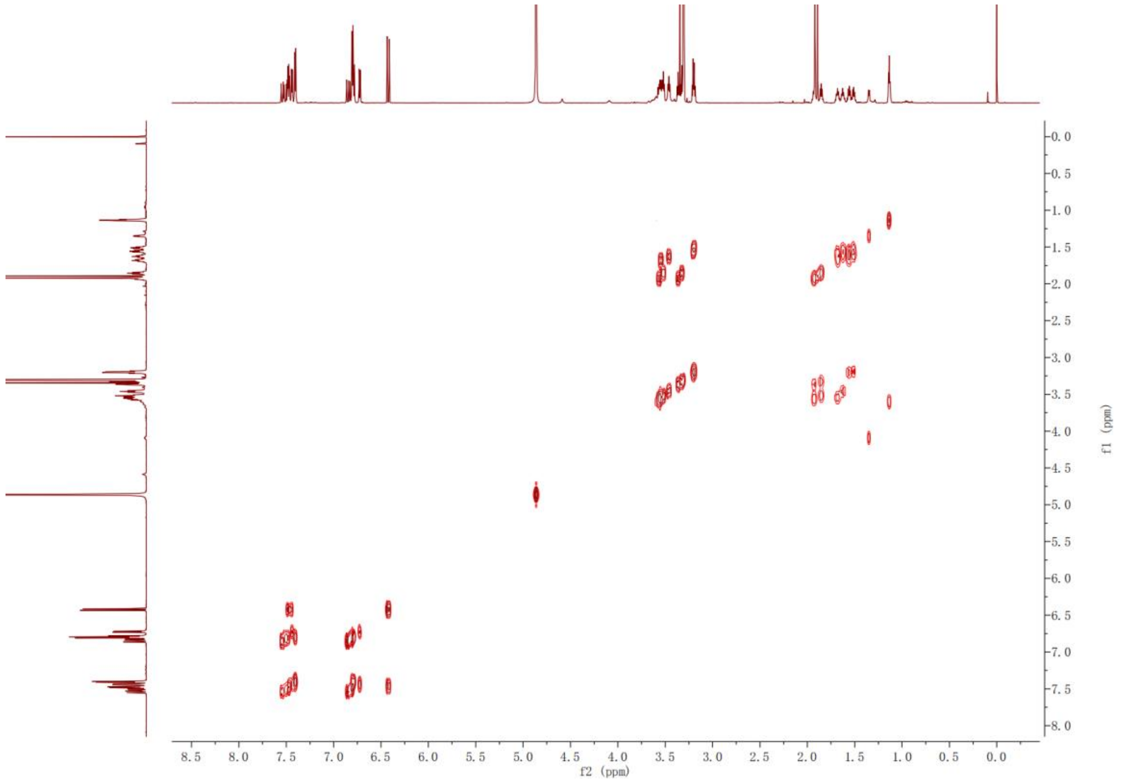

Fig. S40 NOESY spectrum of compound **3**

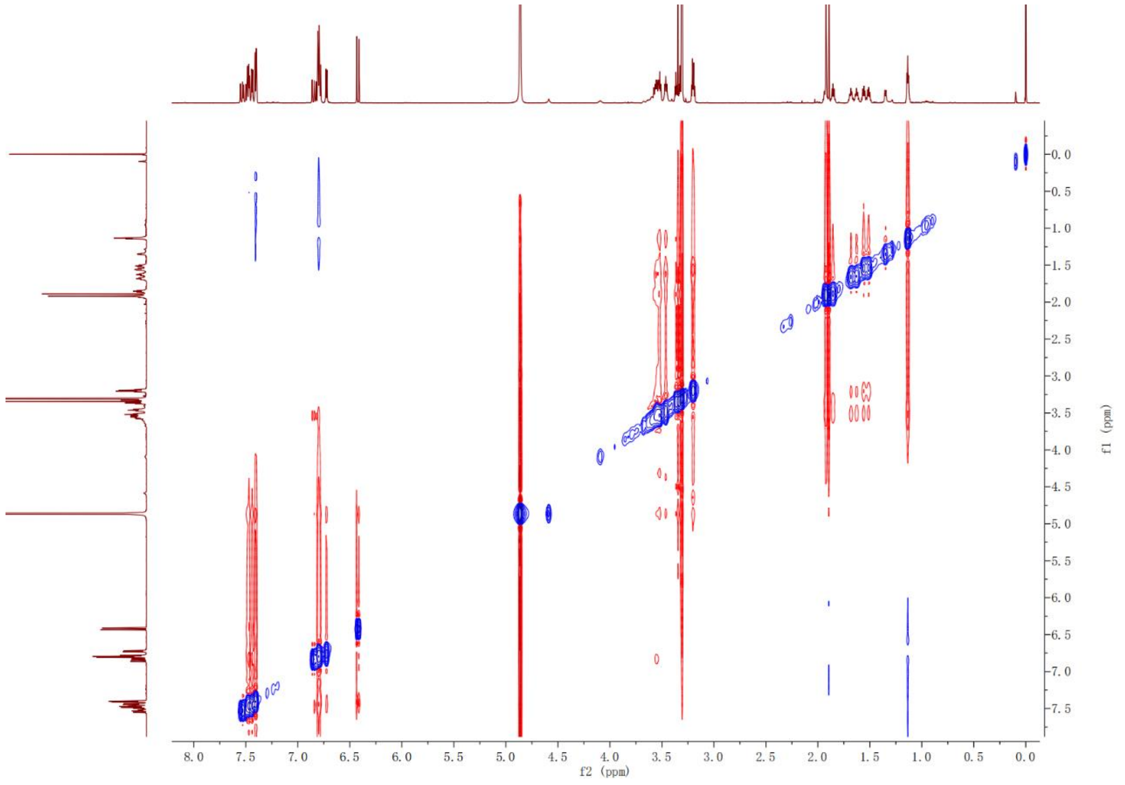

Fig. S41 UV spectrum of compound **3**

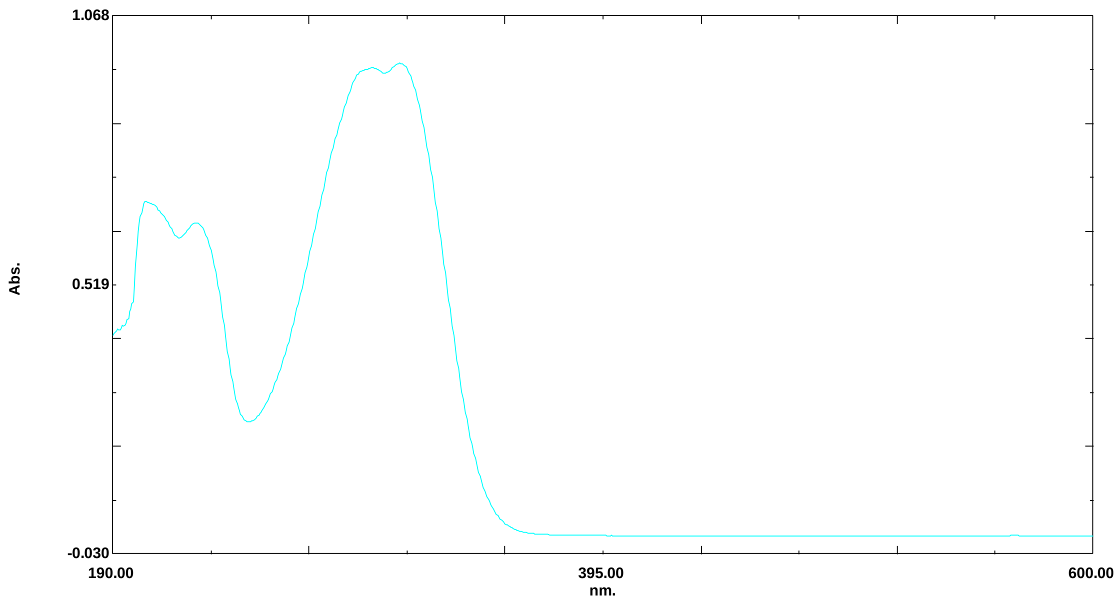

No.	wavelength (nm)	Abs
1	310.00	0.972
2	298.50	0.962
3	224.50	0.646
4	204.00	0.690

Fig. S42 IR spectrum of compound **3**

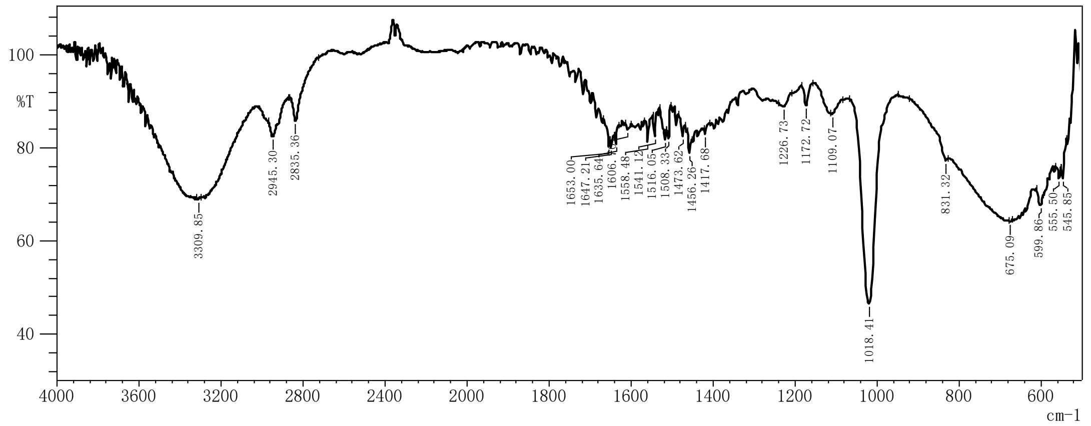

Fig. S43 HRESIMS spectrum of compound **4**

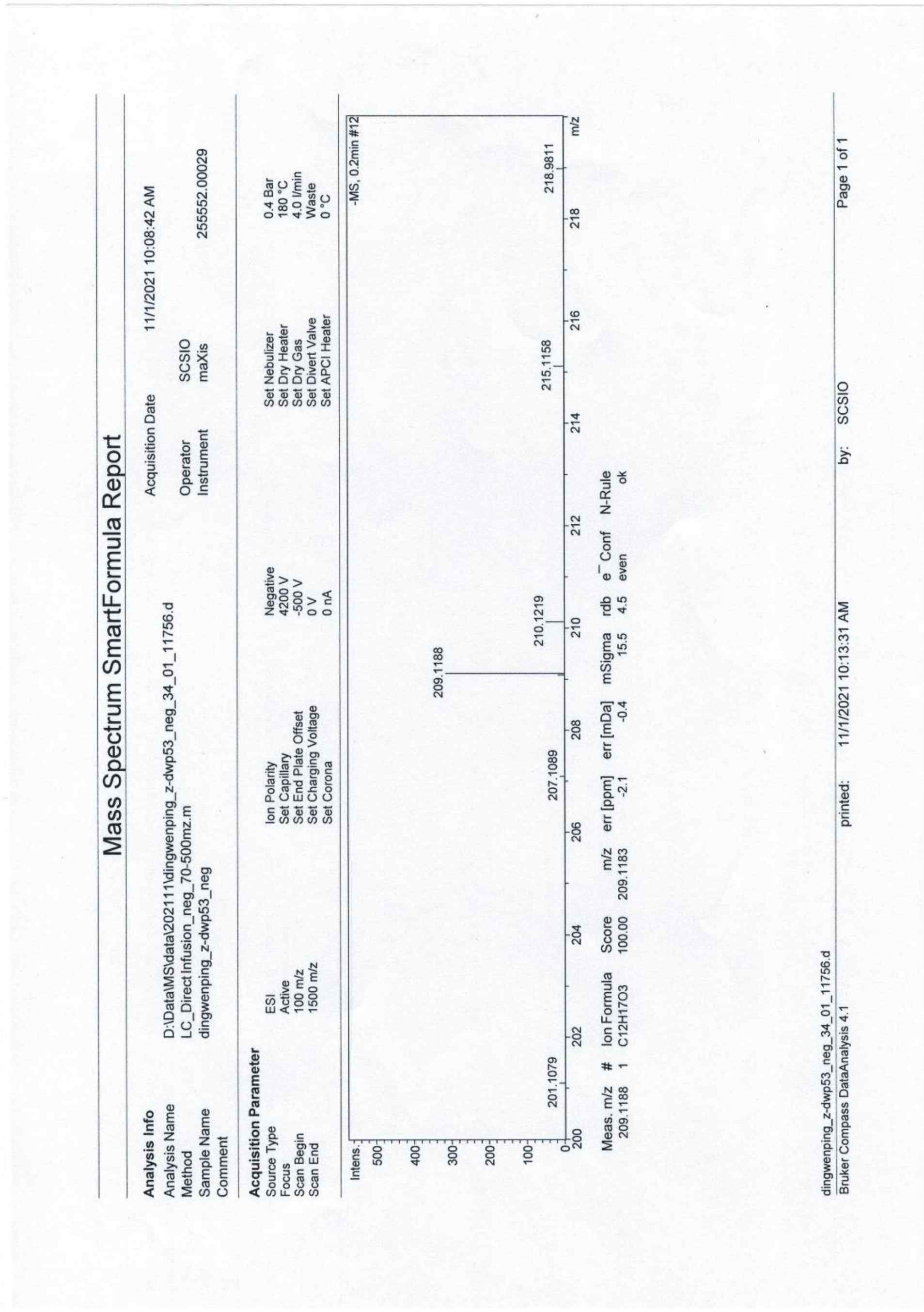

Mass Spectrum SmartFormula Report

Analysis Info

Analysis Name D:\Data\MS\data\202111\dingwenping_z-dwp53_neg_34_01_11756.d
Method LC_Direct Infusion_neg_70-500mz.m
Sample Name dingwenping_z-dwp53_neg
Comment

Acquisition Date 11/1/2021 10:08:42 AM
Operator SCSIO
Instrument maXis 255552.00029

Acquisition Parameter

Source Type	ESI	Ion Polarity	Negative	Set Nebulizer	0.4 Bar
Focus	Active	Set Capillary	4200 V	Set Dry Heater	180 °C
Scan Begin	100 m/z	Set End Plate Offset	-500 V	Set Dry Gas	4.0 l/min
Scan End	1500 m/z	Set Charging Voltage	0 V	Set Divert Valve	Waste
		Set Corona	0 nA	Set APCI Heater	0 °C

Meas. m/z	#	Ion Formula	Score	m/z	err [ppm]	err [mDa]	mSigma	rdb	e⁻ Conf	N-Rule
209.1188	1	C12H17O3	100.00	209.1183	-2.1	-0.4	15.5	4.5	even	ok

dingwenping_z-dwp53_neg_34_01_11756.d
Bruker Compass DataAnalysis 4.1 printed: 11/1/2021 10:13:31 AM by: SCSIO Page 1 of 1

Fig. S44 ^{1}H NMR spectrum (CD_3Cl, 700 MHz) of compound **4**

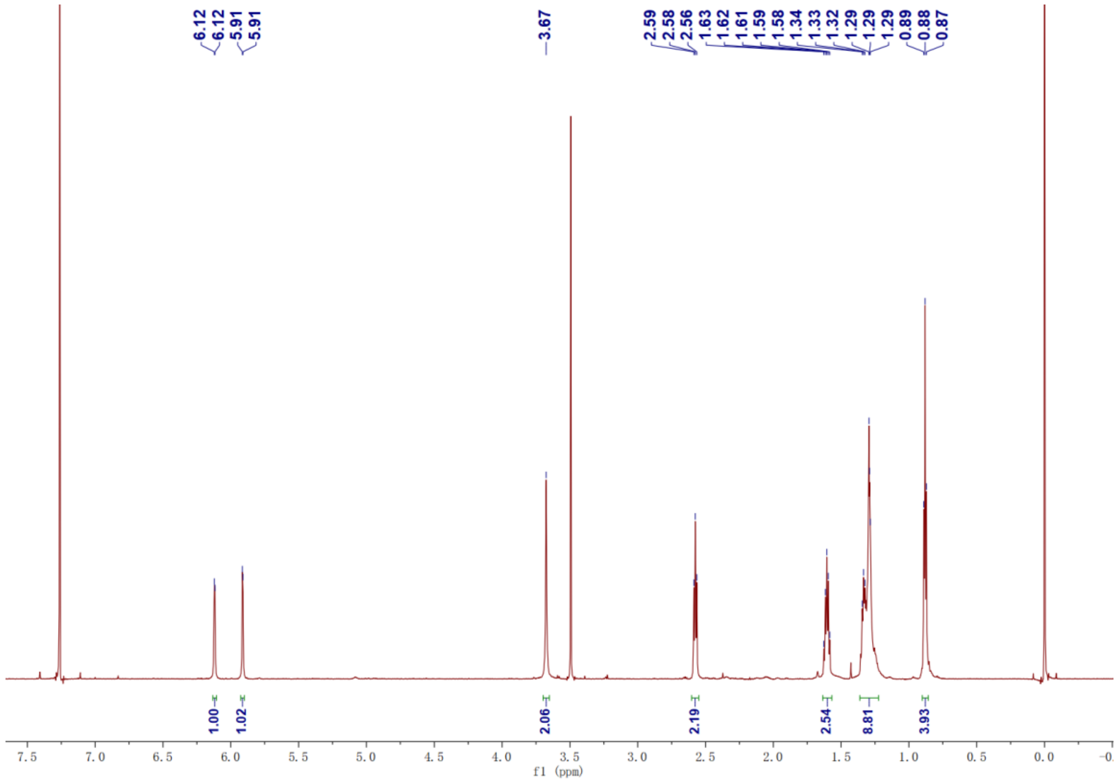

Fig. S45 ^{13}C NMR spectrum (CD_3Cl, 176 MHz) of compound **4**

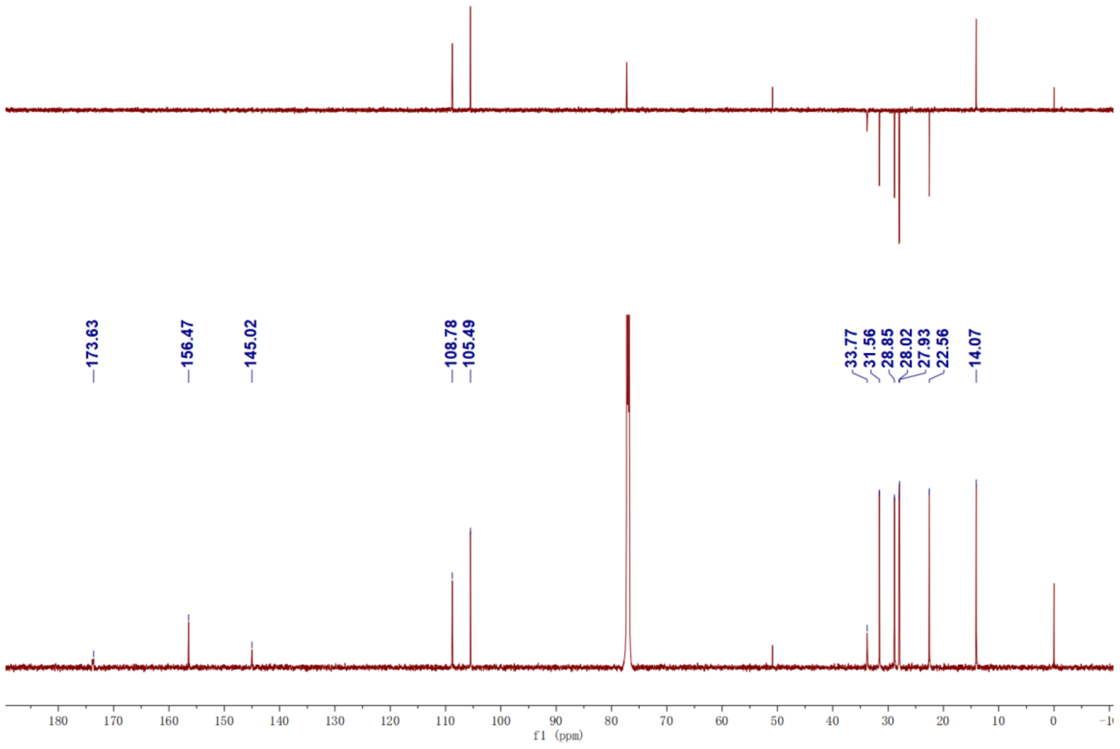

Fig. S46 HSQC spectrum of compound **4**

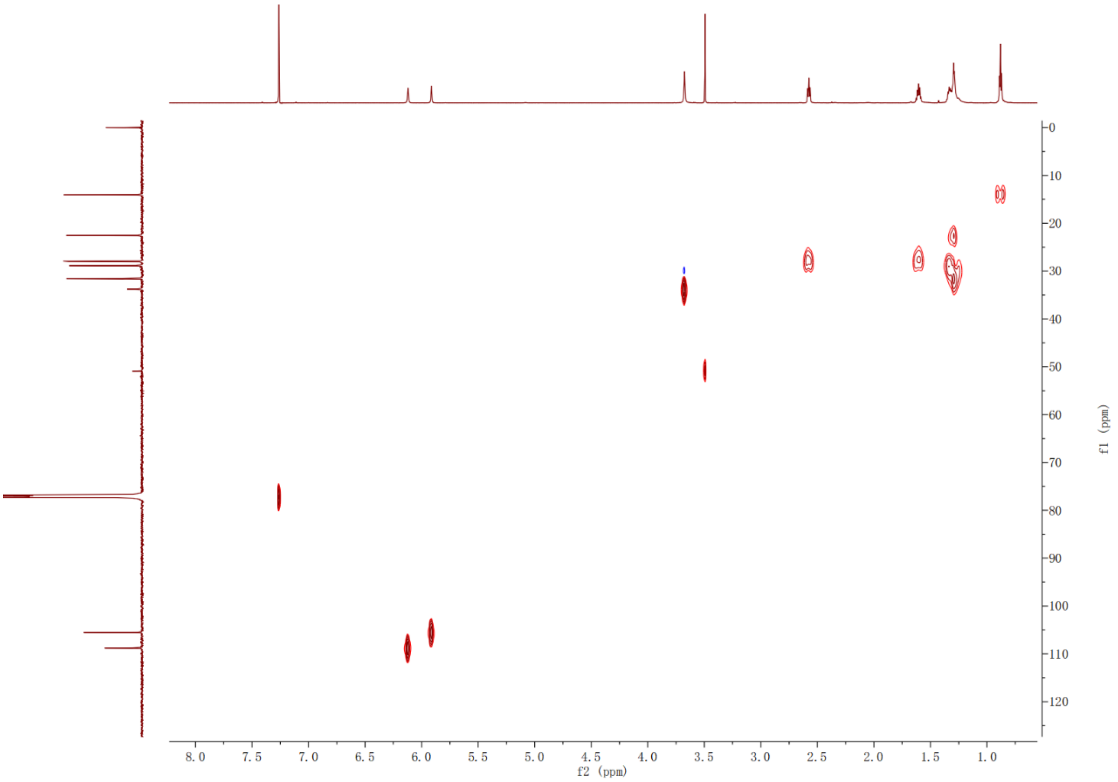

Fig. S47 HMBC spectrum of compound **4**

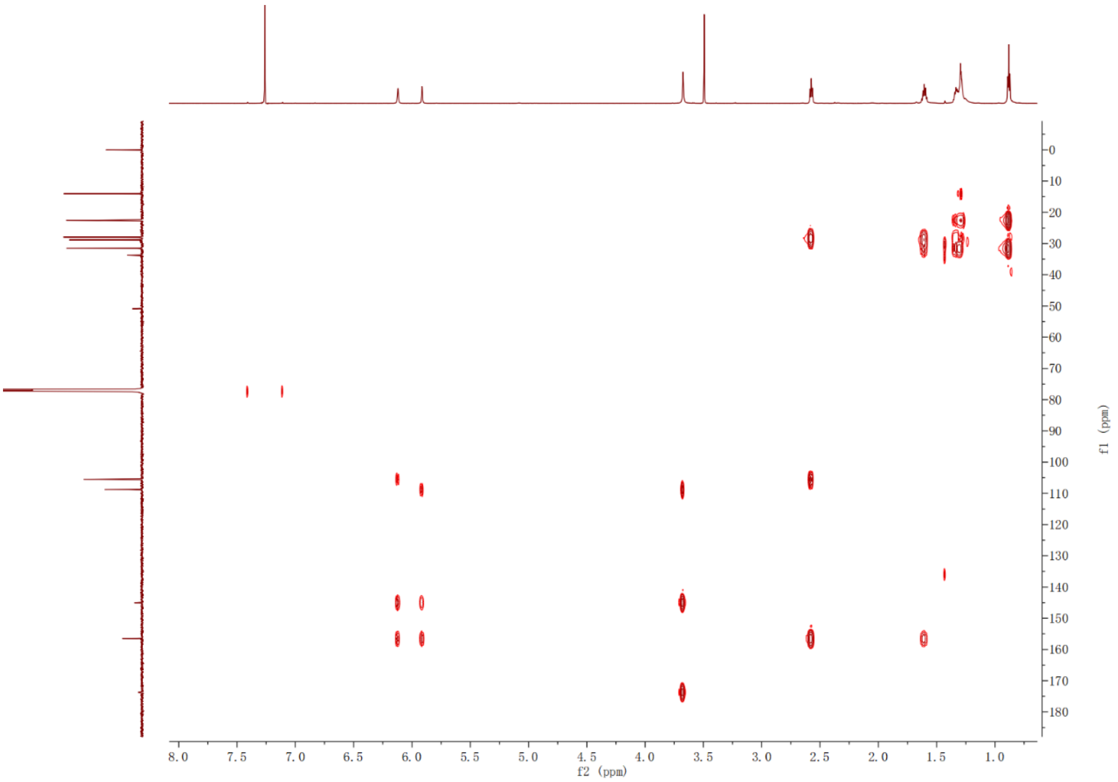

Fig. S48 ^{1}H–^{1}H COSY spectrum of compound **4**

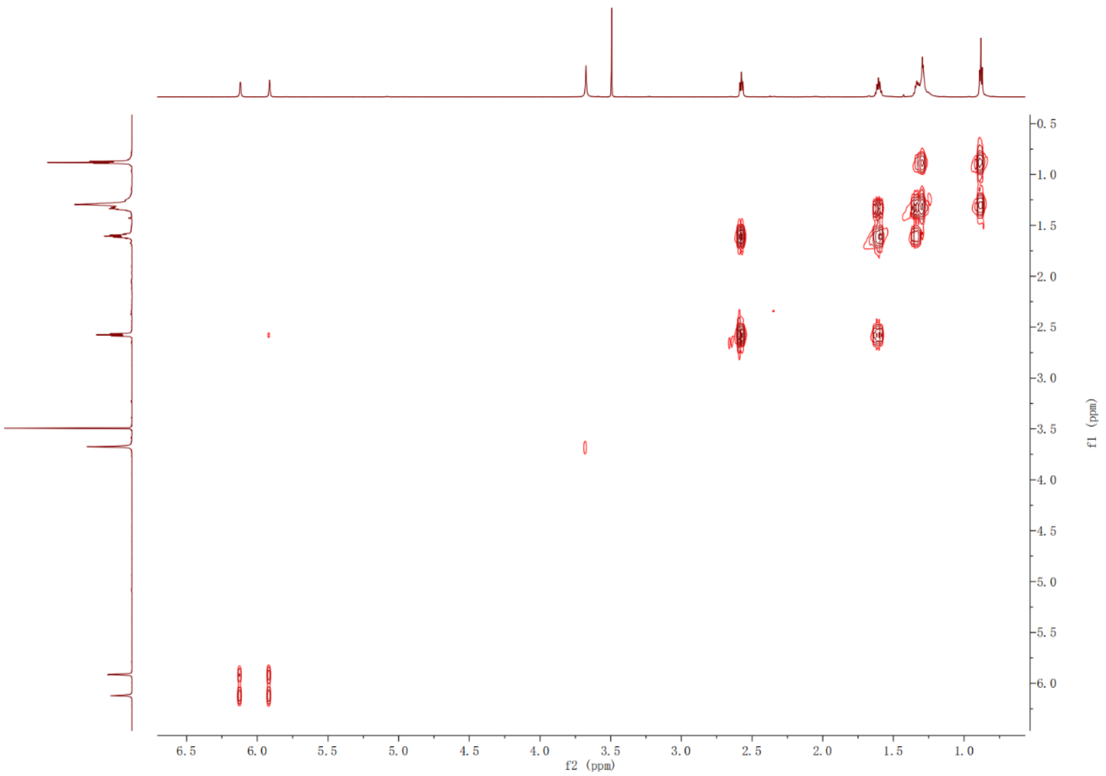

Fig. S49 UV spectrum of compound **4**

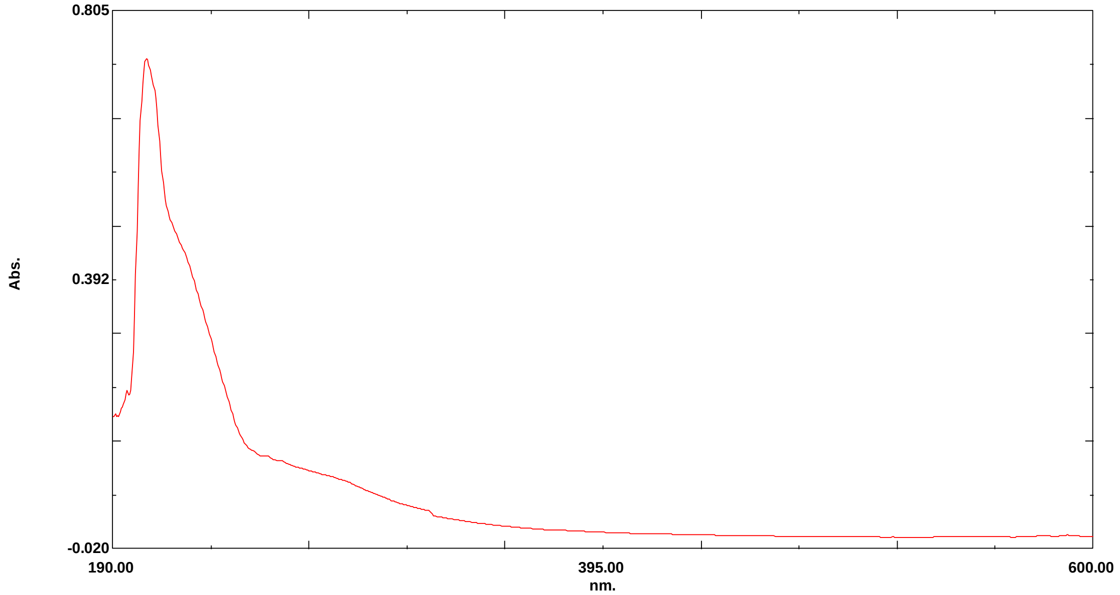

No.	wavelength (nm)	Abs
1	204.00	0.731

Fig. S50 IR spectrum of compound **4**

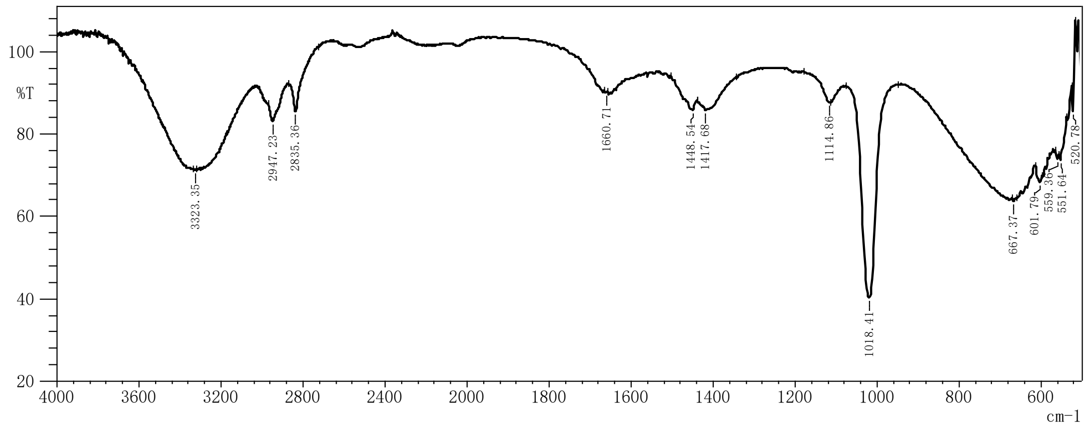

Fig. S51 HRESIMS spectrum of compound **5**

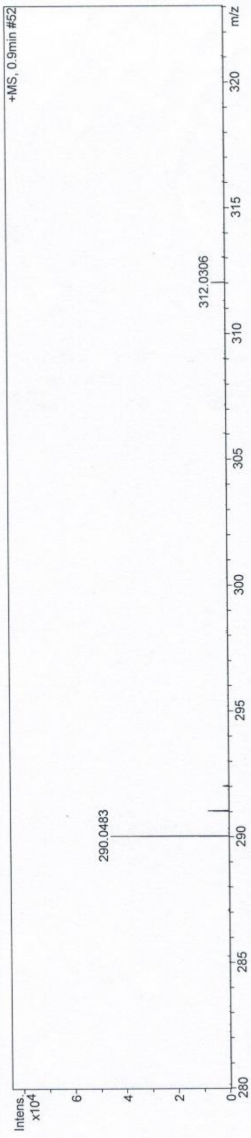

Mass Spectrum SmartFormula Report

Analysis Info

Analysis Name D:\Data\MS\data\202106\dingwenping_Z-DWP50_pos_21_01_10902.d
Method LC_Direct Infusion_pos_70-500mz.m
Sample Name dingwenping_Z-DWP50_pos
Comment

Acquisition Date 6/11/2021 10:47:13 AM
Operator SCSIO
Instrument maXis 255552.00029

Acquisition Parameter

Source Type	ESI	Ion Polarity	Positive	Set Nebulizer	0.4 Bar
Focus	Active	Set Capillary	4500 V	Set Dry Heater	180 °C
Scan Begin	70 m/z	Set End Plate Offset	-500 V	Set Dry Gas	4.0 l/min
Scan End	1500 m/z	Set Charging Voltage	0 V	Set Divert Valve	Waste
		Set Corona	0 nA	Set APCI Heater	0 °C

Meas. m/z	#	Ion Formula	Score	m/z	err [ppm]	err [mDa]	mSigma	rdb	e⁻ Conf	N-Rule
290.0483	1	C14H12NO4S	100.00	290.0482	0.6	0.2	10.3	9.5	even	ok
312.0306	1	C14H11NNaO4S	100.00	312.0301	1.6	0.5	14.2	9.5	even	ok

dingwenping_Z-DWP50_pos_21_01_10902.d printed: 6/11/2021 10:51:41 AM by: SCSIO
Bruker Compass DataAnalysis 4.1 Page 1 of 1

Fig. S52 ^{1}H NMR spectrum (CD_3OD, 700 MHz) of compound **5**

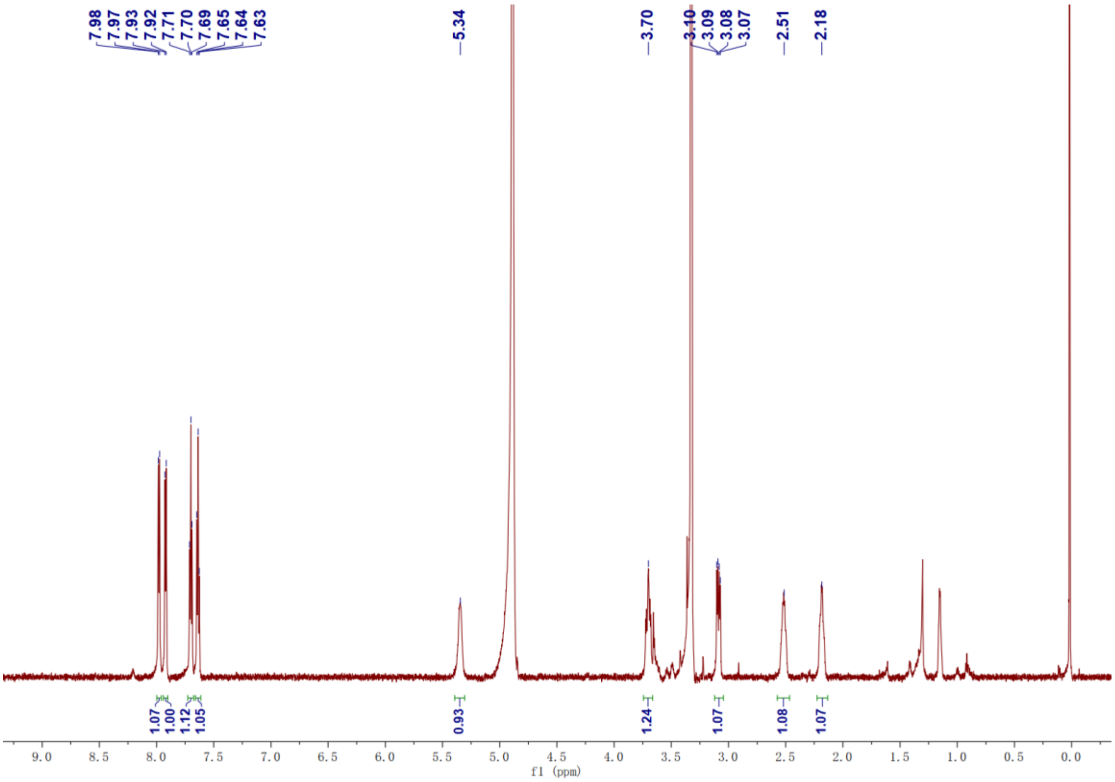

Fig. S53 ^{13}C NMR spectrum (CD_3OD, 176 MHz) of compound **5**

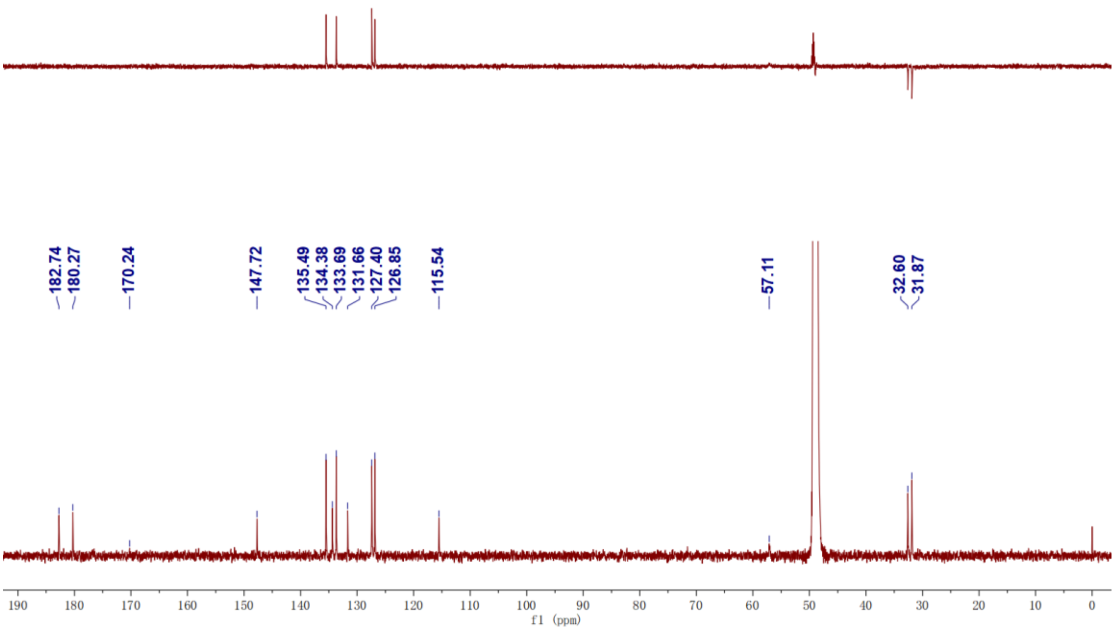

Fig. S54 HSQC spectrum of compound **5**

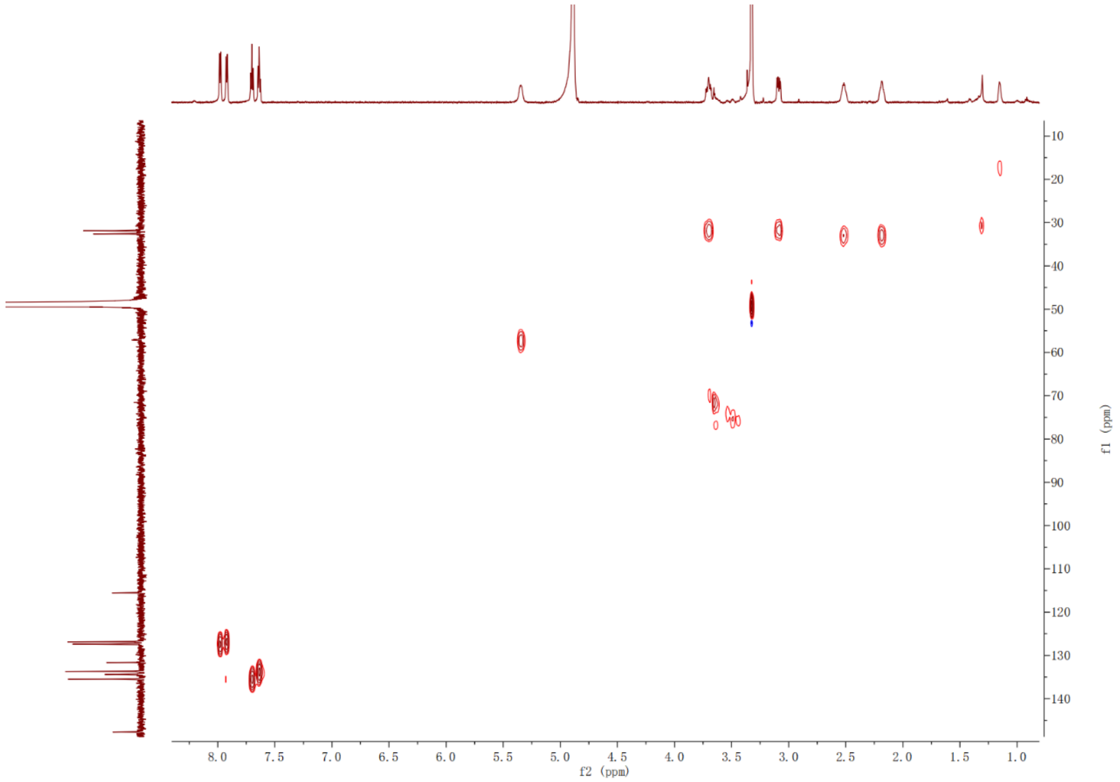

Fig. S55 HMBC spectrum of compound **5**

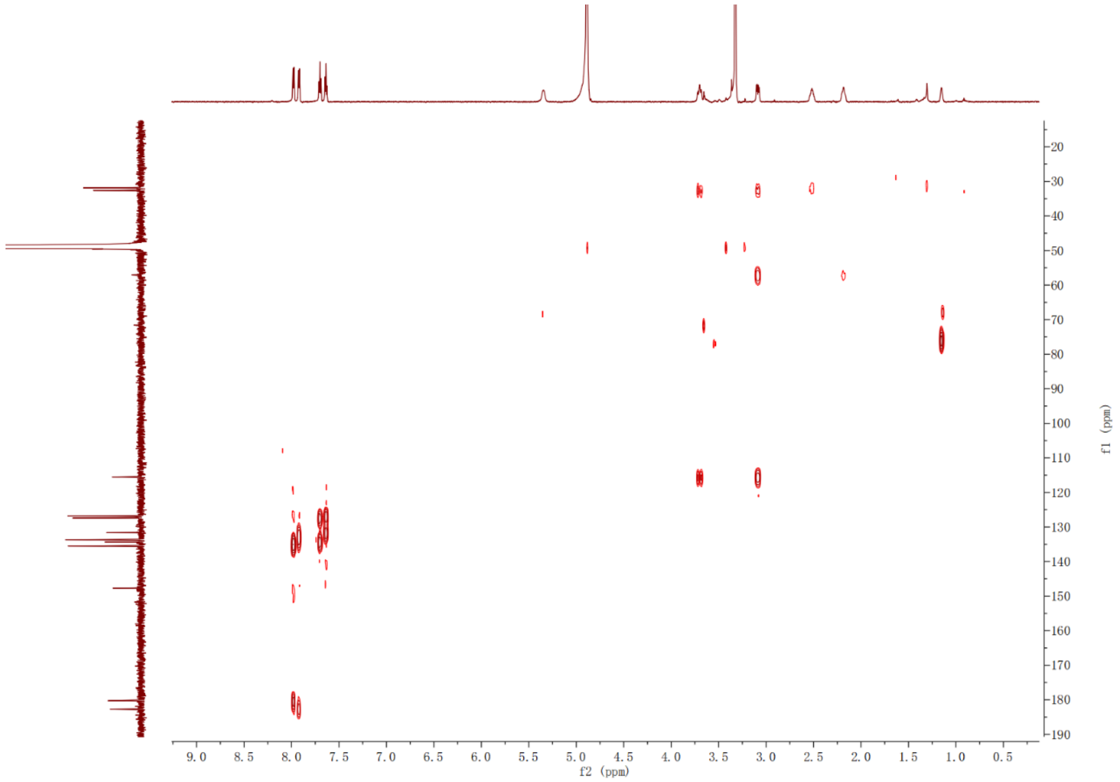

Fig. S56 ^{1}H–^{1}H COSY spectrum of compound **5**

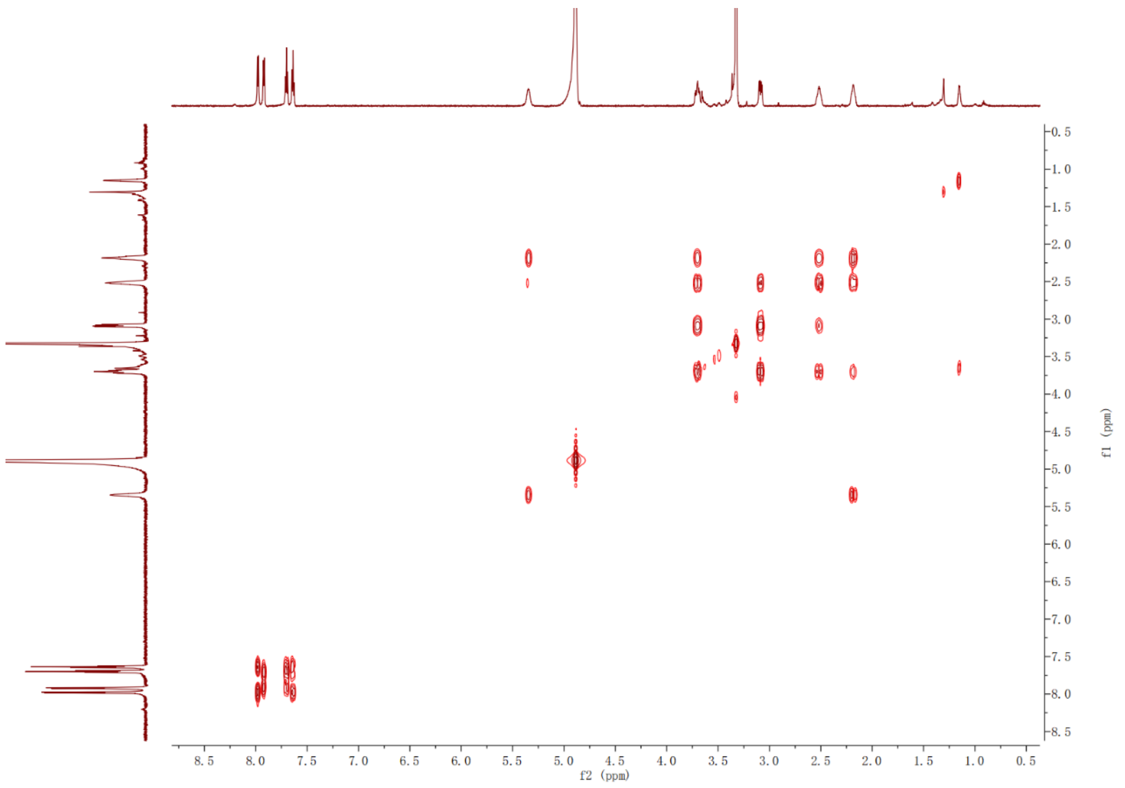

Fig. S57 ESI-MS spectrum of compound **6**

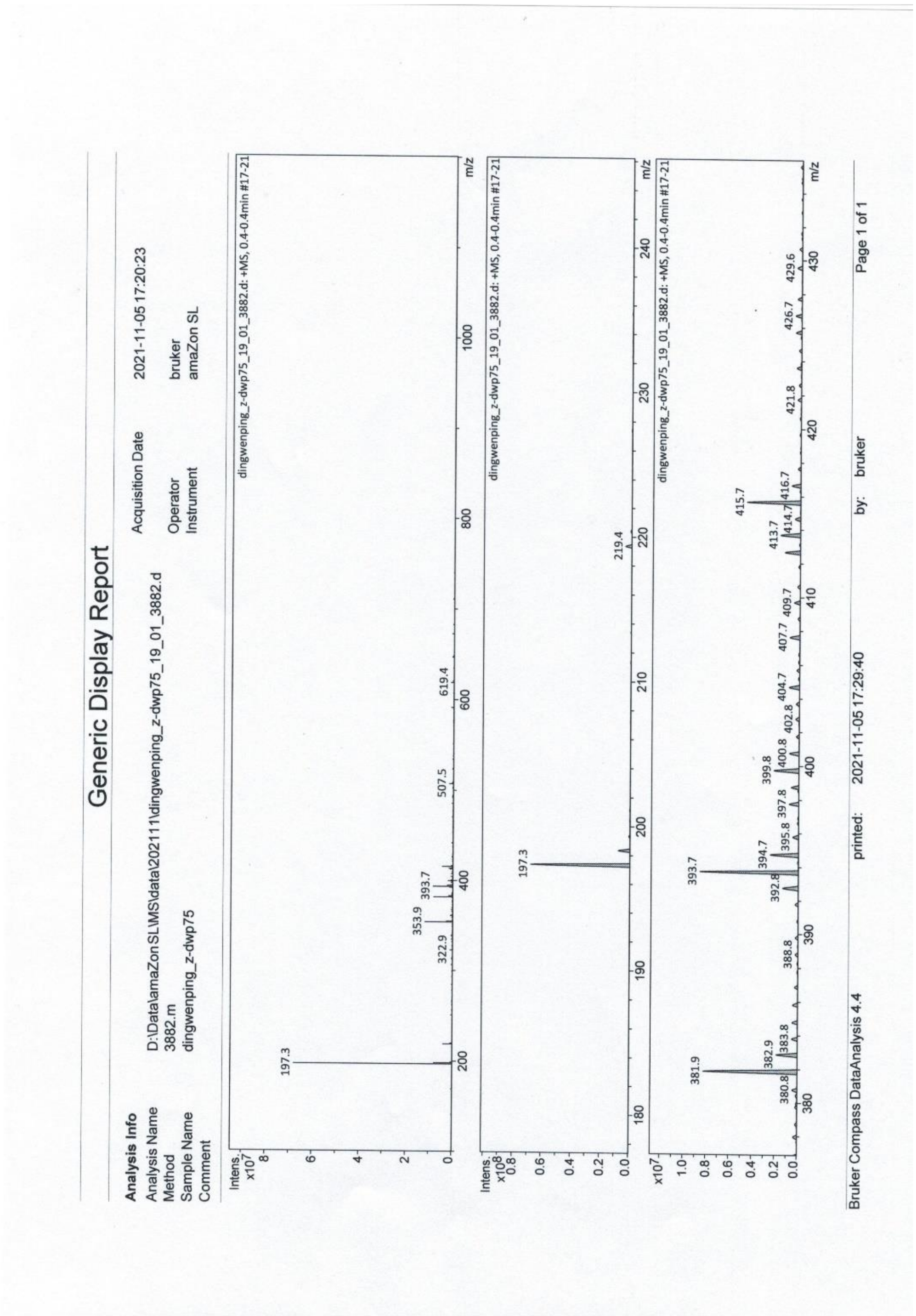

Fig. S58 ^{1}H NMR spectrum (CD_3OD, 500 MHz) of compound **6**

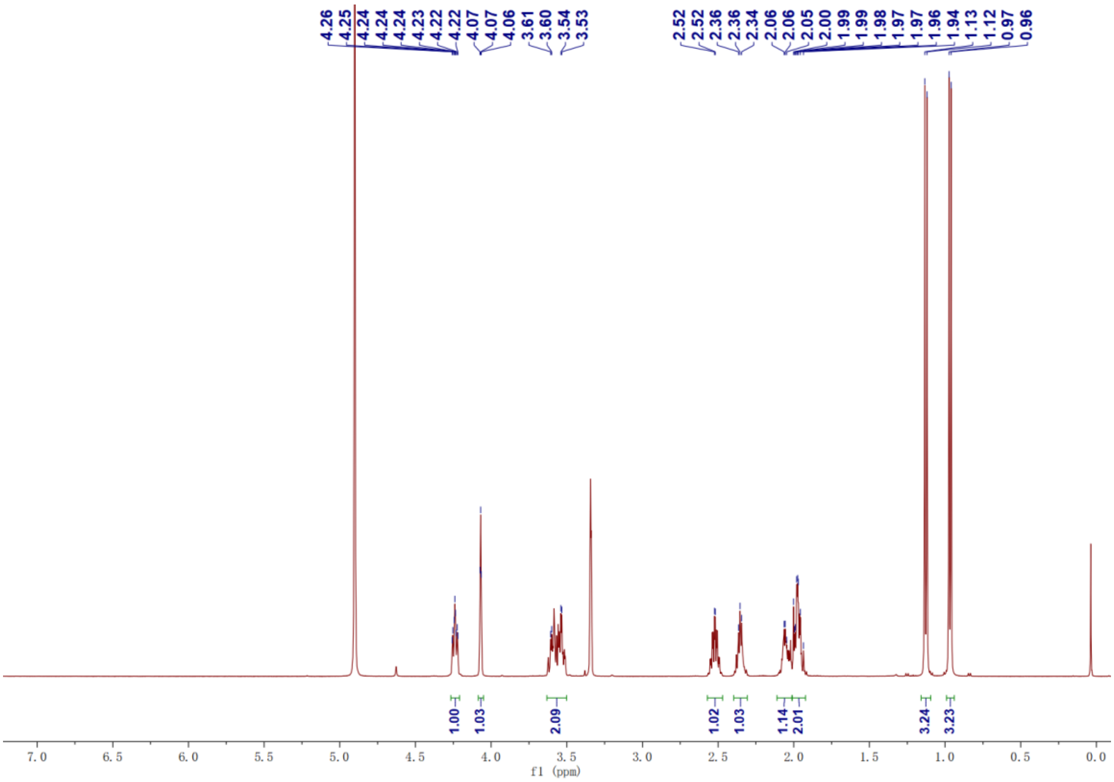

Fig. S59 ^{13}C NMR spectrum (CD_3OD, 126 MHz) of compound **6**

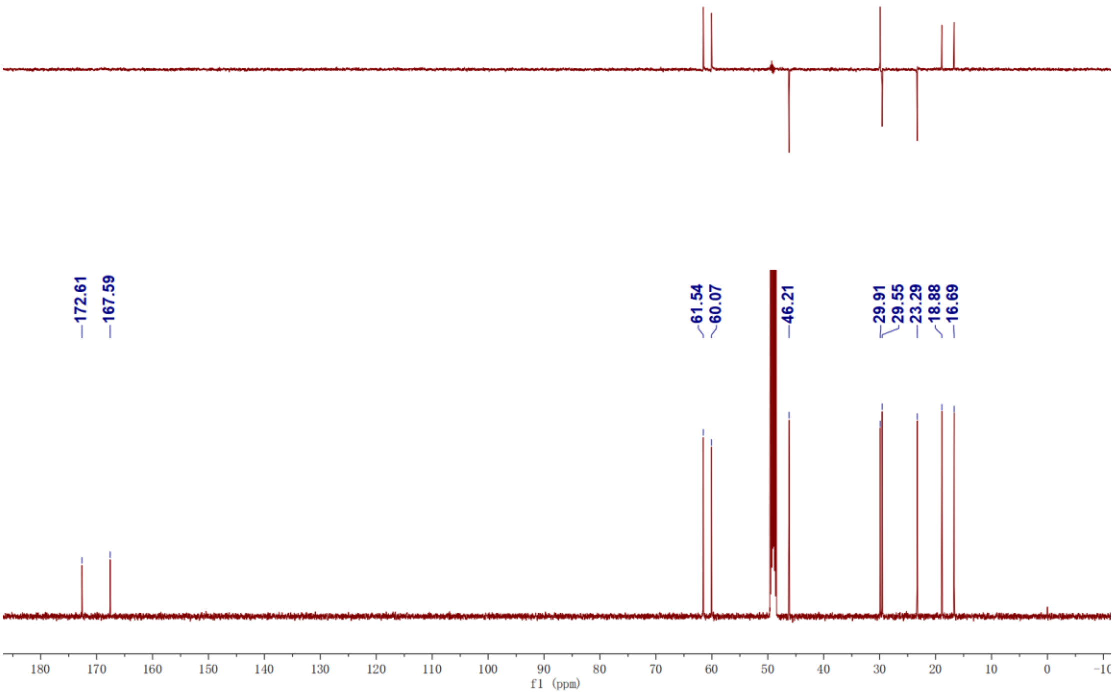

Crystallographic data of compound **6**

Crystal Data for $C_{10}H_{16}N_2O_2$ (M =1569.98 g/mol): orthorhombic, space group $P2_12_12_1$ (no. 19), a = 5.61820(10) Å, b = 10.25990(10) Å, c = 34.2074(4) Å, V = 1971.79(5) Å^3, Z = 8, T = 99.99(10) K, μ(Cu Kα) = 0.756 mm^{-1}, $Dcalc$ = 1.322 g/cm^3, 19431 reflections measured (5.166° ≤ 2Θ ≤ 148.834°), 3939 unique (R_{int} = 0.0292, R_{sigma} = 0.0222) which were used in all calculations. The final R_1 was 0.0283 (I > 2σ(I)) and wR_2 was 0.0720 (all data).

Fig. S60 ESI-MS spectrum of compound **7**

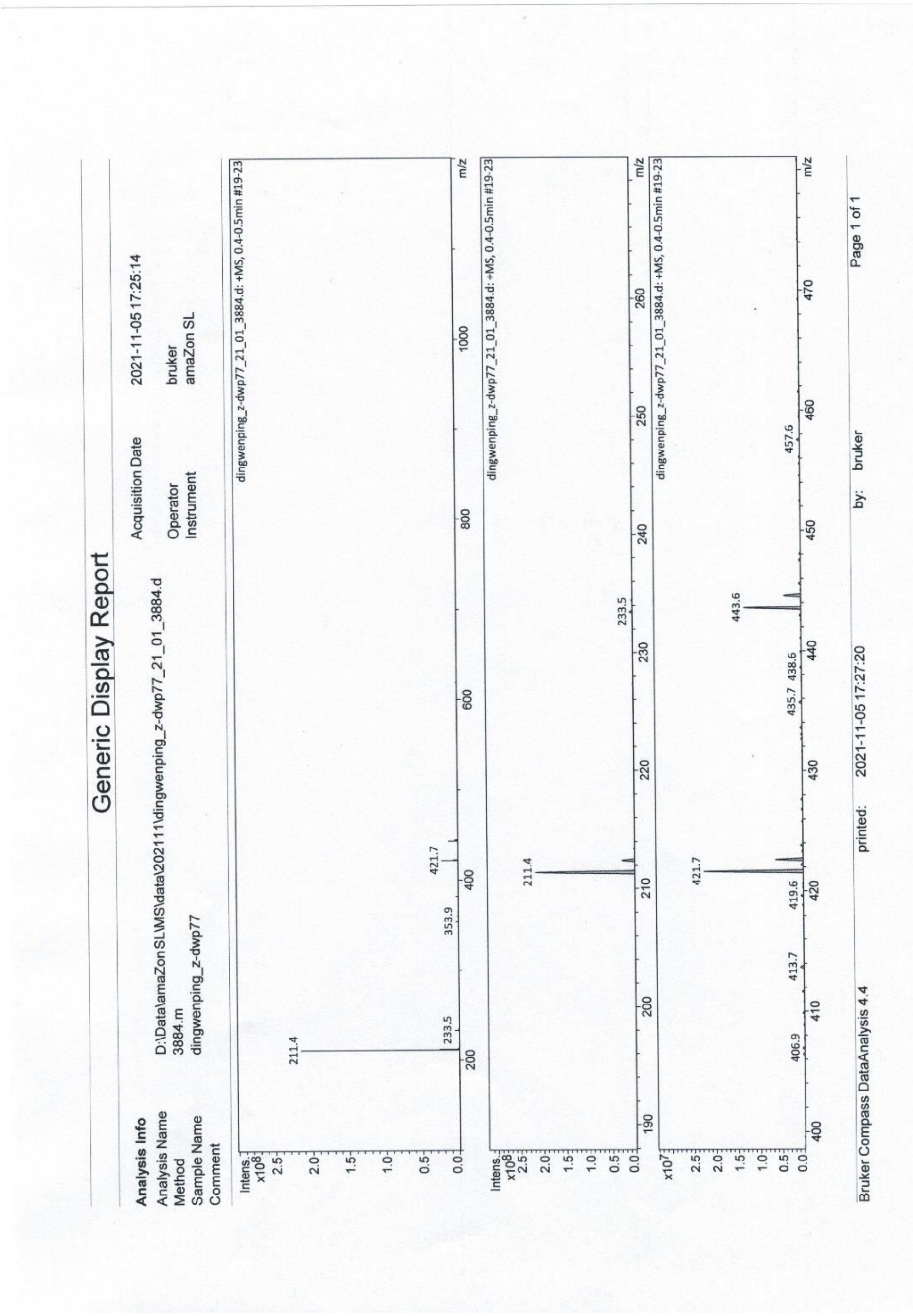

Fig. S61 ^{1}H NMR spectrum (CD_3OD, 500 MHz) of compound **7**

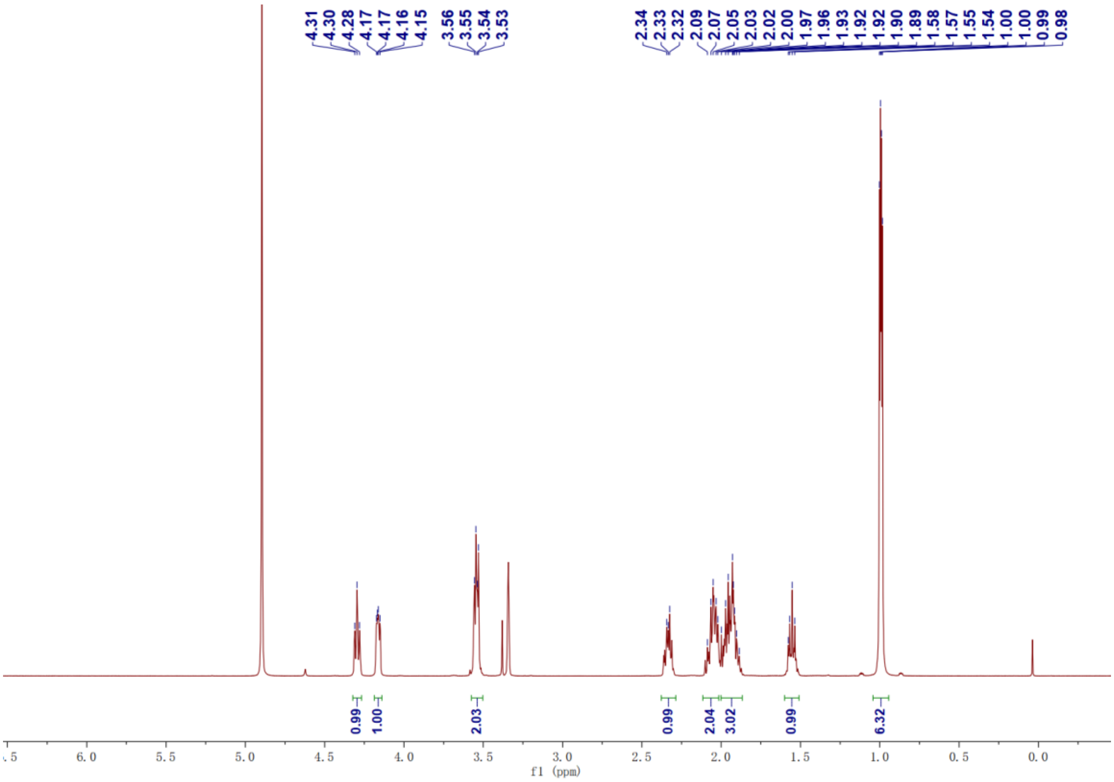

Fig. S62 ^{13}C NMR spectrum (CD_3OD, 126 MHz) of compound **7**

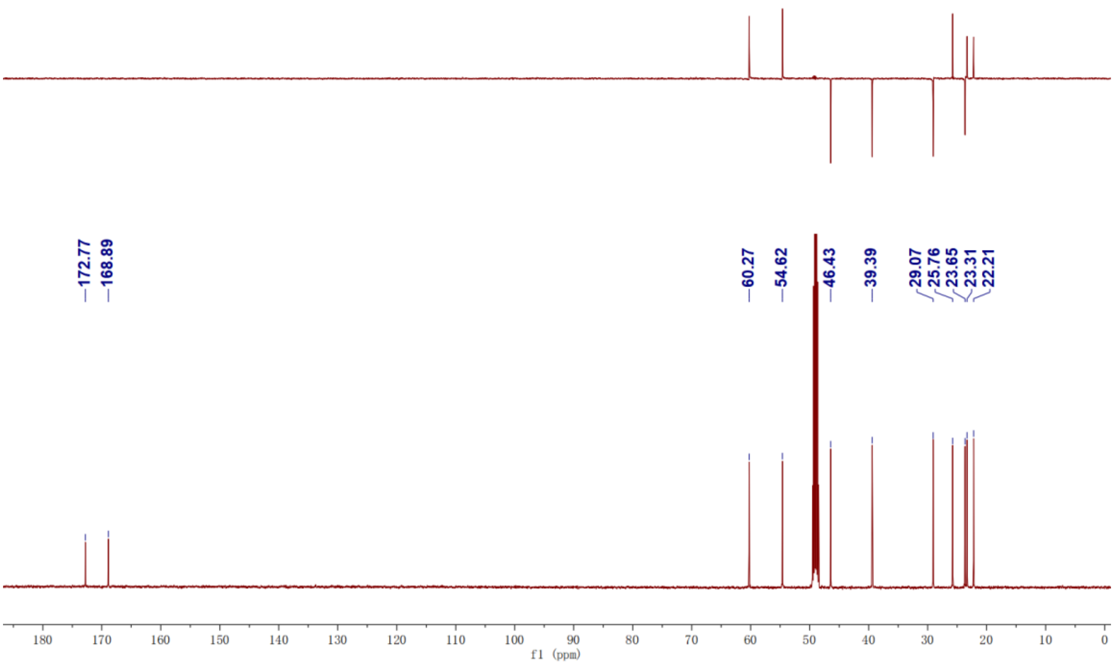

Crystallographic data of compound **7**

Crystal Data for $C_{11}H_{18}N_2O_2$ (M =210.27 g/mol): orthorhombic, space group $P2_12_12_1$ (no. 19), a = 6.3034(2) Å, b = 9.2289(3) Å, c = 19.5991(7) Å, V = 1140.15(7) $Å^3$, Z = 4, T = 100.00(10) K, μ(Cu Kα) = 0.686 mm^{-1}, $Dcalc$ = 1.225 g/cm^3, 10779 reflections measured (9.024° ≤ 2Θ ≤ 147.842°), 2274 unique (R_{int} = 0.0609, R_{sigma} = 0.0335) which were used in all calculations. The final R_1 was 0.0547 (I > 2σ(I)) and wR_2 was 0.1469 (all data).

Fig. S63 ESI-MS spectrum of compound **8**

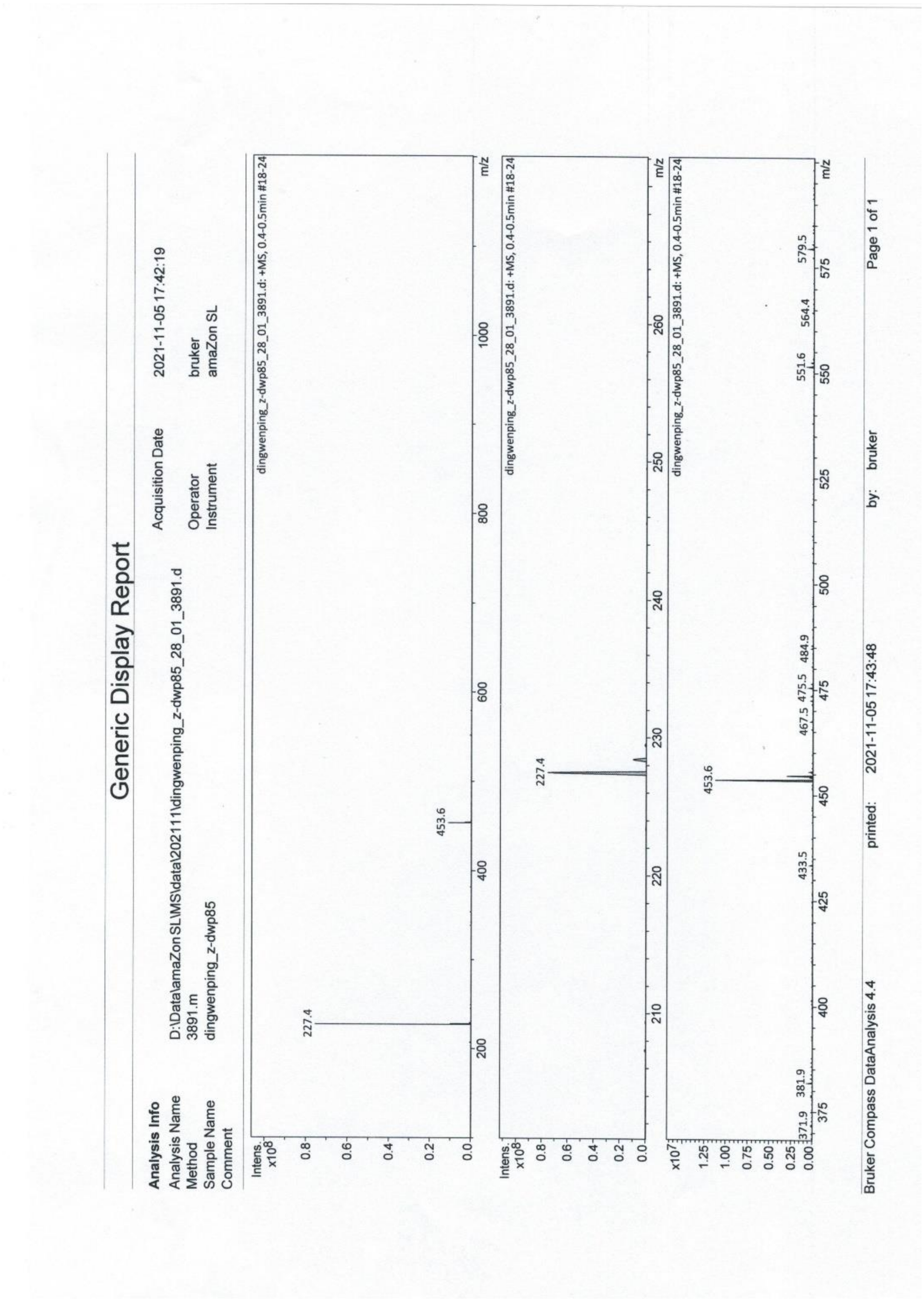

Fig. S64 ^{1}H NMR spectrum (CD_3OD, 500 MHz) of compound **8**

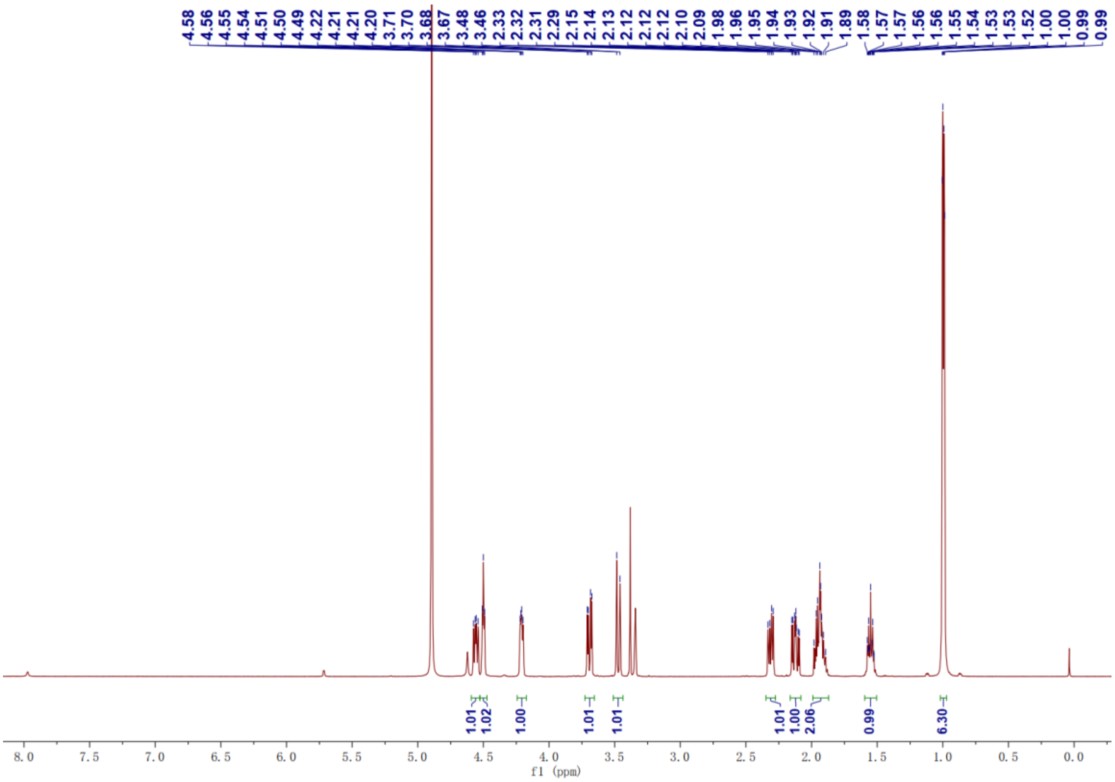

Fig. S65 ^{13}C NMR spectrum (CD_3OD, 126 MHz) of compound **8**

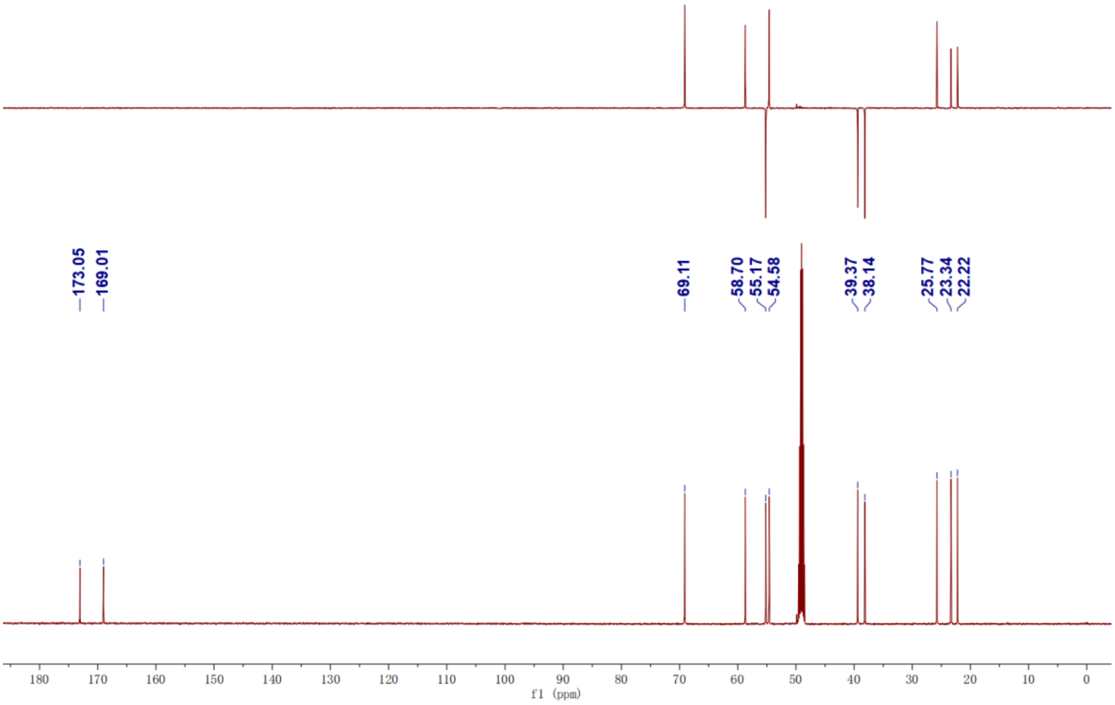

Crystallographic data of compound **8**

Crystal Data for $C_{11}H_{18}N_2O_3$ (*M* =226.27 g/mol): orthorhombic, space group $P2_12_12_1$ (no. 19), *a* = 6.28190(10) Å, *b* = 9.4412(2) Å, *c* = 19.6668(3) Å, *V* = 1166.41(4) Å^3, *Z* = 4, *T* = 100.00(10) K, μ(Cu Kα) = 0.775 mm^{-1}, *Dcalc* = 1.289 g/cm^3, 5357 reflections measured (8.992° ≤ 2Θ ≤ 148.308°), 2286 unique (R_{int} = 0.0248, R_{sigma} = 0.0276) which were used in all calculations. The final R_1 was 0.0296 (I > 2σ(I)) and wR_2 was 0.0781 (all data).

Fig. S66 ESI-MS spectrum of compound **9**

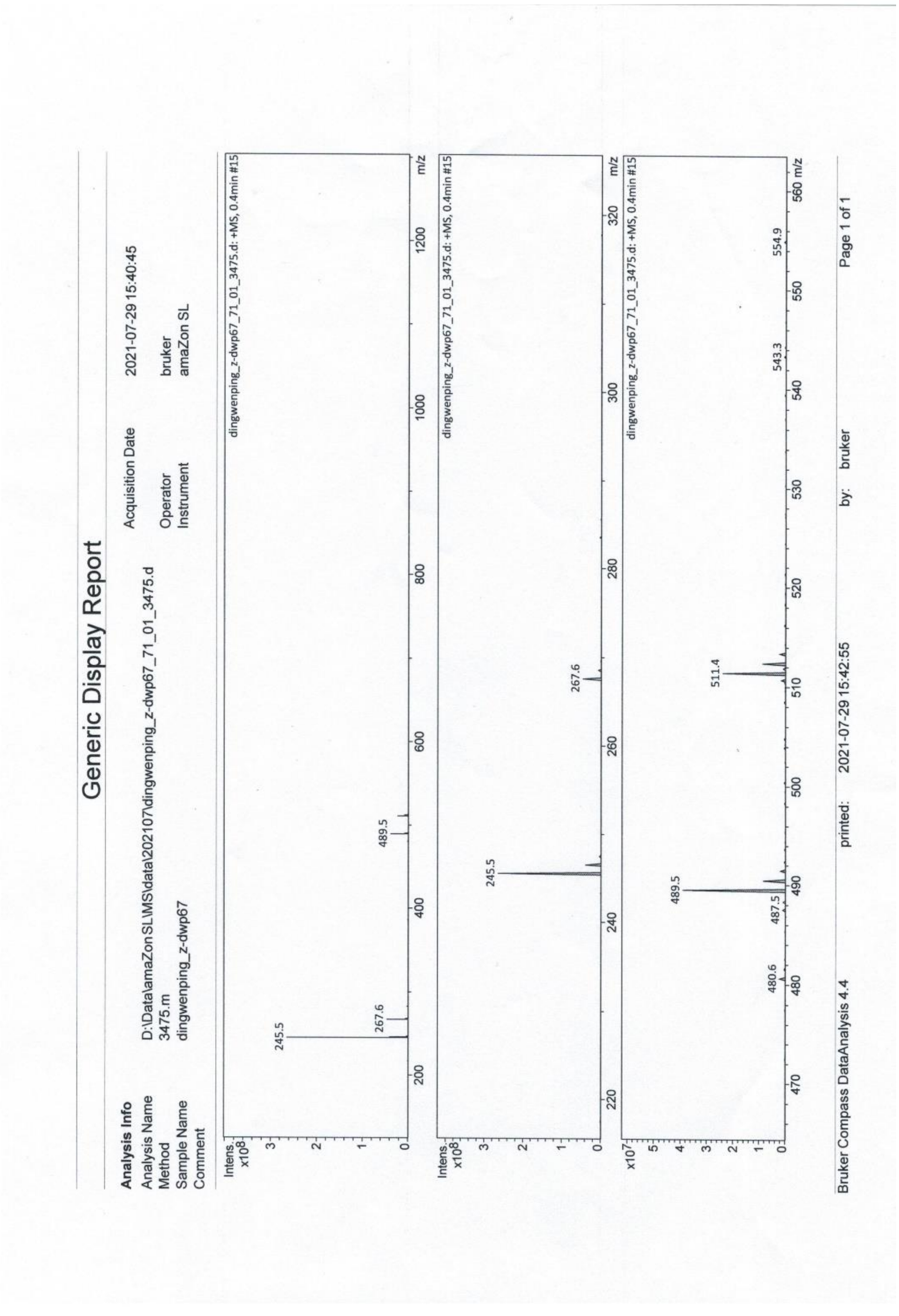

Fig. S67 ^{1}H NMR spectrum (CD_3OD, 500 MHz) of compound **9**

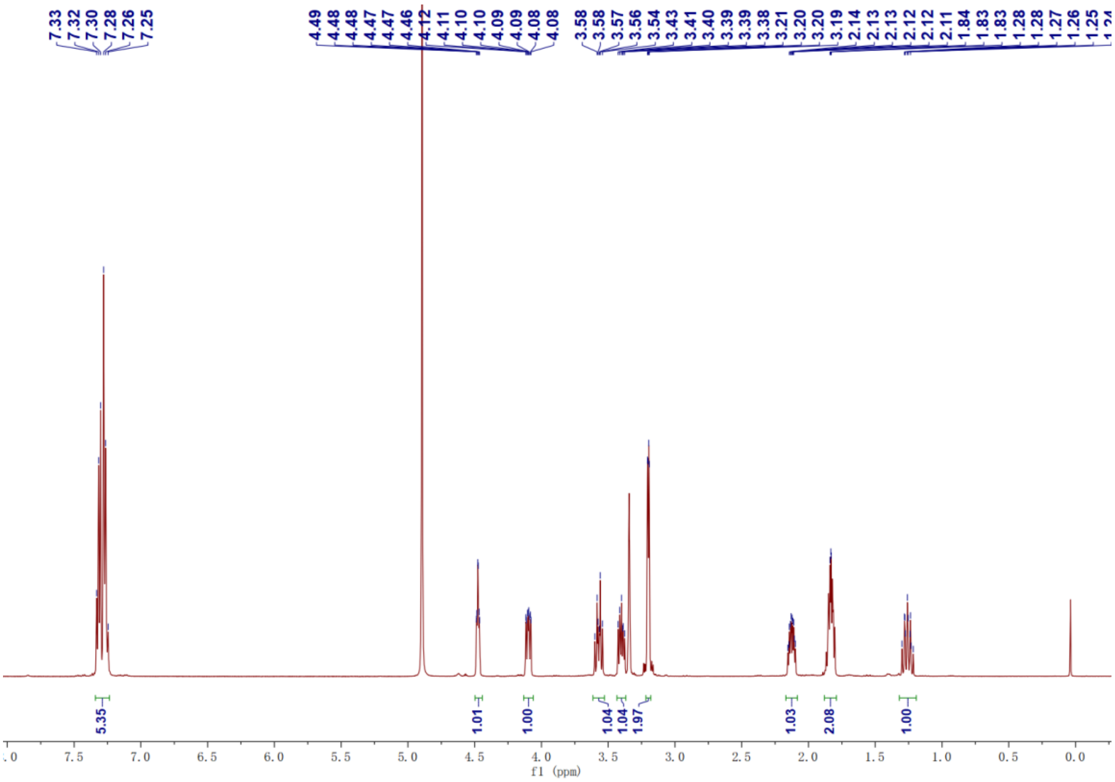

Fig. S68 ^{13}C NMR spectrum (CD_3OD, 126 MHz) of compound **9**

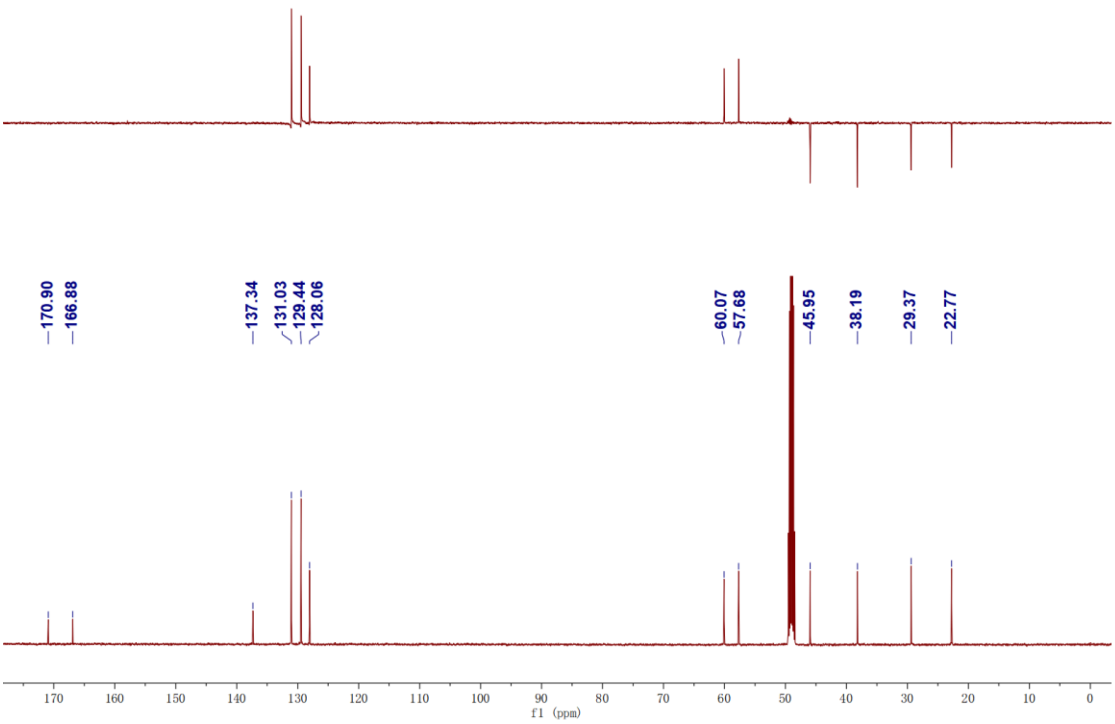

Crystallographic data of compound **9**

Crystal Data for $C_{14}H_{16}N_2O_2$ (M =244.29 g/mol): monoclinic, space group P2$_1$ (no. 4), a = 11.9476(2) Å, b = 8.86980(10) Å, c = 12.3429(2) Å, β = 109.501(2)°, V = 1232.98(3) Å^3, Z = 4, T = 99.99(10) K, μ(Cu Kα) = 0.721 mm^{-1}, $Dcalc$ = 1.316 g/cm^3, 22922 reflections measured (7.598° ≤ 2Θ ≤ 148.674°), 4840 unique (R_{int} = 0.0468, R_{sigma} = 0.0353) which were used in all calculations. The final R_1 was 0.0453 (I > 2σ(I)) and wR_2 was 0.1227 (all data).

Fig. S69 ESI-MS spectrum of compound **10**

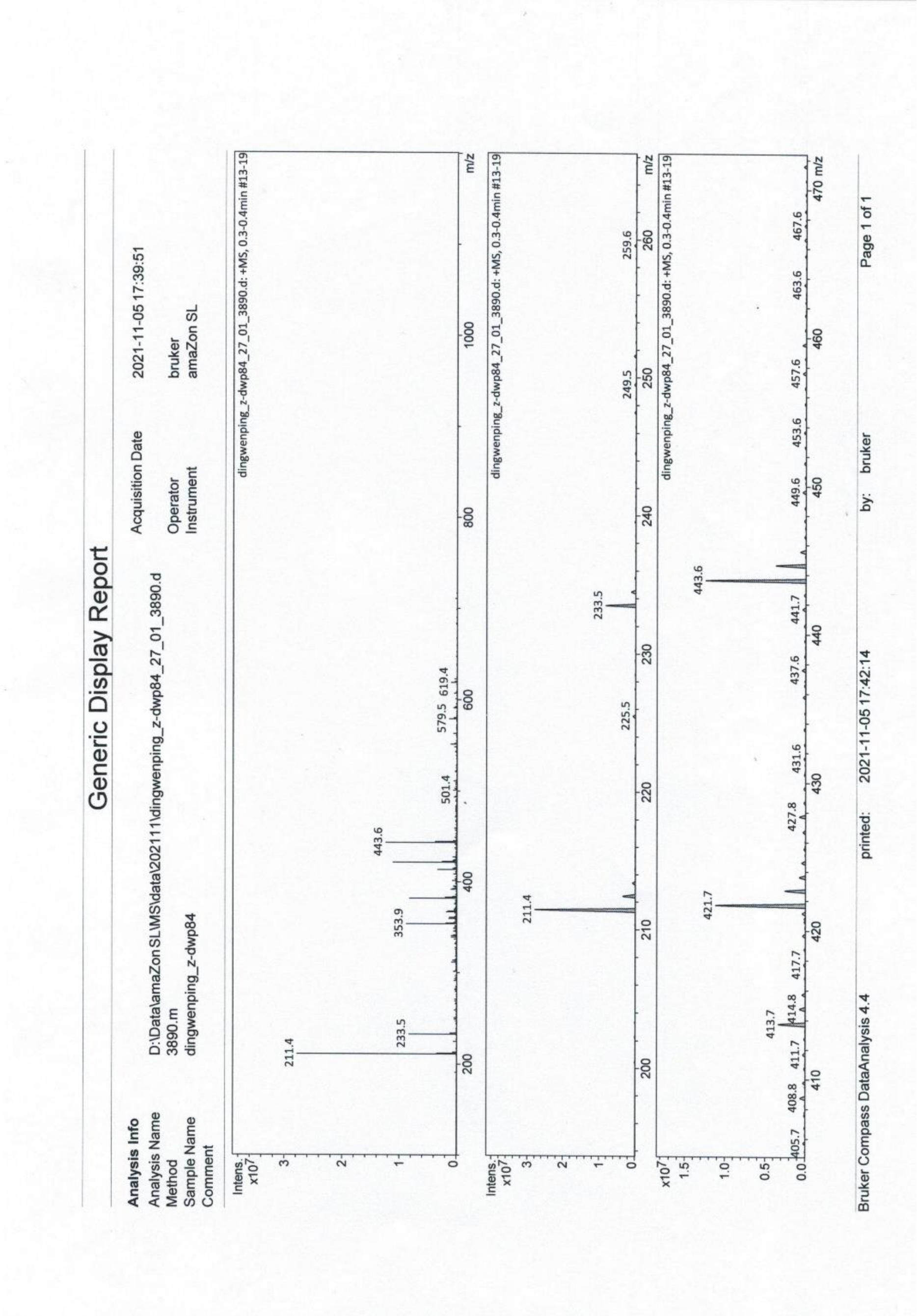

Fig. S70 ^{1}H NMR spectrum (CD_3OD, 700 MHz) of compound **10**

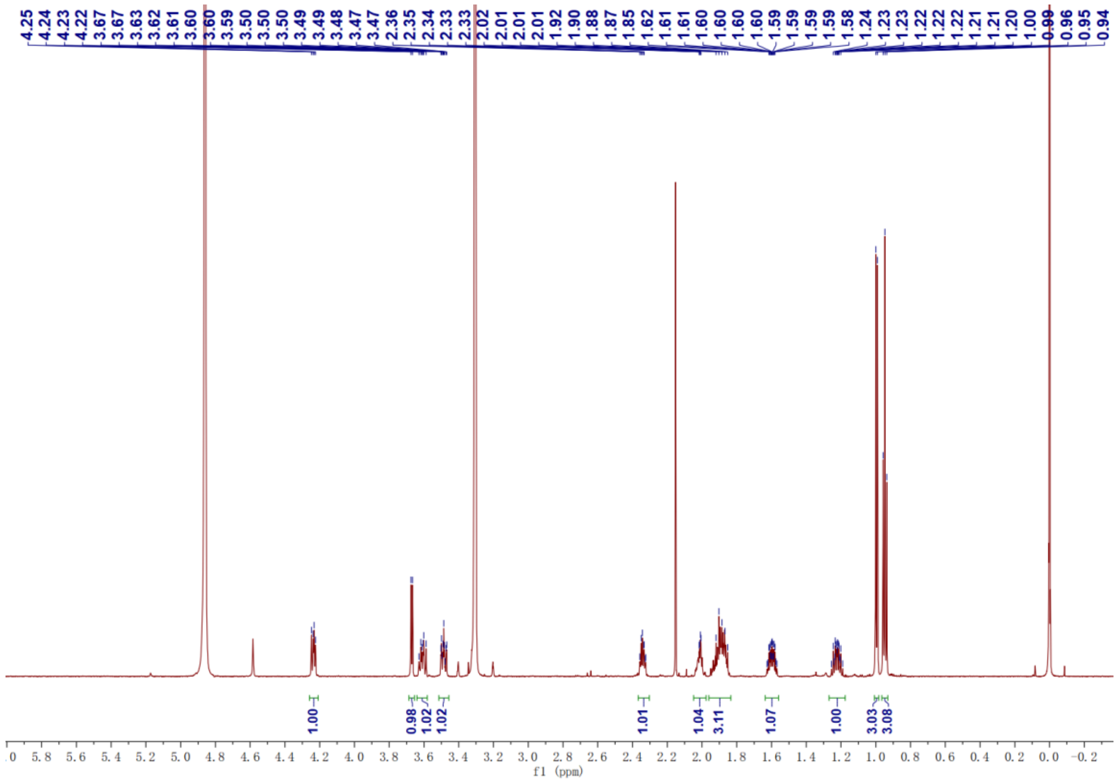

Fig. S71 ^{13}C NMR spectrum (CD_3OD, 176 MHz) of compound **10**

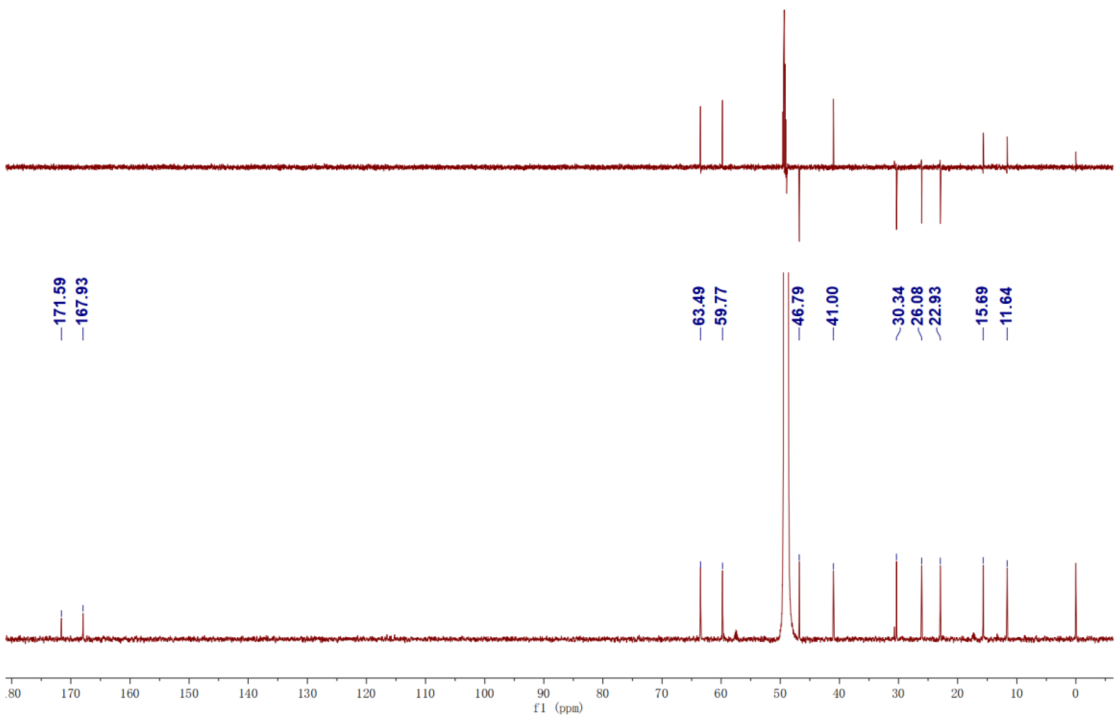

Fig. S72 ESI-MS spectrum of compound **11**

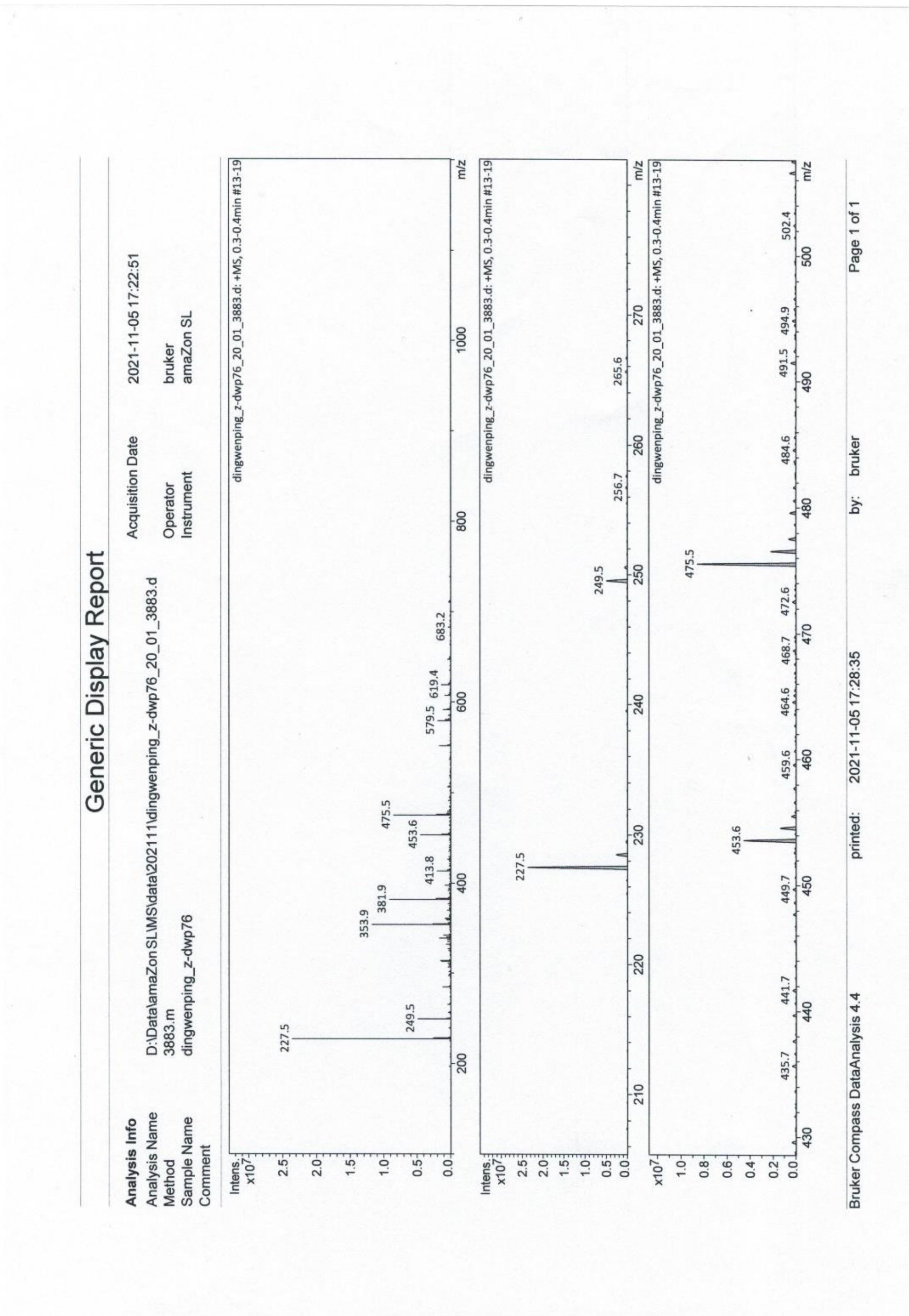

Fig. S73 ¹H NMR spectrum (CD_3OD, 700 MHz) of compound **11**

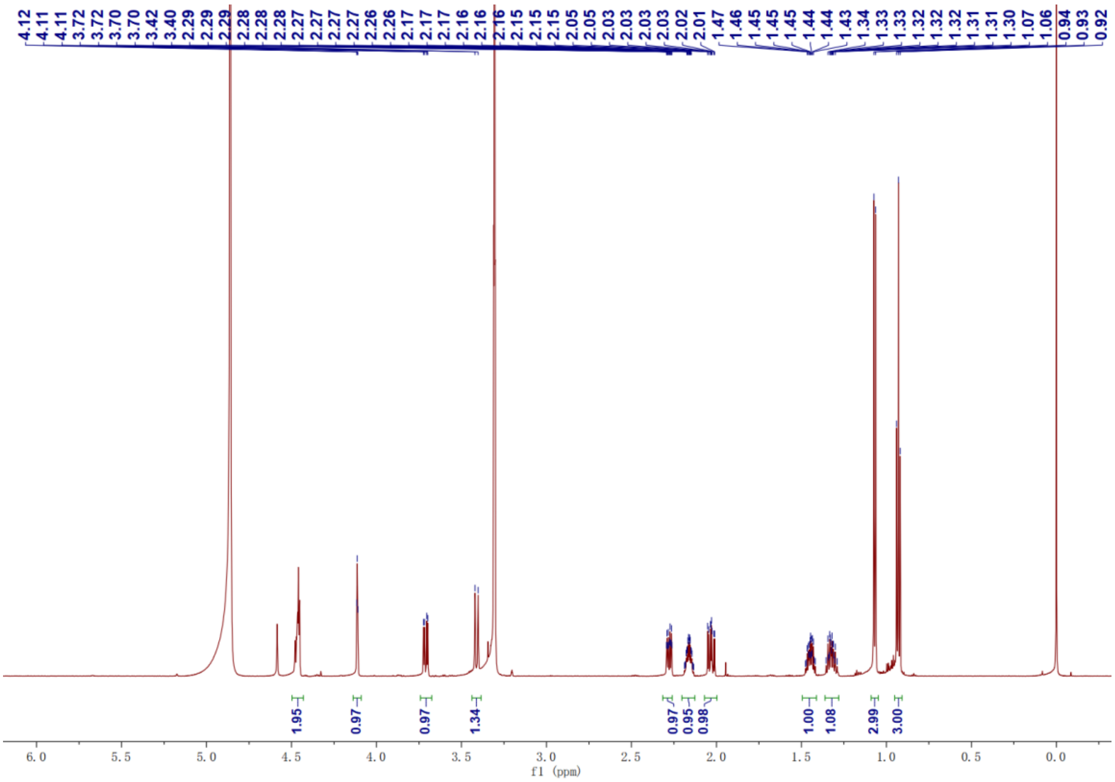

Fig. S74 ¹³C NMR spectrum (CD_3OD, 176 MHz) of compound **11**

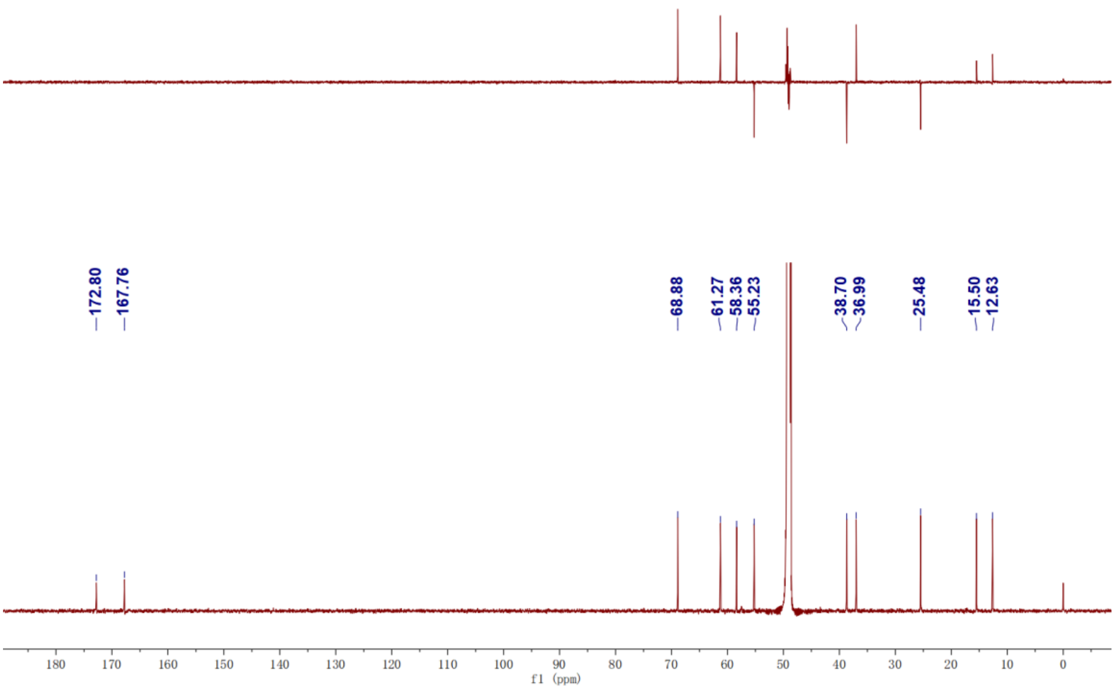

Fig. S75 ESI-MS spectrum of compound **12**

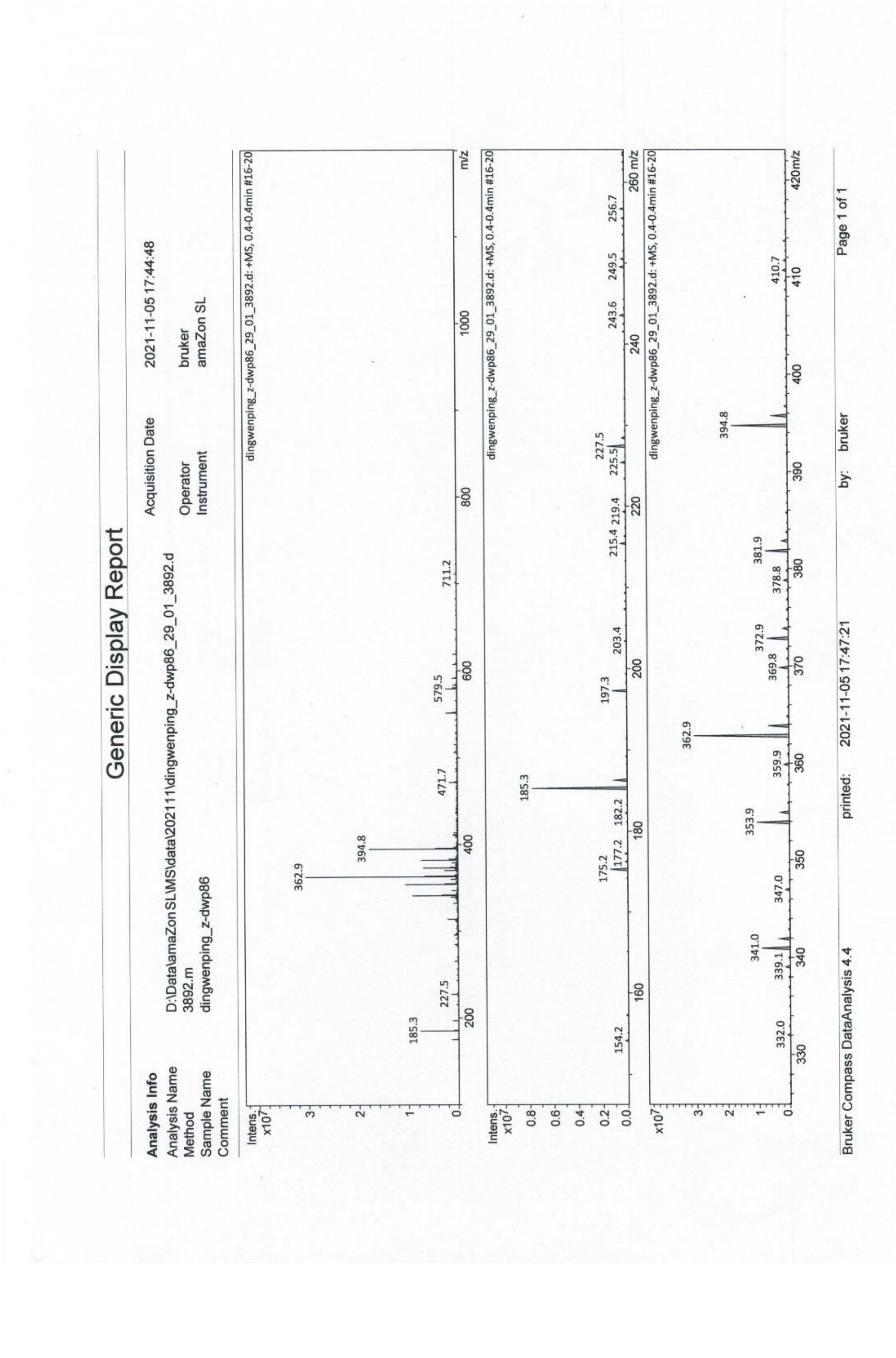

Fig. S76 ^{1}H NMR spectrum (CD_3OD, 700 MHz) of compound **12**

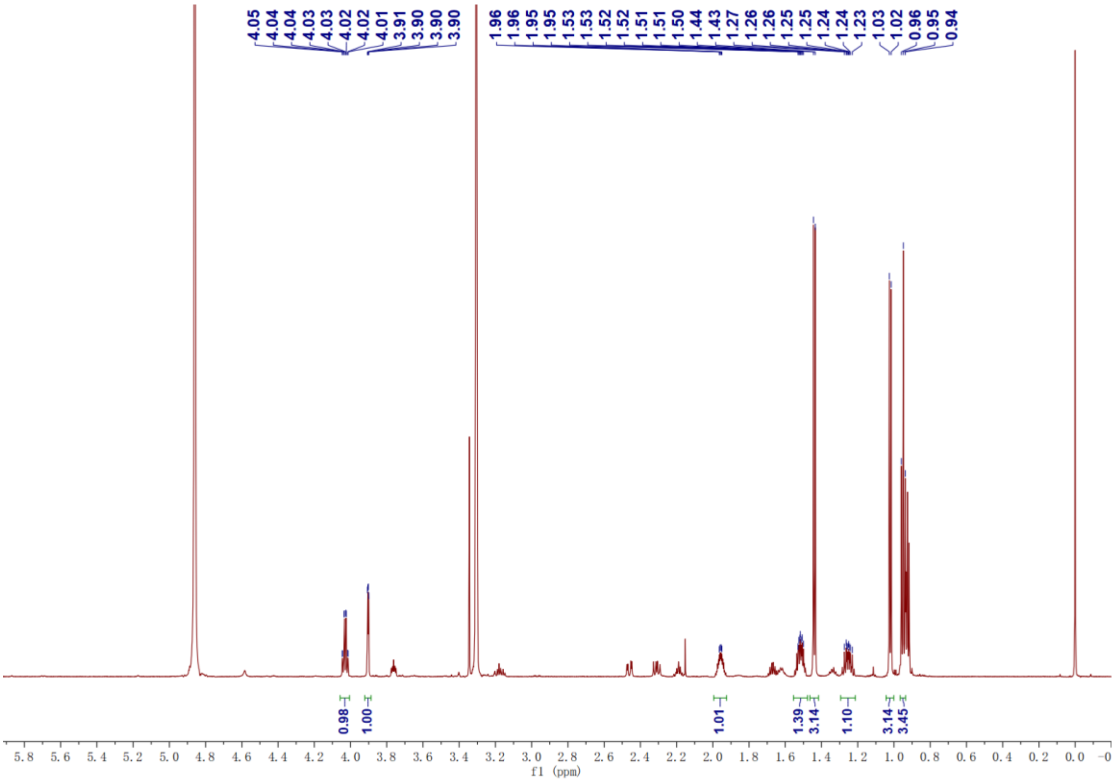

Fig. S77 ^{13}C NMR spectrum (CD_3OD, 176 MHz) of compound **12**

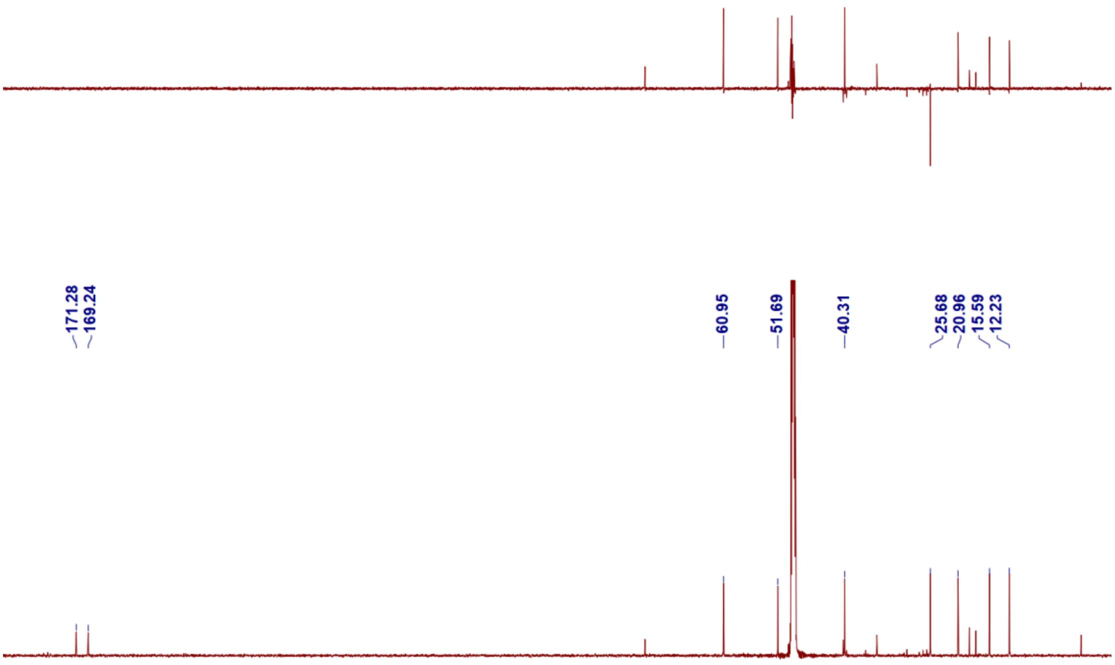

Fig. S78 ESI-MS spectrum of compound **13**

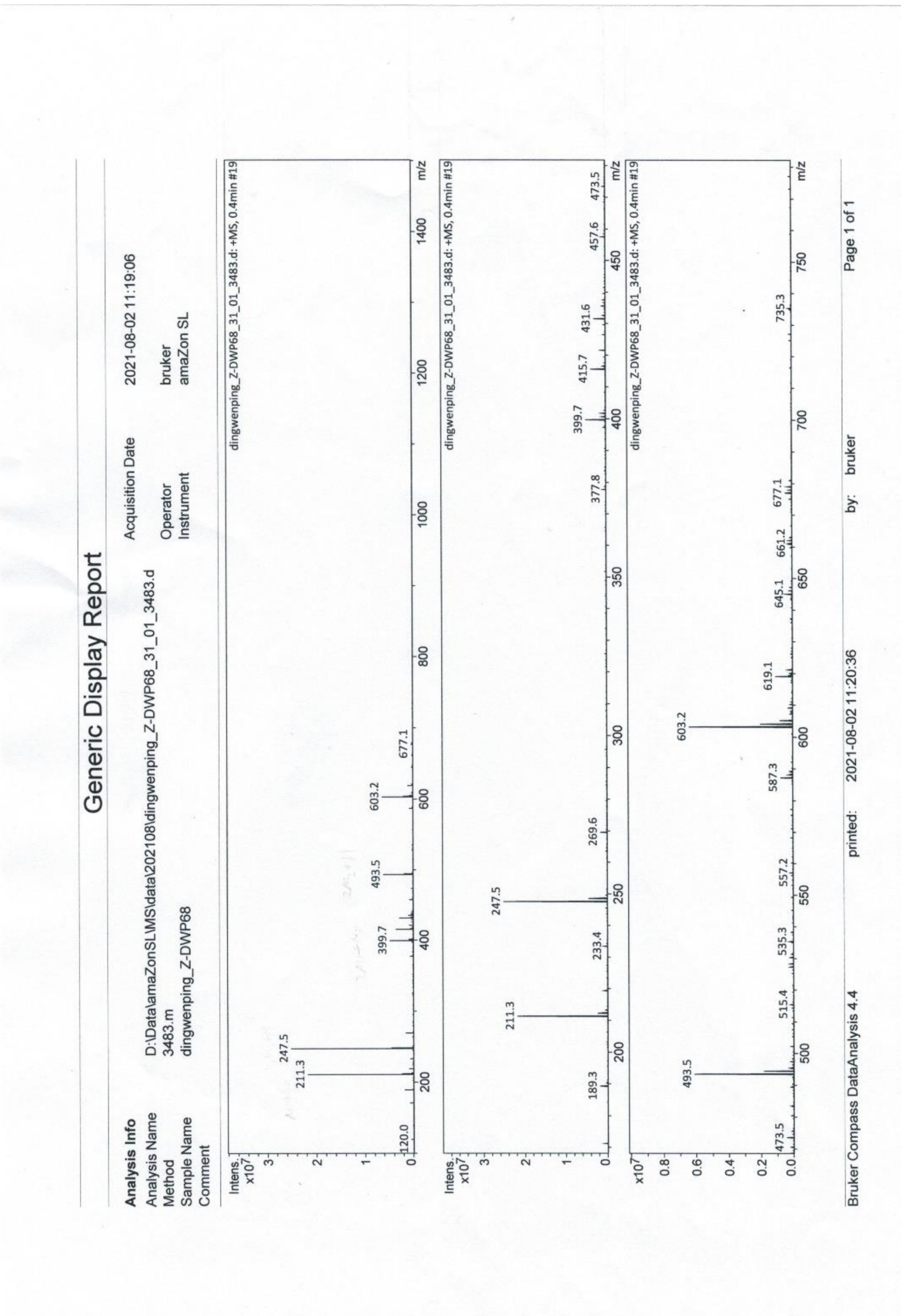

Structure of impurity:

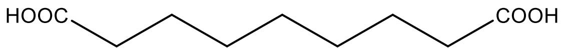

Nonanedioic acid

Fig. S79 ^{1}H NMR spectrum (CD_3OD, 700 MHz) of compound **13**

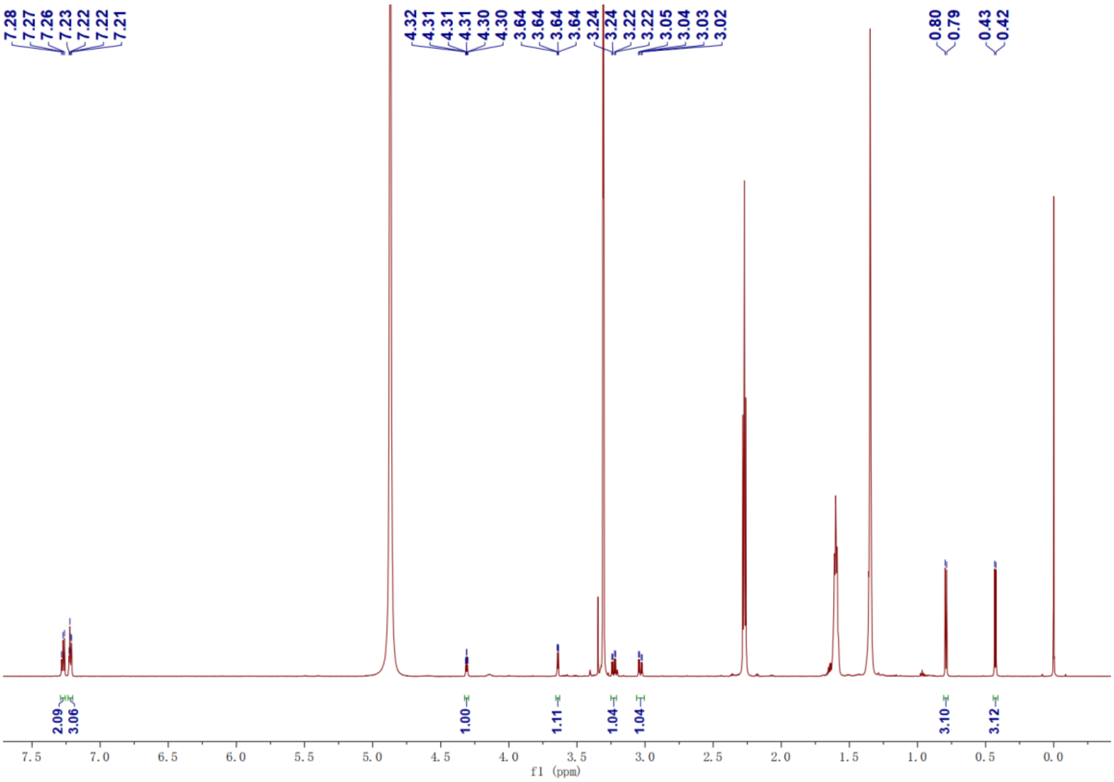

Fig. S80 ^{13}C NMR spectrum (CD_3OD, 176 MHz) of compound **13**

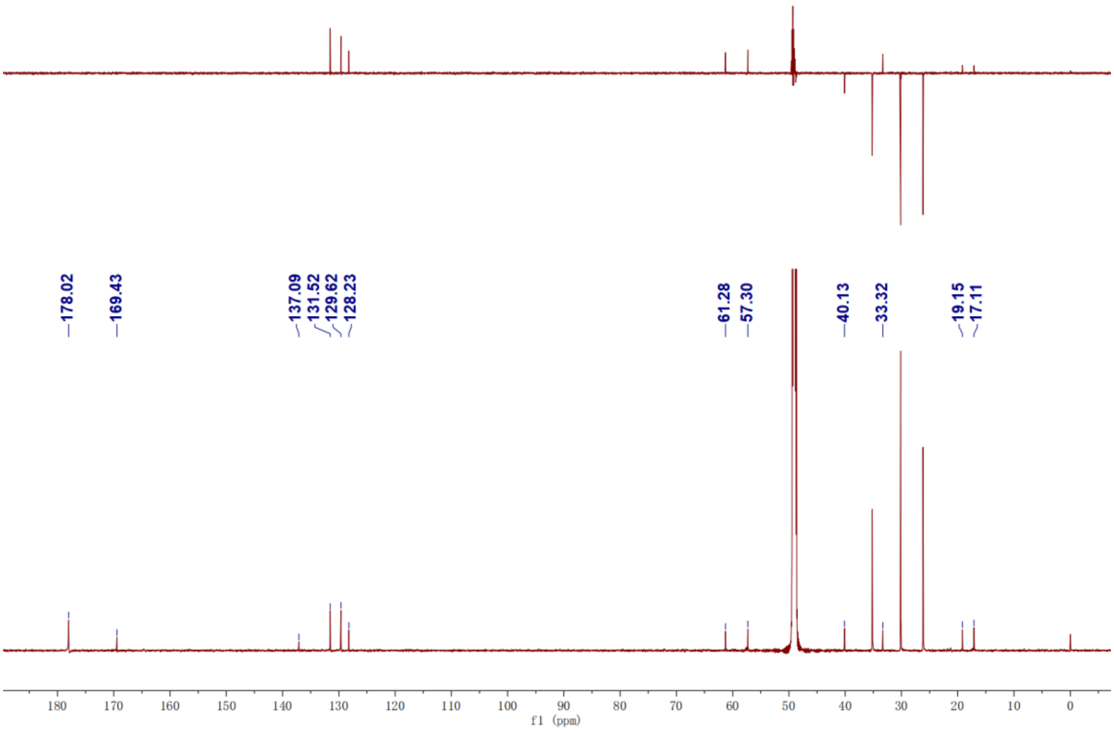

Fig. S81 ESI-MS spectrum of compound **14**

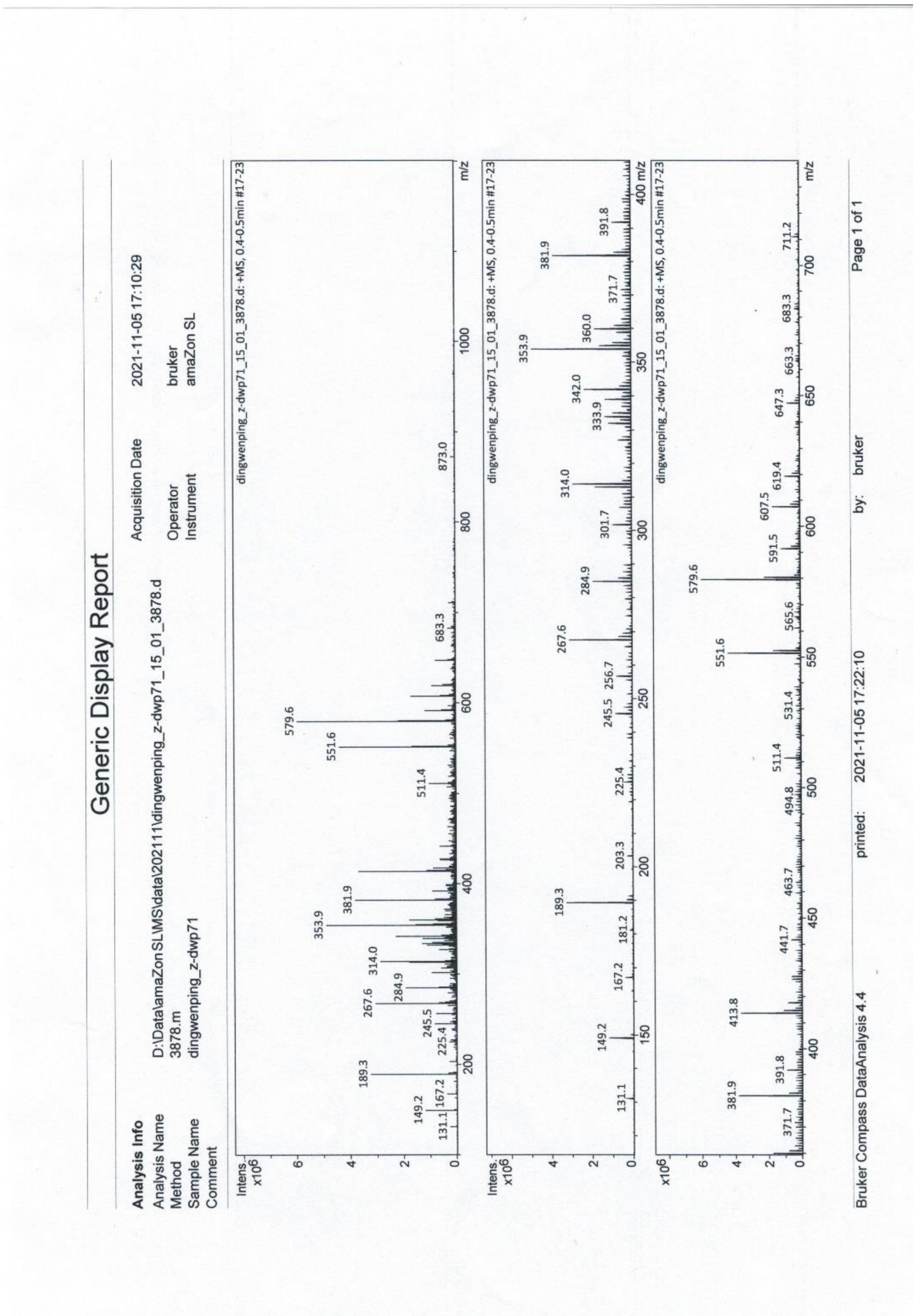

Fig. S82 ^{1}H NMR spectrum (CD_3Cl, 700 MHz) of compound **14**

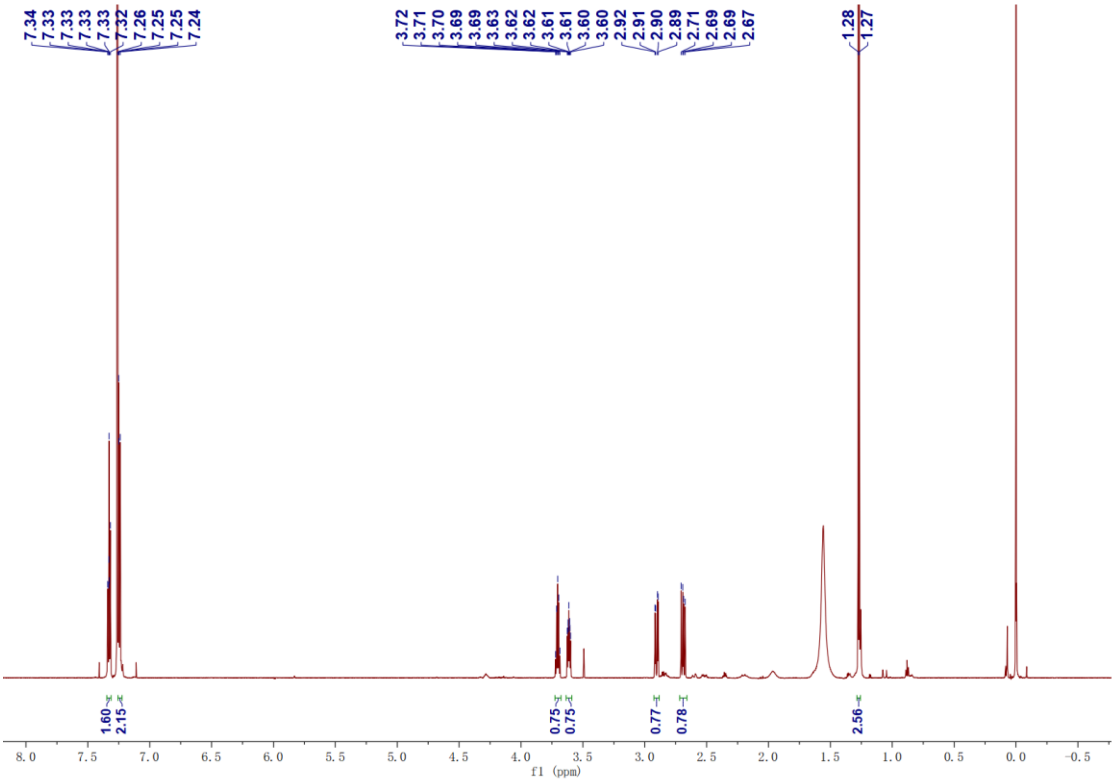

Fig. S83 ^{13}C NMR spectrum (CD_3Cl, 176 MHz) of compound **14**

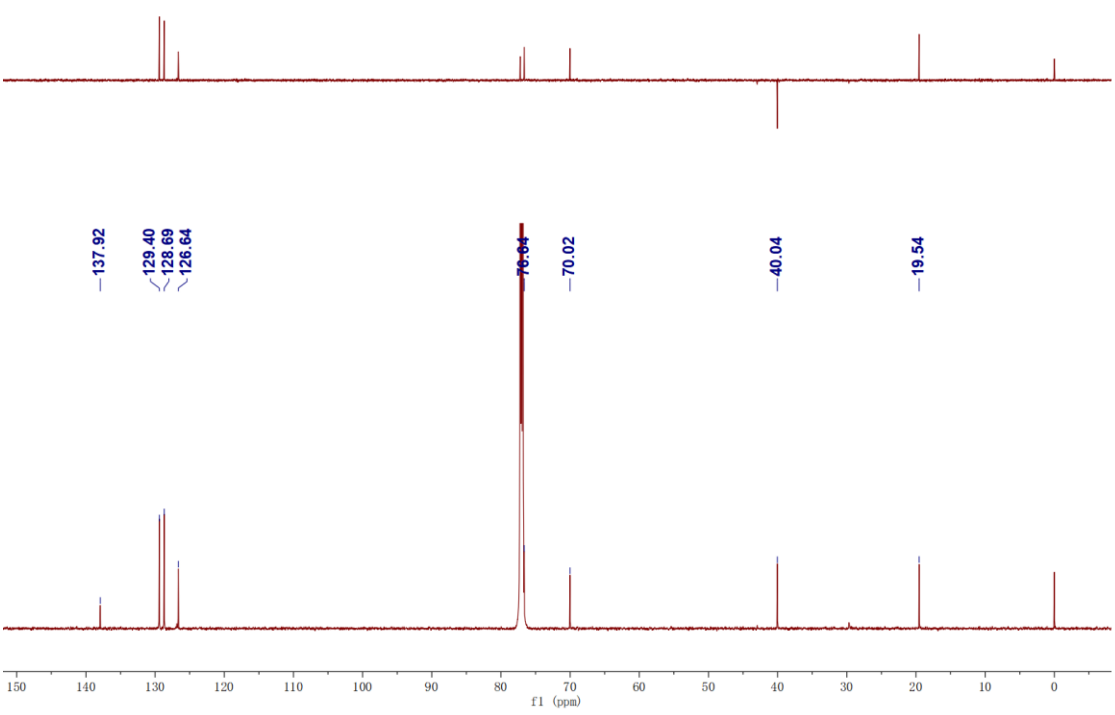

Fig. S84 ESI-MS spectrum of compound **15**

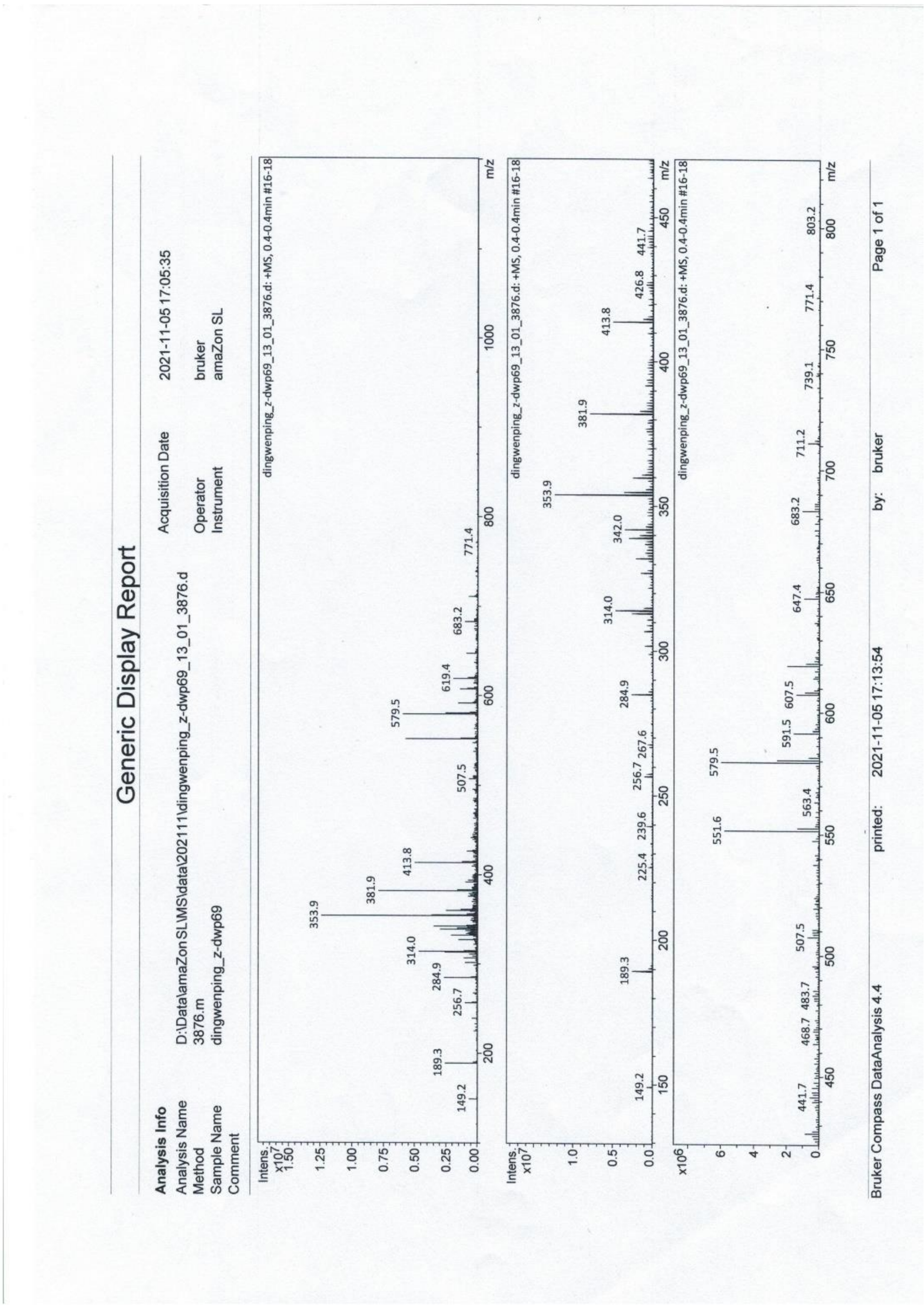

Fig. S85 ^{1}H NMR spectrum (CD_3Cl, 700 MHz) of compound **15**

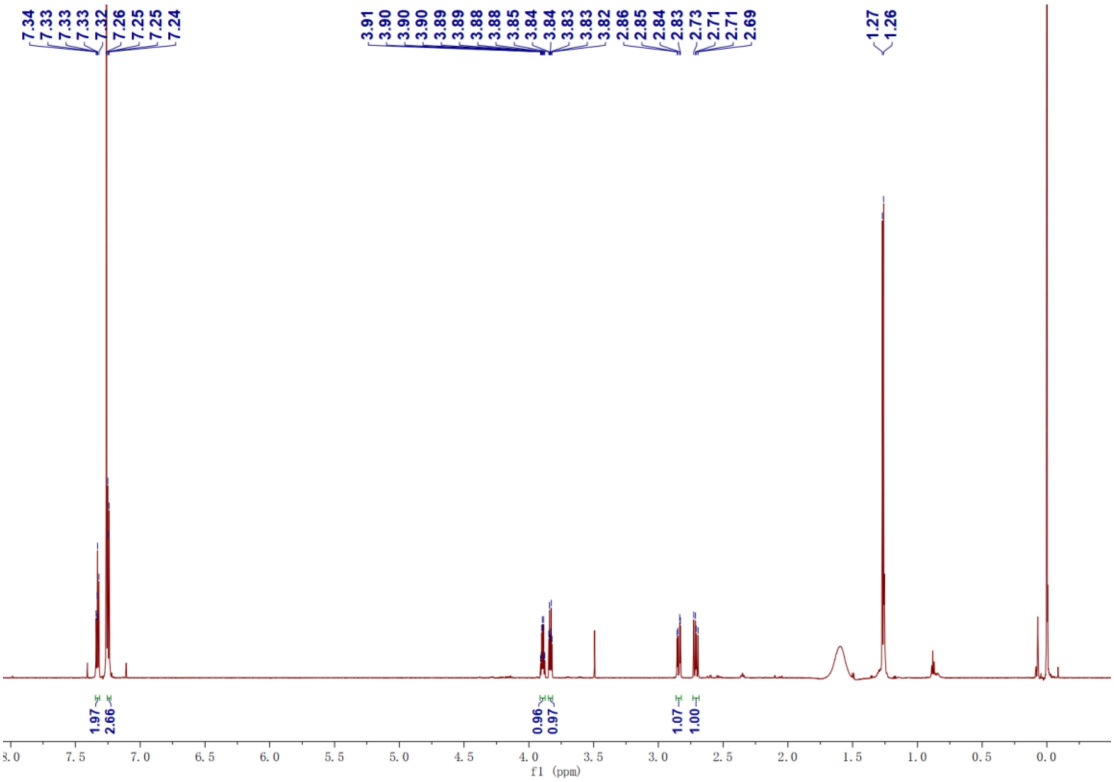

Fig. S86 ^{13}C NMR spectrum (CD_3Cl, 176 MHz) of compound **15**

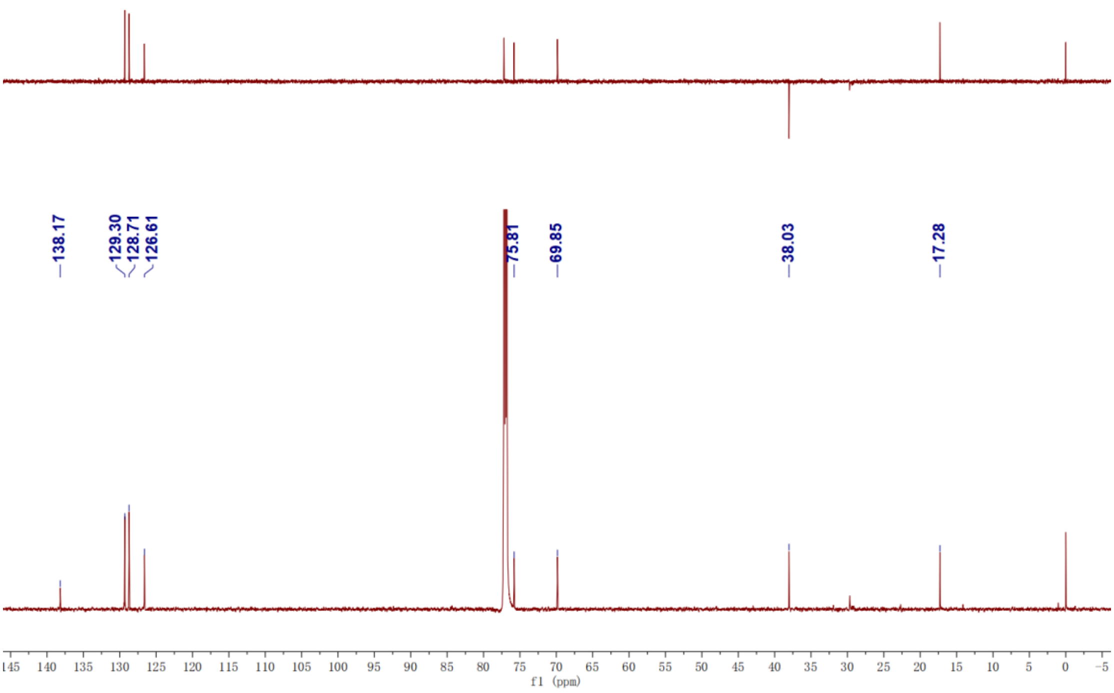

Fig. S87 ESI-MS spectrum of compound **16**

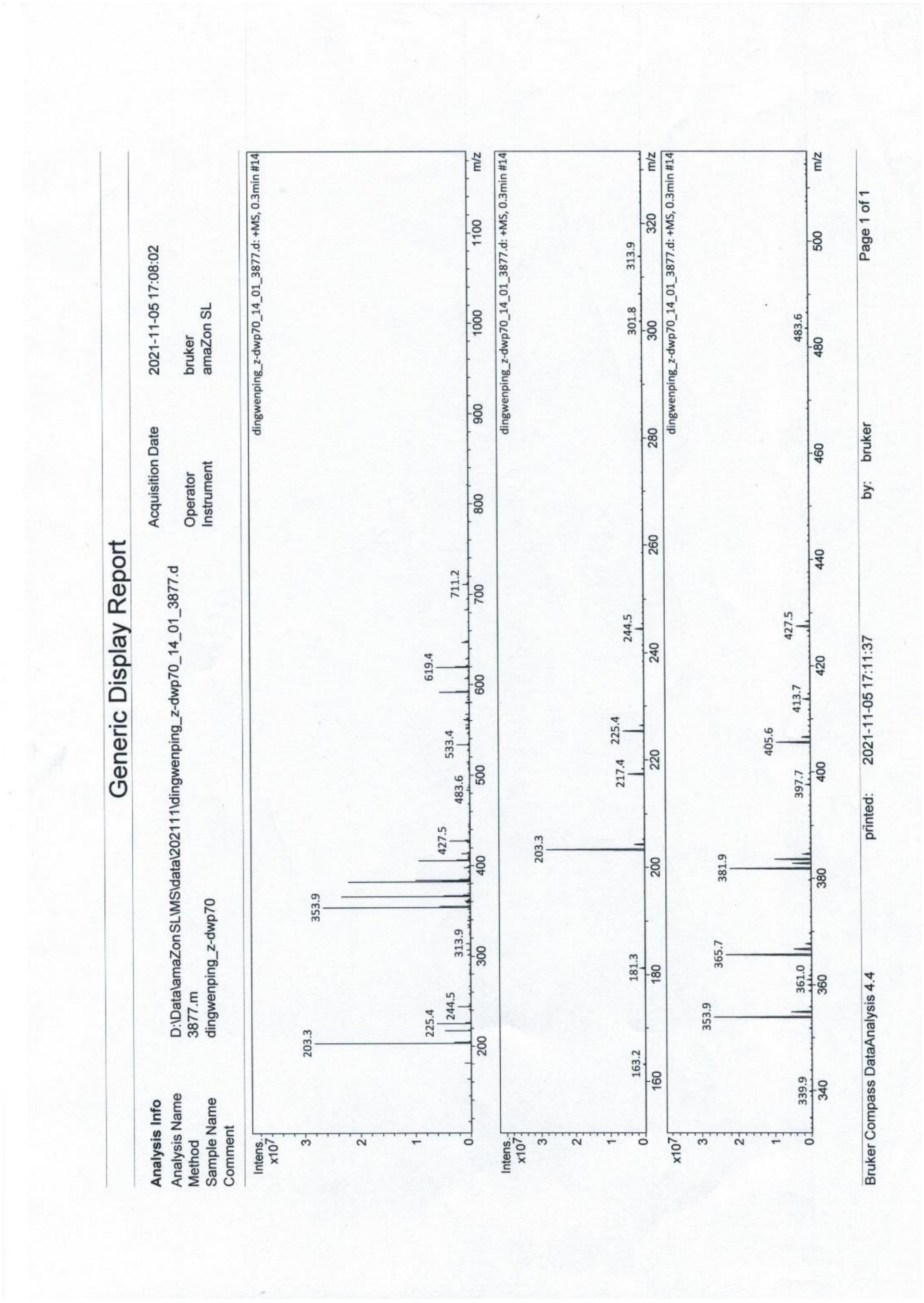

Fig. S88 ^{1}H NMR spectrum (CD_3OD, 700 MHz) of compound **16**

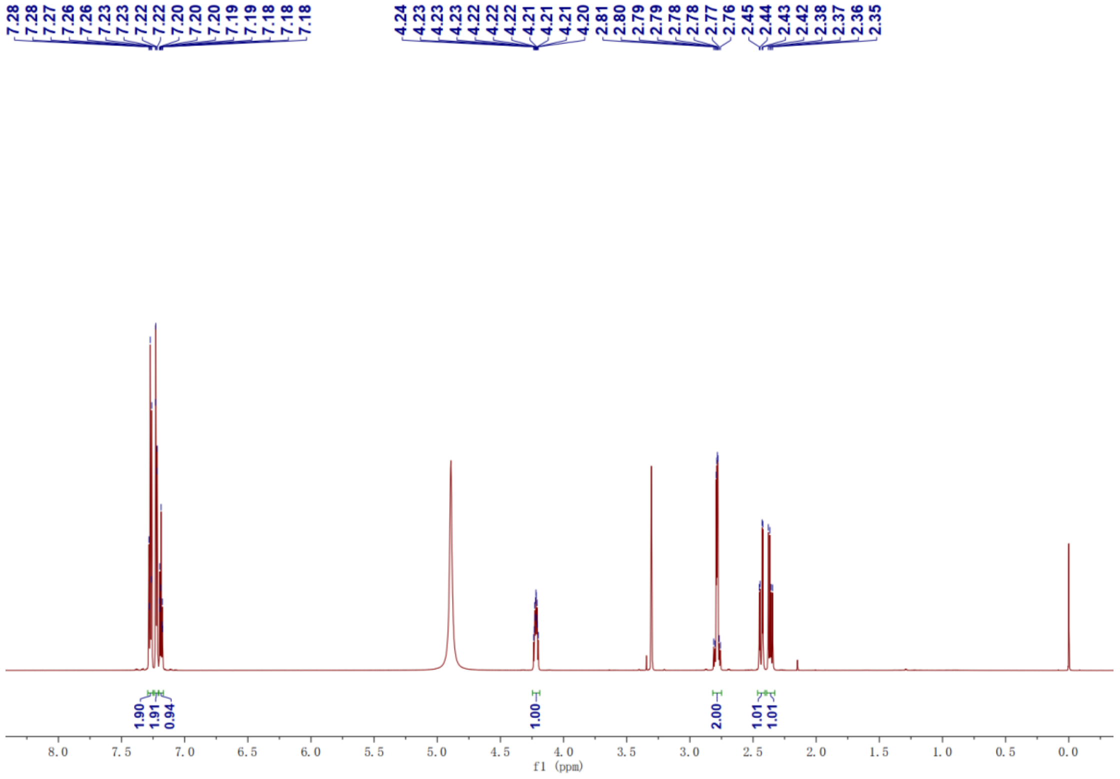

Fig. S89 ^{13}C NMR spectrum (CD_3OD, 176 MHz) of compound **16**

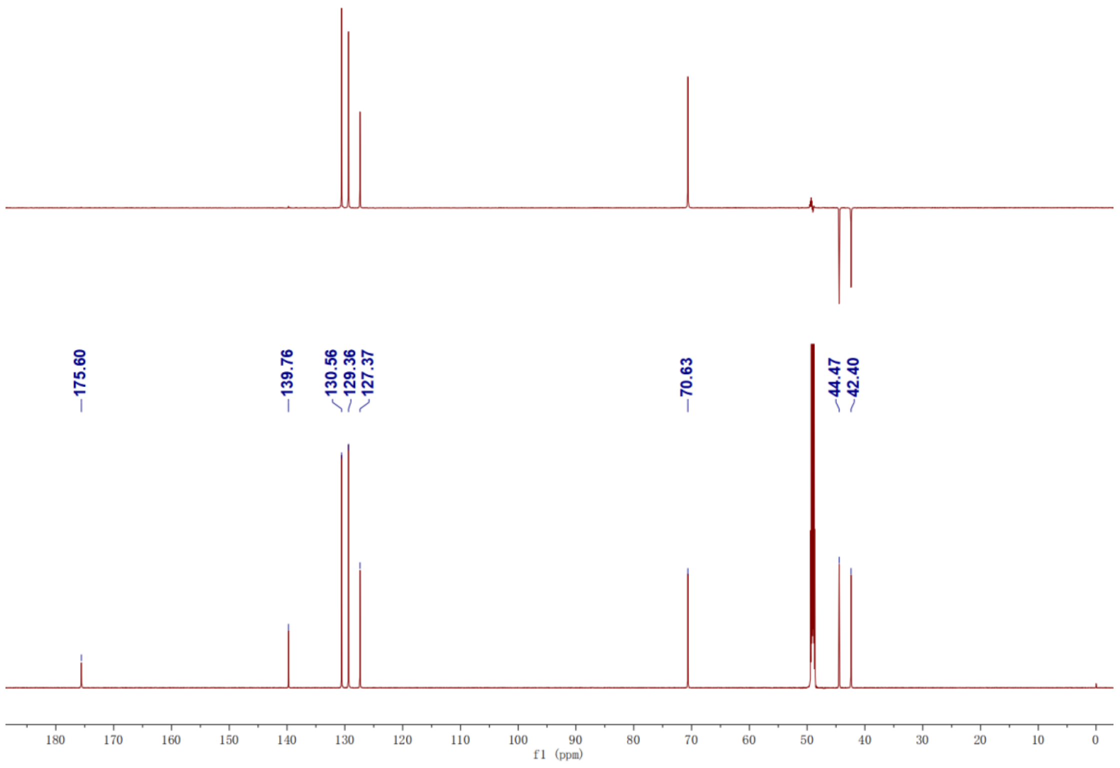

Fig. S90 ESI-MS spectrum of compound **17**

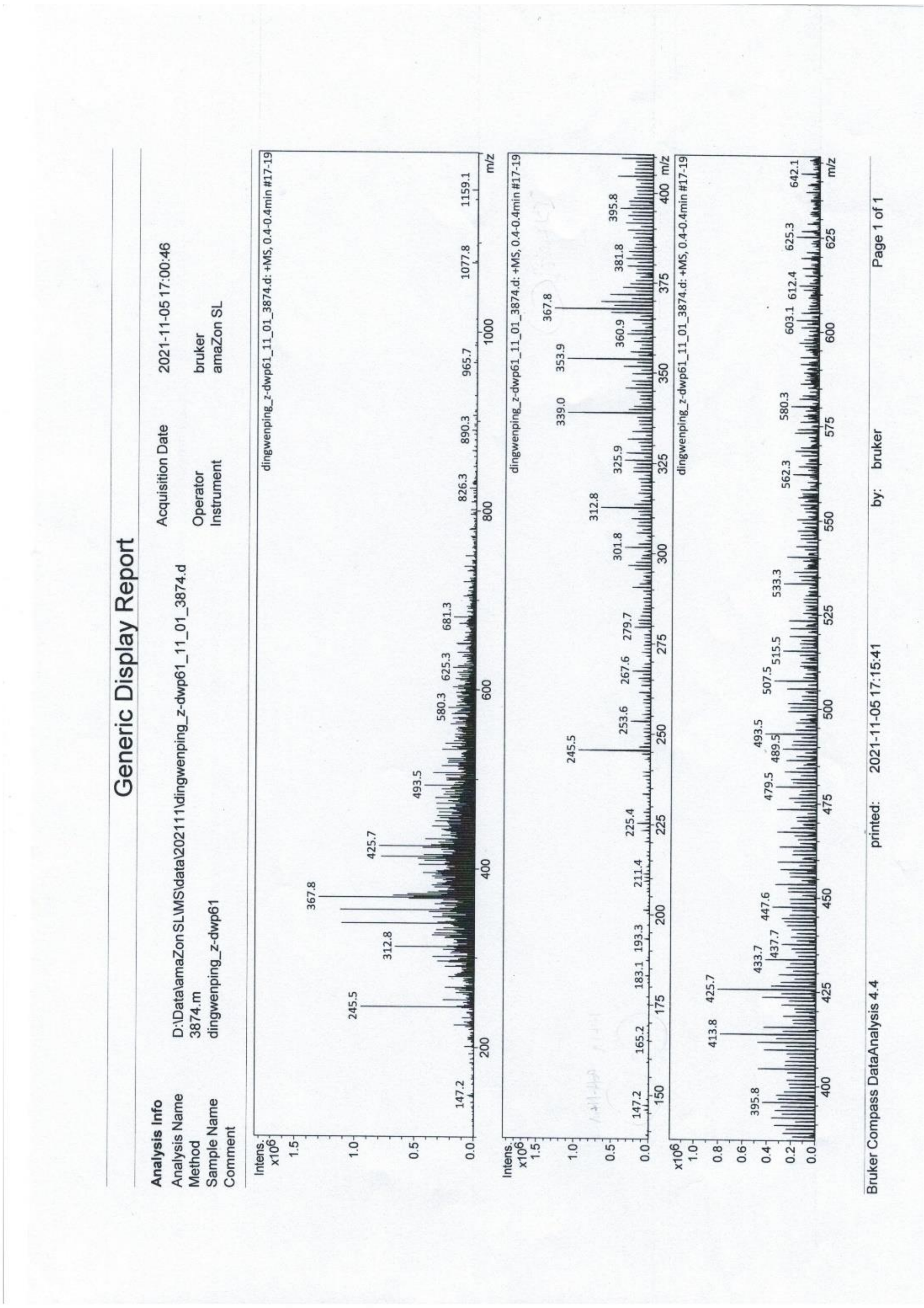

Fig. S91 ^{1}H NMR spectrum (CD_3OD, 700 MHz) of compound **17**

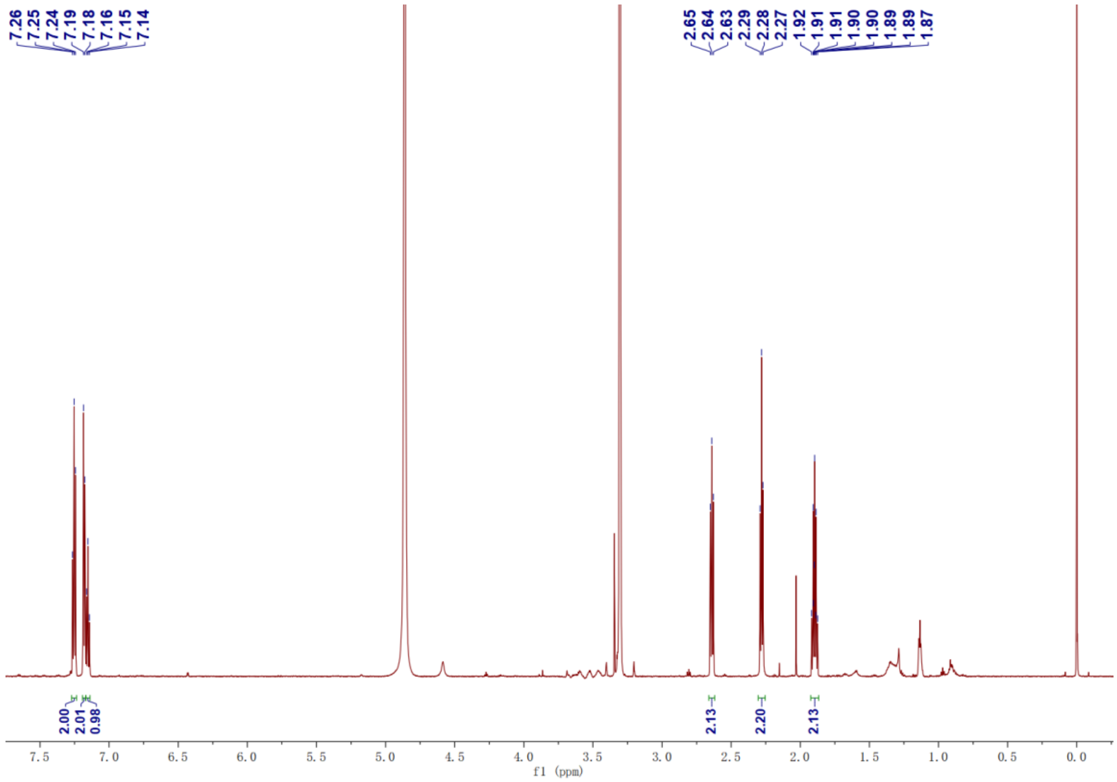

Fig. S92 ^{13}C NMR spectrum (CD_3OD, 176 MHz) of compound **17**

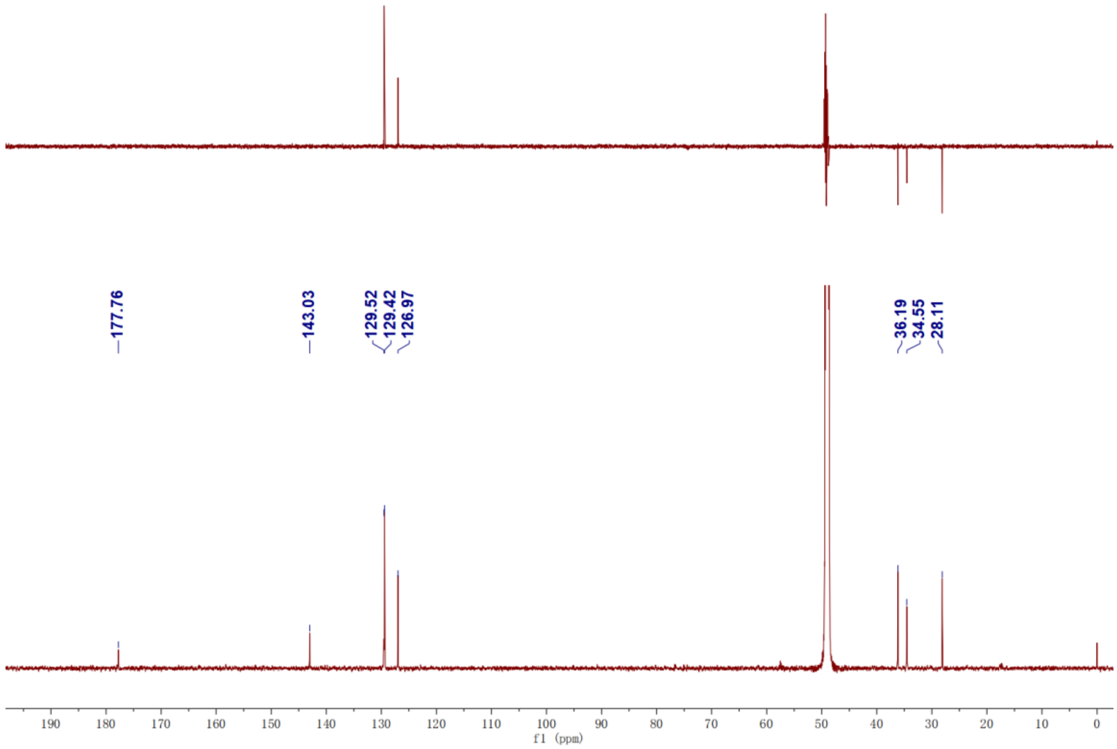

Fig. S93 ESI-MS spectrum of compound **18**

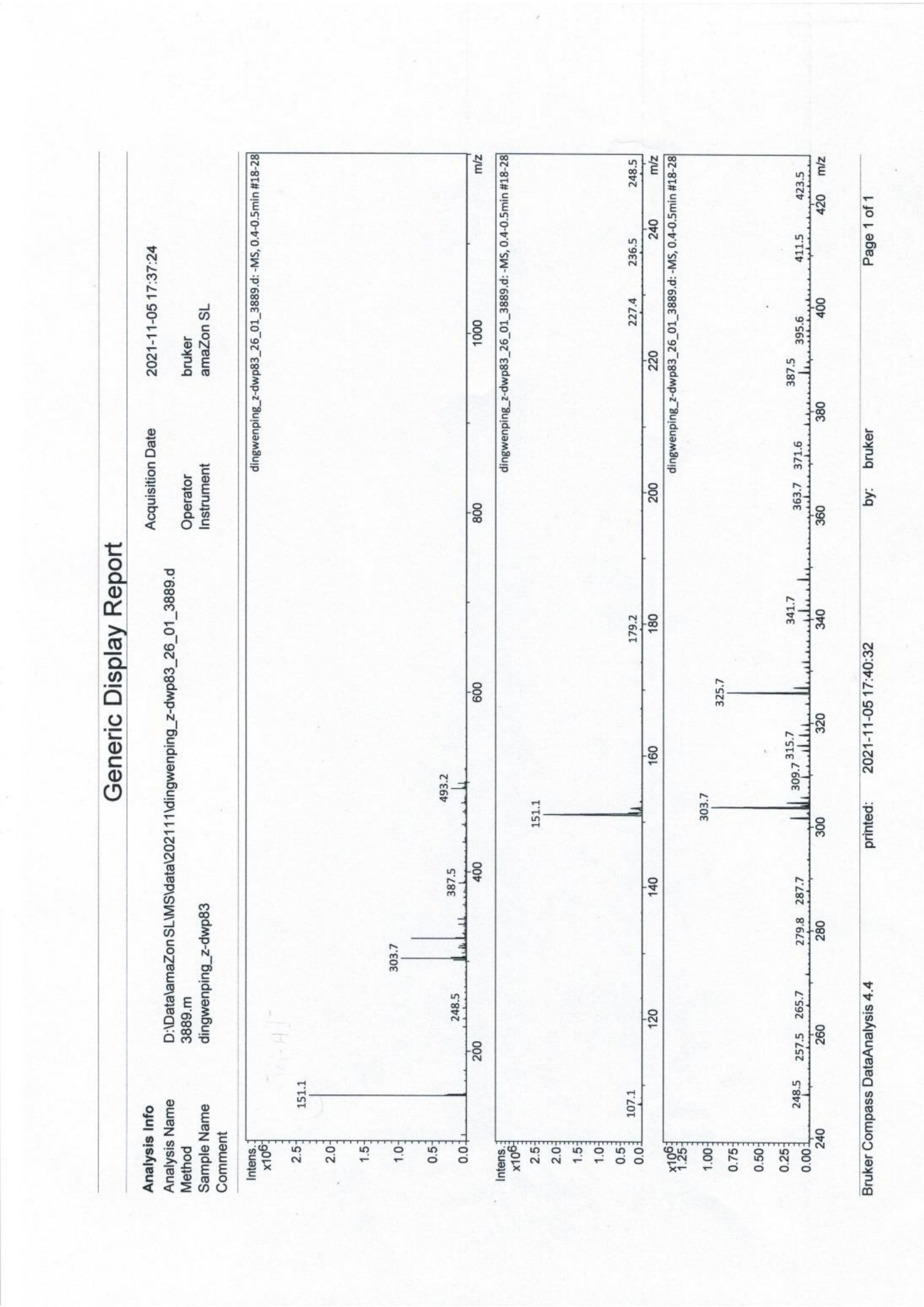

Fig. S94 ^{1}H NMR spectrum (CD_3OD, 500 MHz) of compound **18**

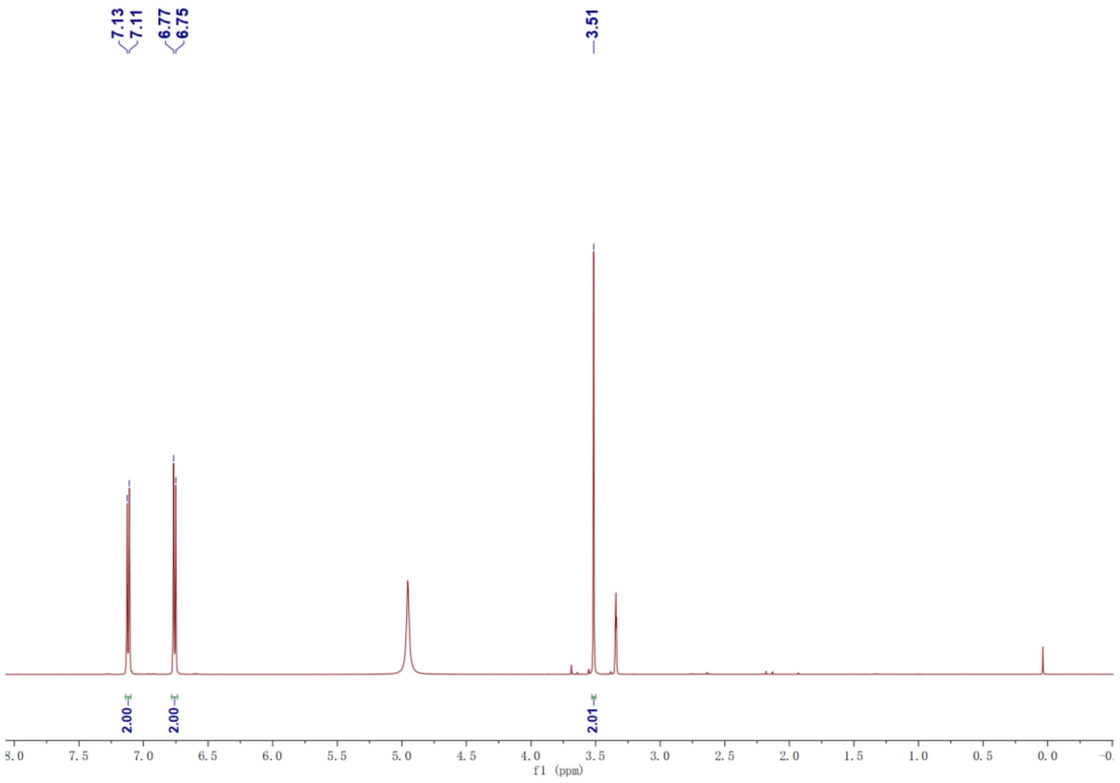

Fig. S95 ^{13}C NMR spectrum (CD_3OD, 126 MHz) of compound **18**

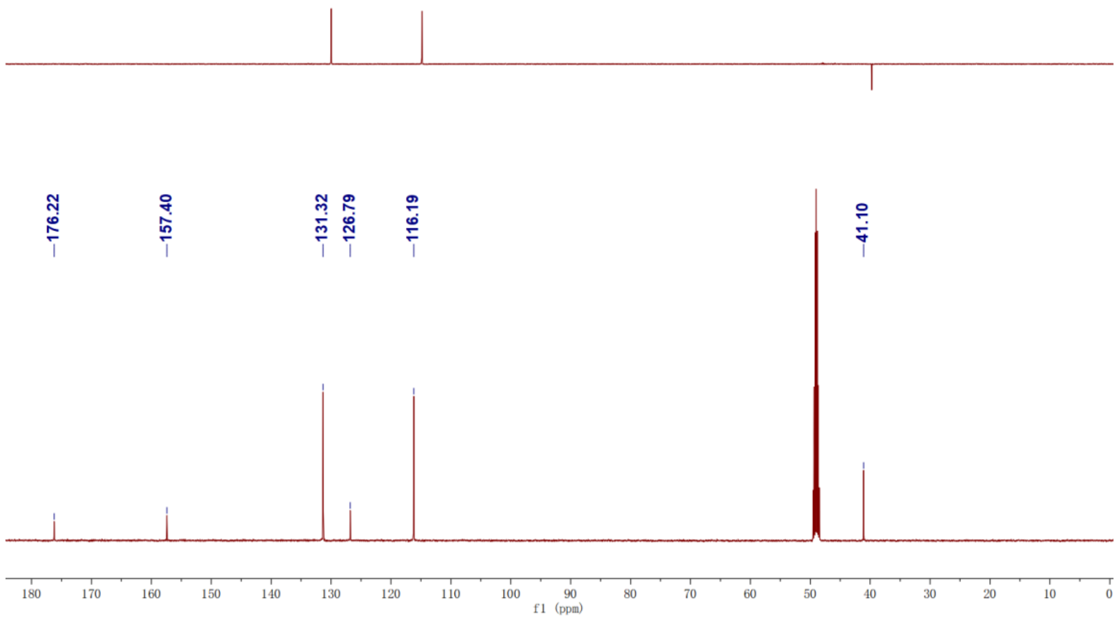

Fig. S96 ESI-MS spectrum of compound **19**

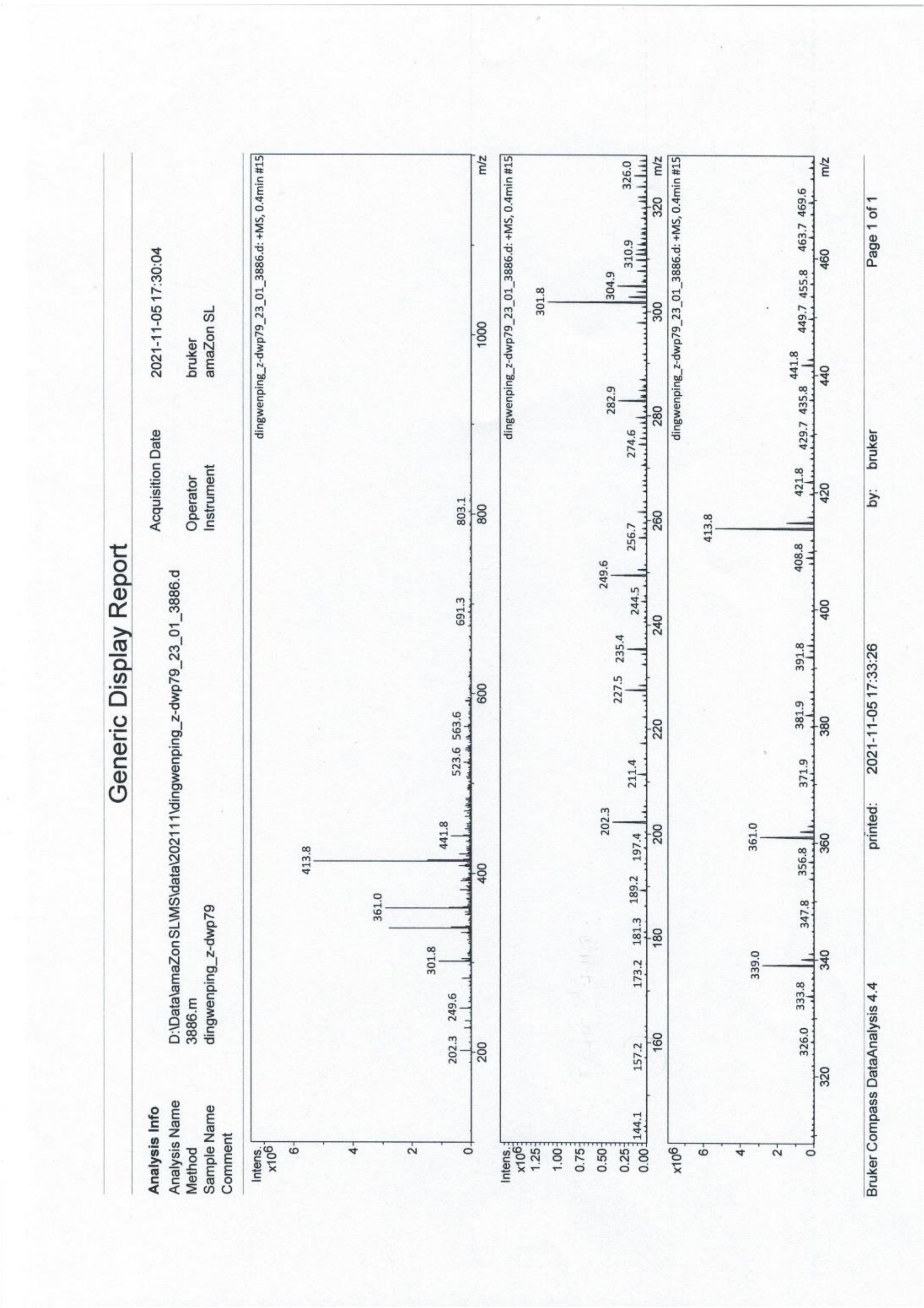

Fig. S97 ^{1}H NMR spectrum (CD_3OD, 500 MHz) of compound **19**

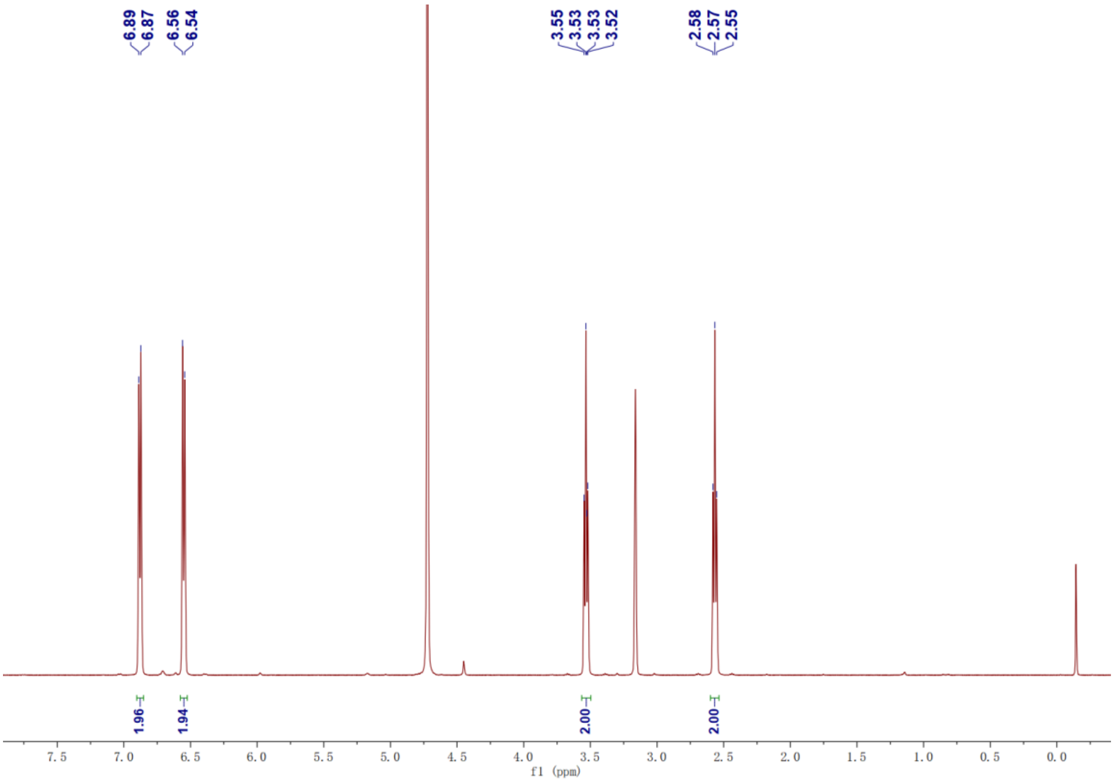

Fig. S98 ^{13}C NMR spectrum (CD_3OD, 126 MHz) of compound **19**

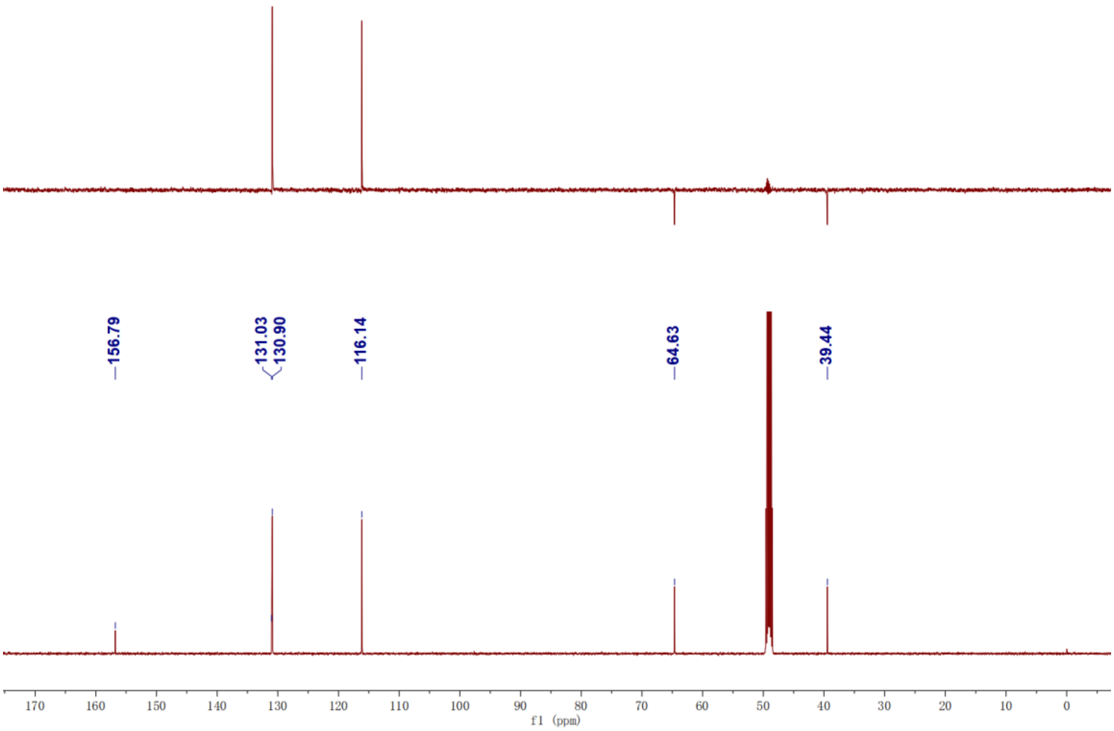

Fig. S99 ESI-MS spectrum of compound **20**

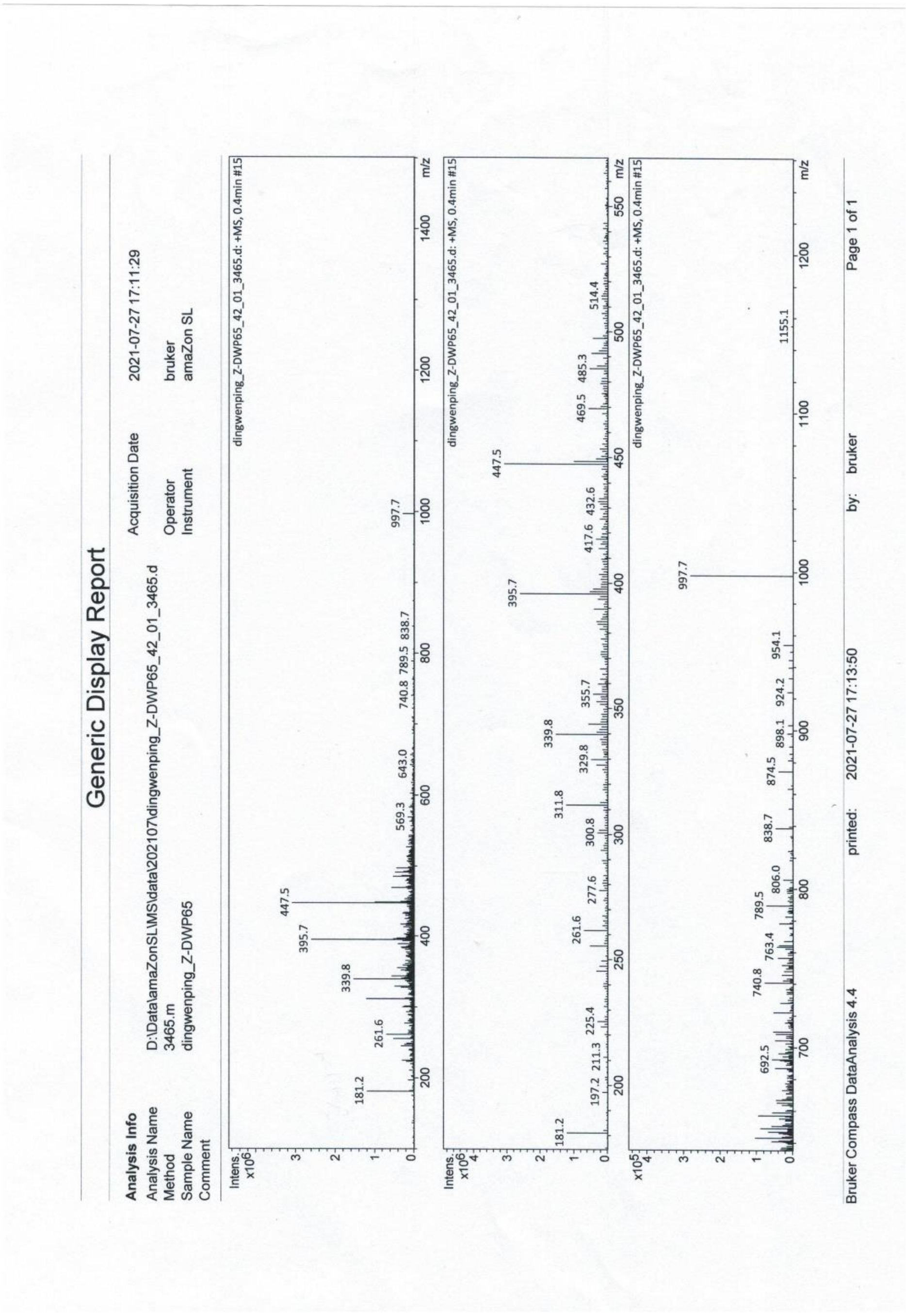

Fig. S100 ^{1}H NMR spectrum (CD_3OD, 500 MHz) of compound **20**

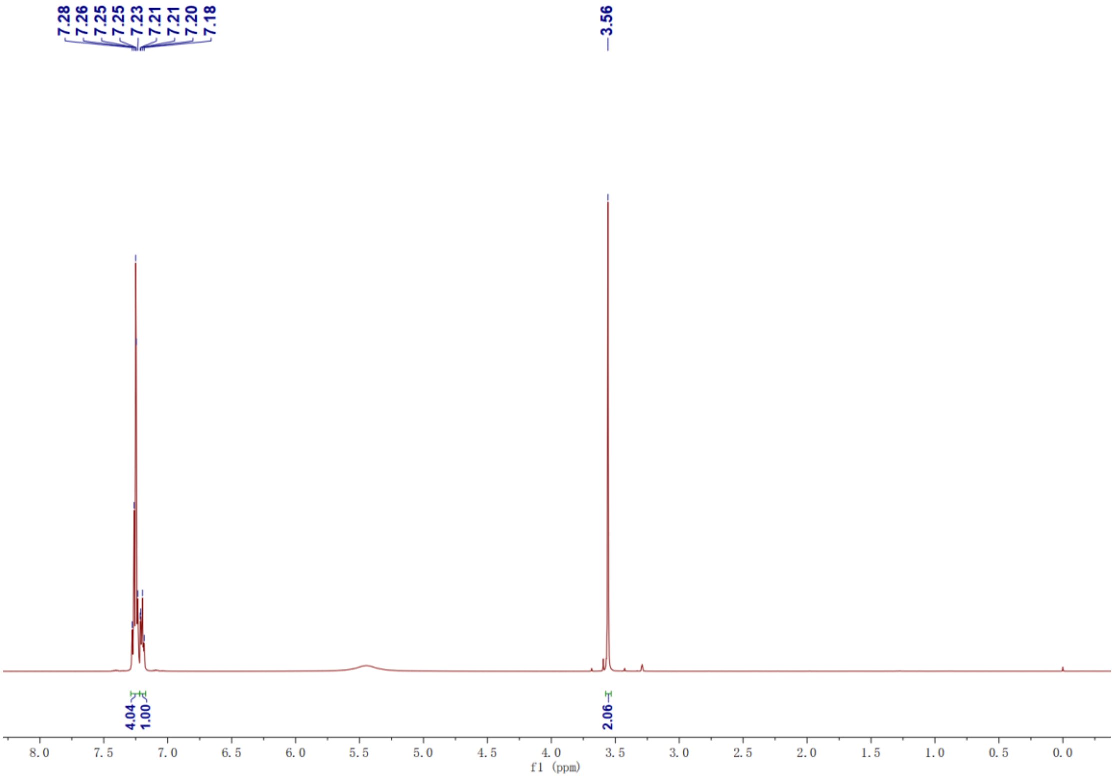

Fig. S101 ^{13}C NMR spectrum (CD_3OD, 126 MHz) of compound **20**

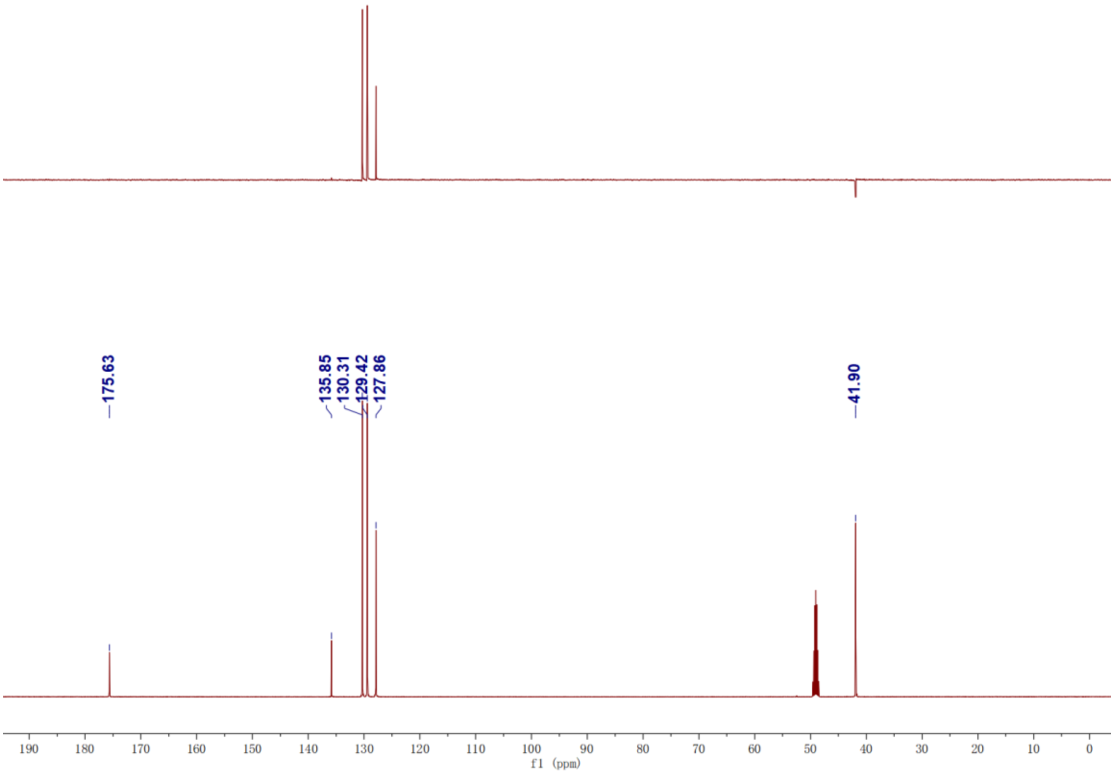

Fig. S102 ESI-MS spectrum of compound **21**

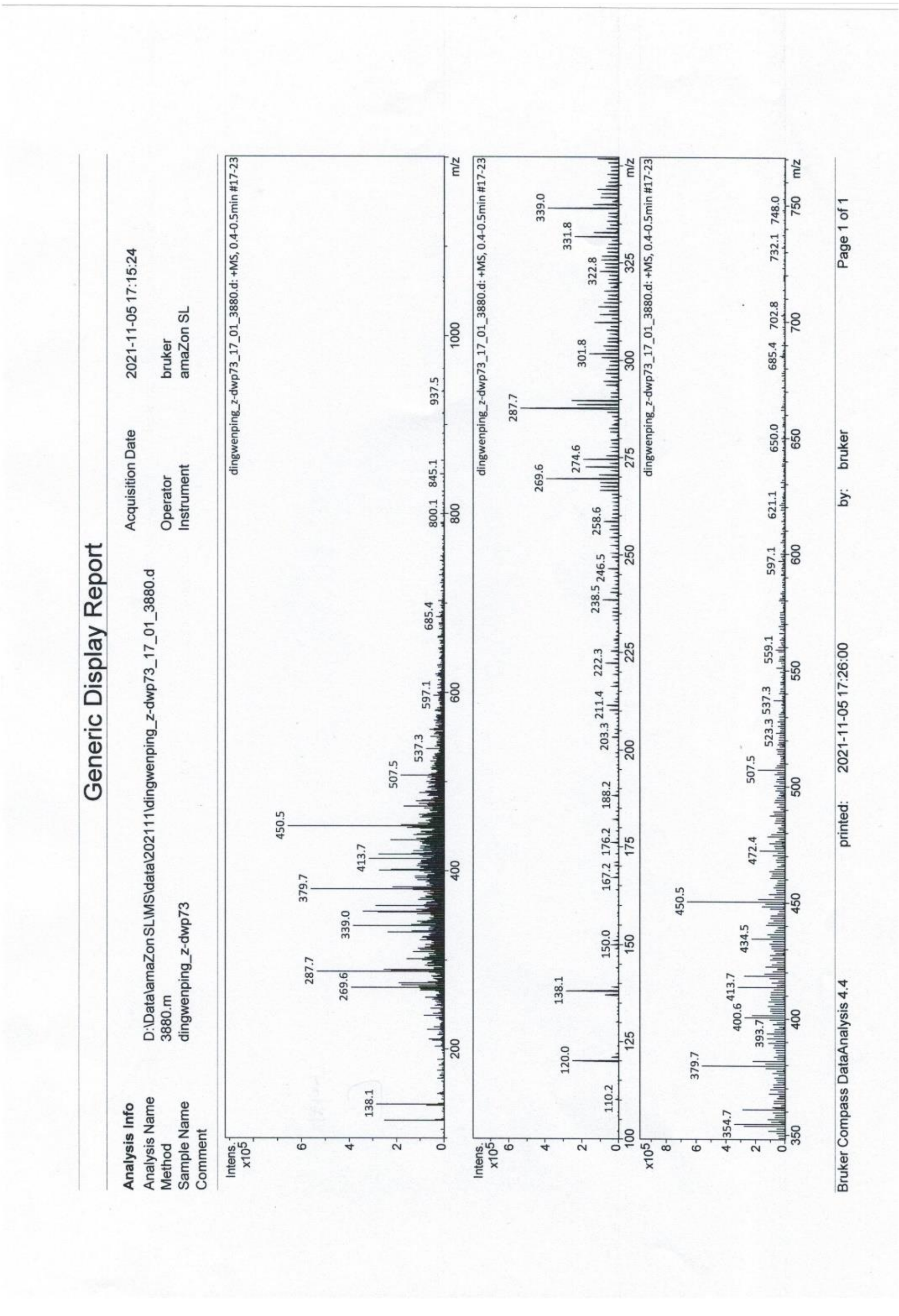

Fig. S103 ^{1}H NMR spectrum (CD_3OD, 500 MHz) of compound **21**

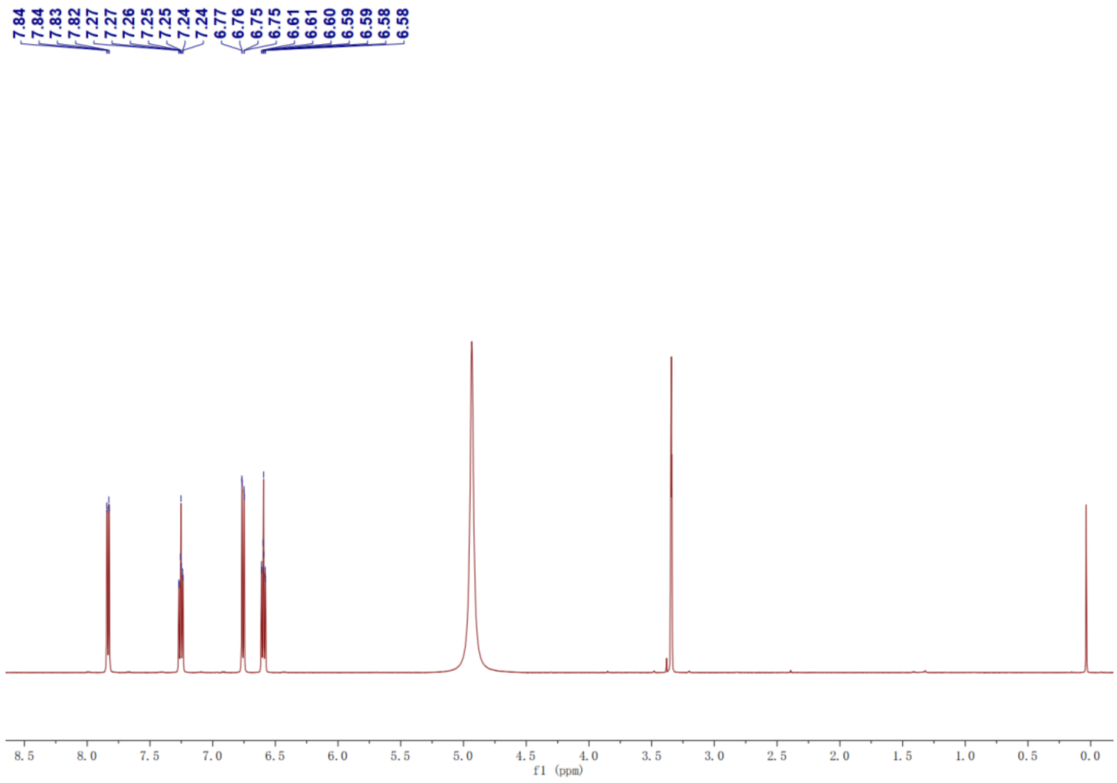

Fig. S104 ^{13}C NMR spectrum (CD_3OD, 126 MHz) of compound **21**

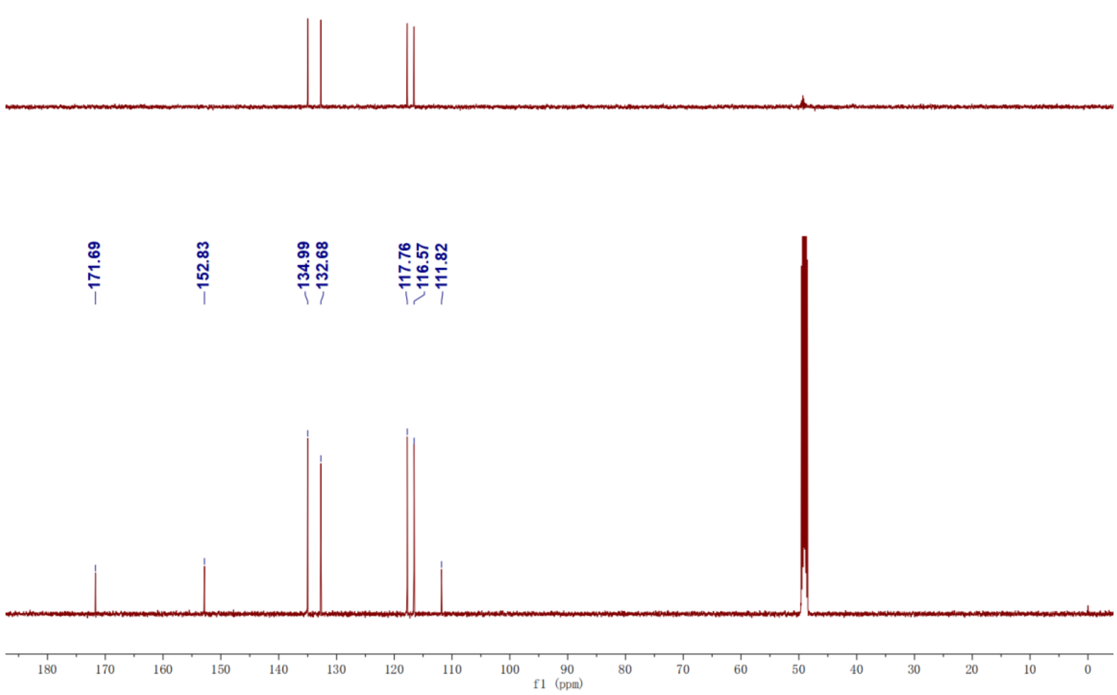

Fig. S105 ESI-MS spectrum of compound **22**

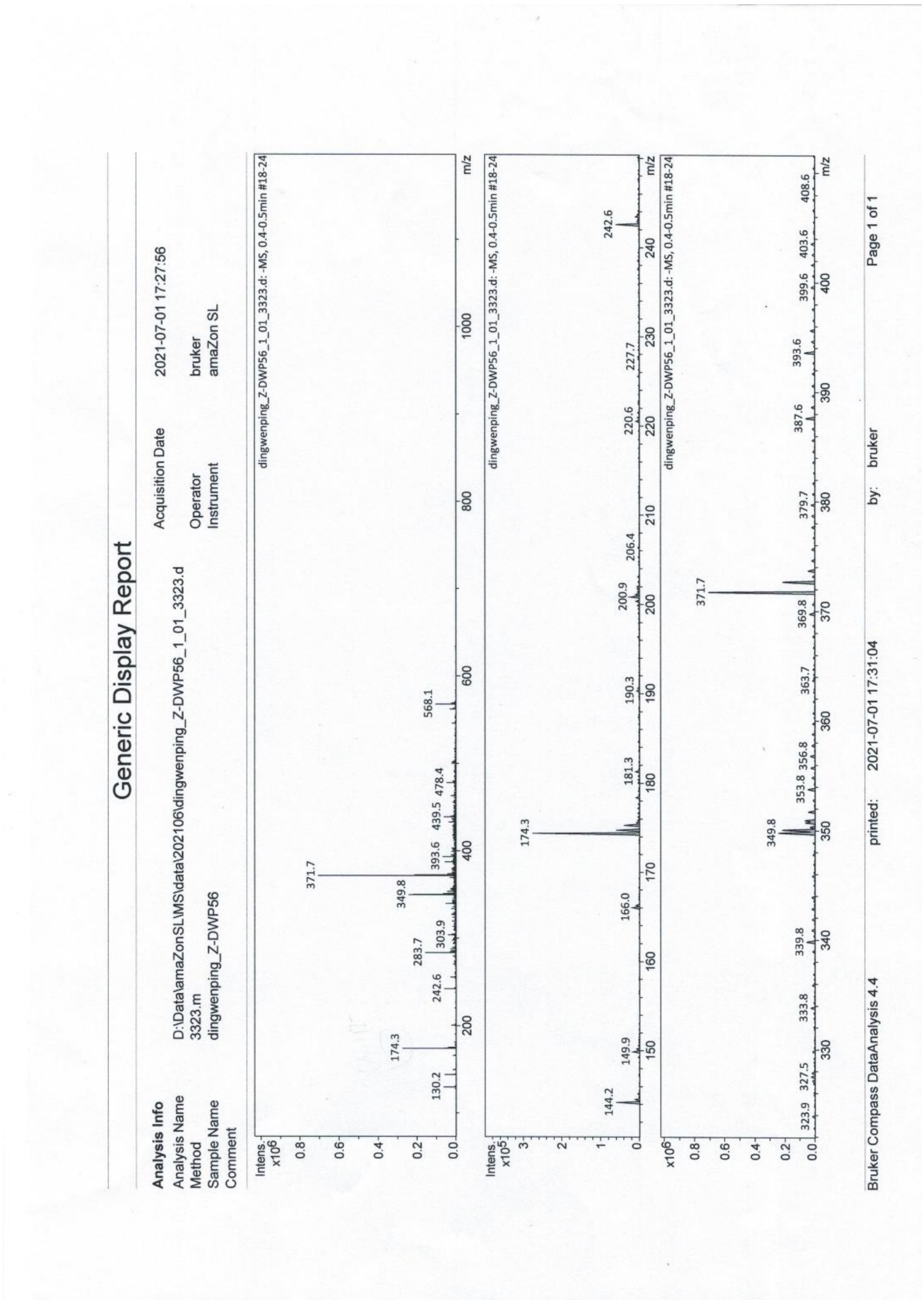

Fig. S106 ^{1}H NMR spectrum (CD_3OD, 700 MHz) of compound **22**

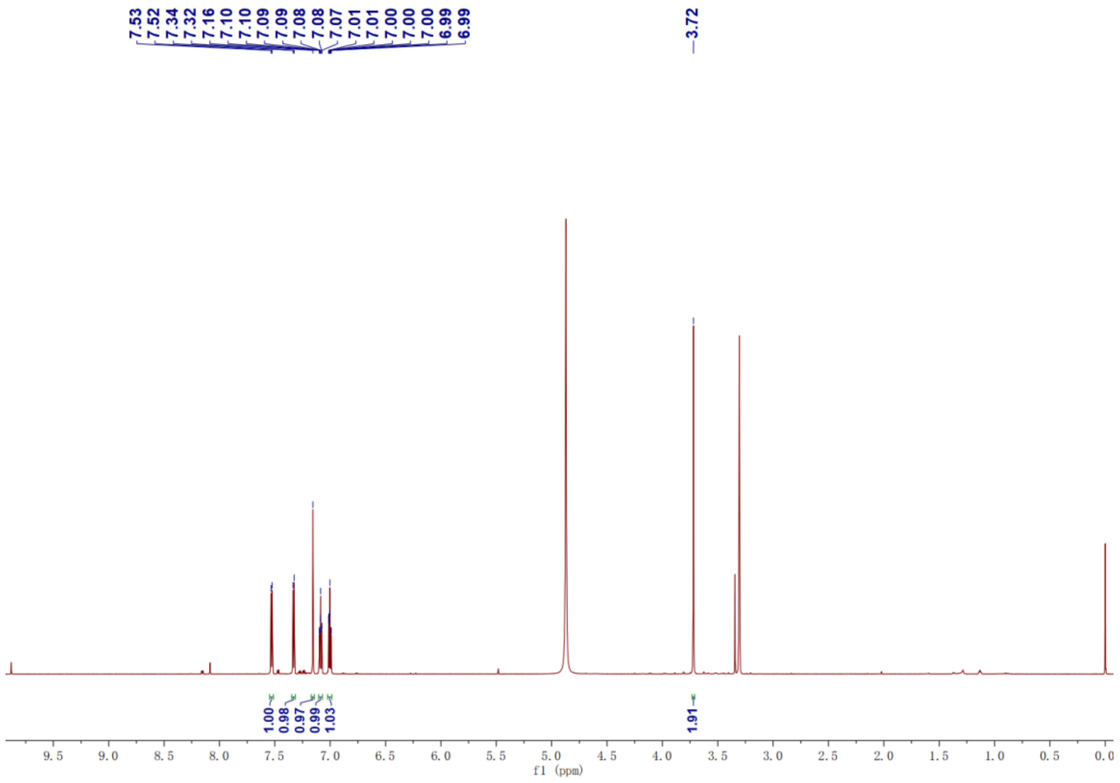

Fig. S107 ^{13}C NMR spectrum (CD_3OD, 176 MHz) of compound **22**

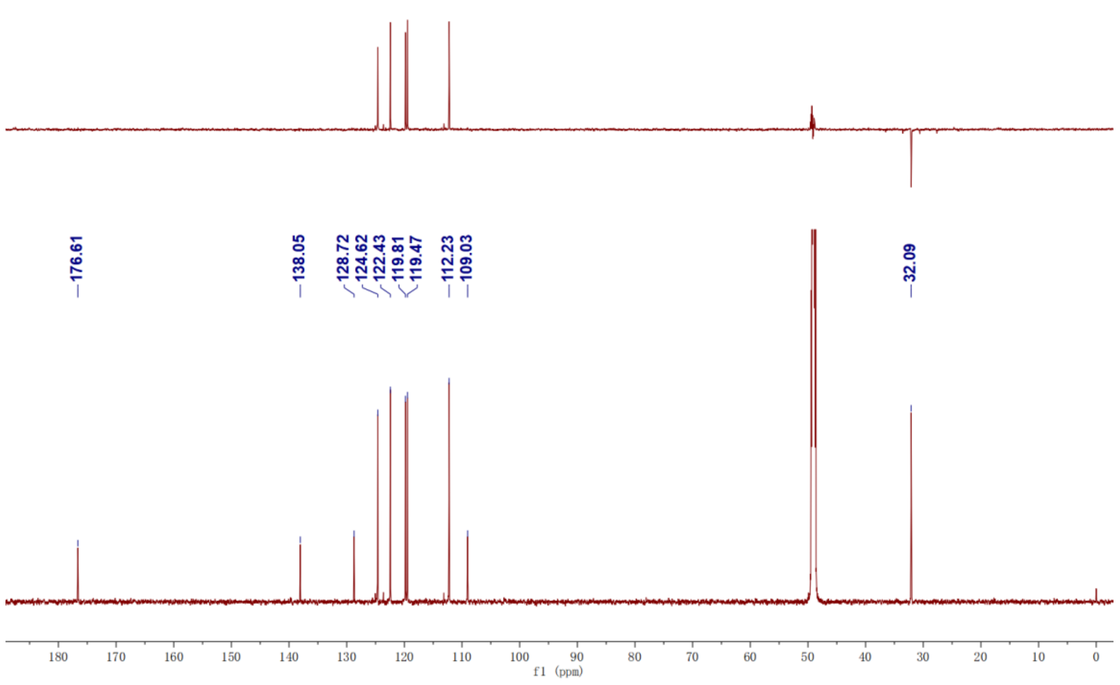

Fig. S108 ESI-MS spectrum of compound **23**

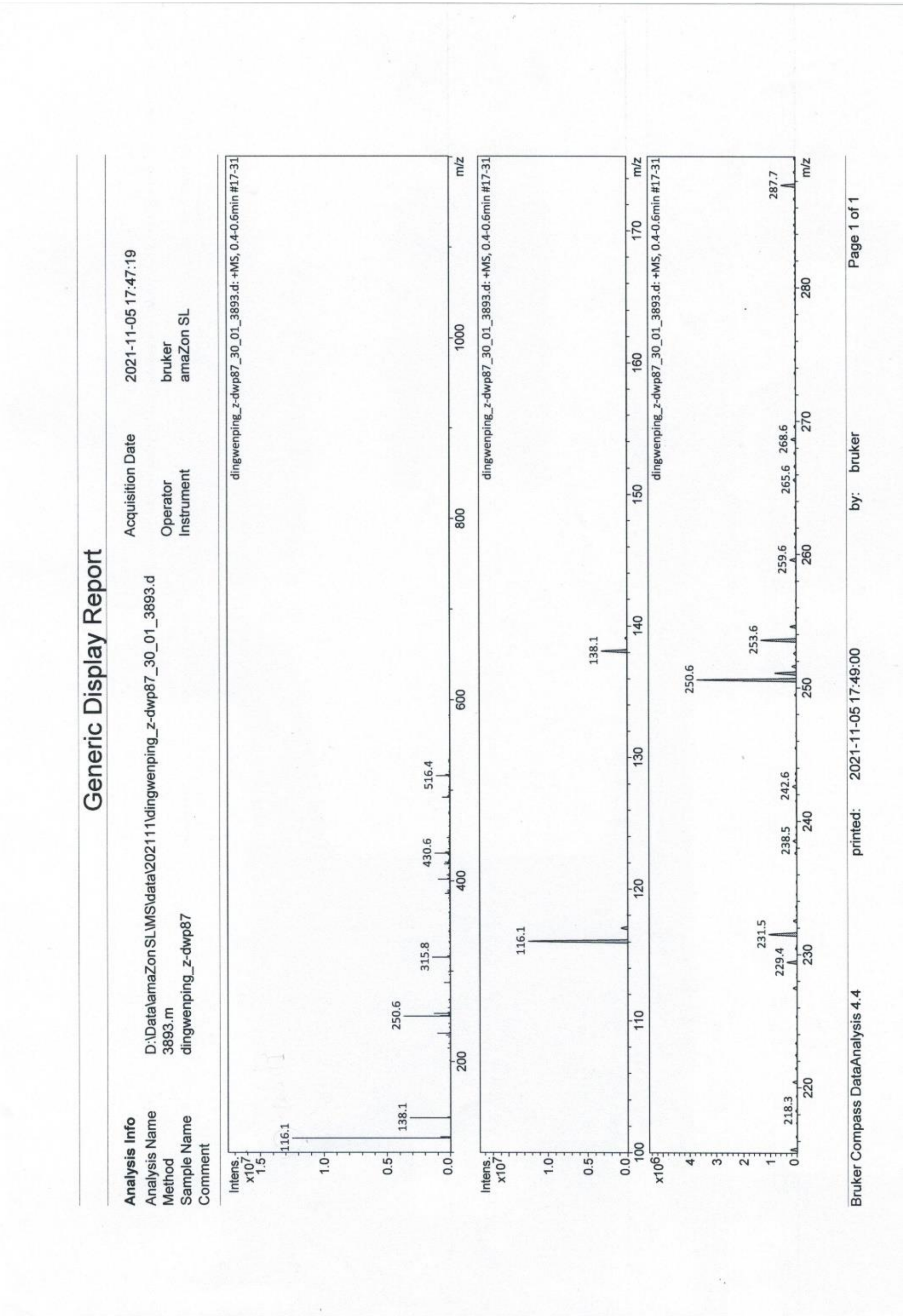

Fig. S109 ^{1}H NMR spectrum (CD_3OD, 500 MHz) of compound **23**

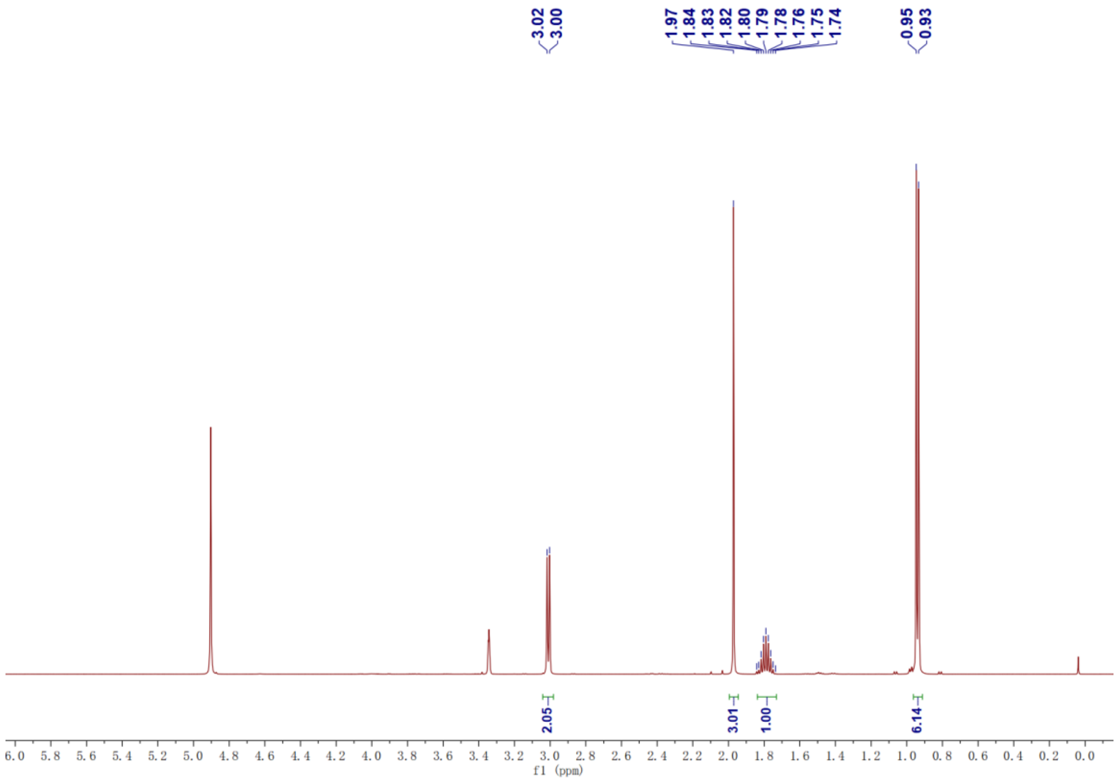

Fig. S110 ^{13}C NMR spectrum (CD_3OD, 126 MHz) of compound **23**

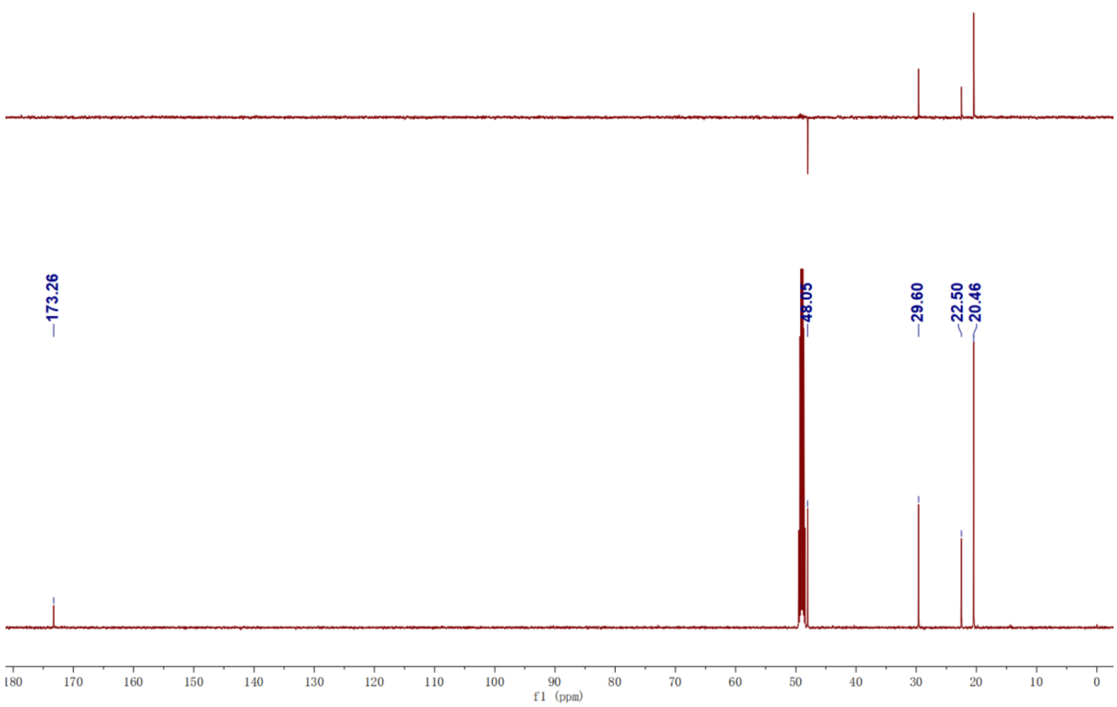

Fig. S111 Integrated cluster-node diagram of Molecular Networking

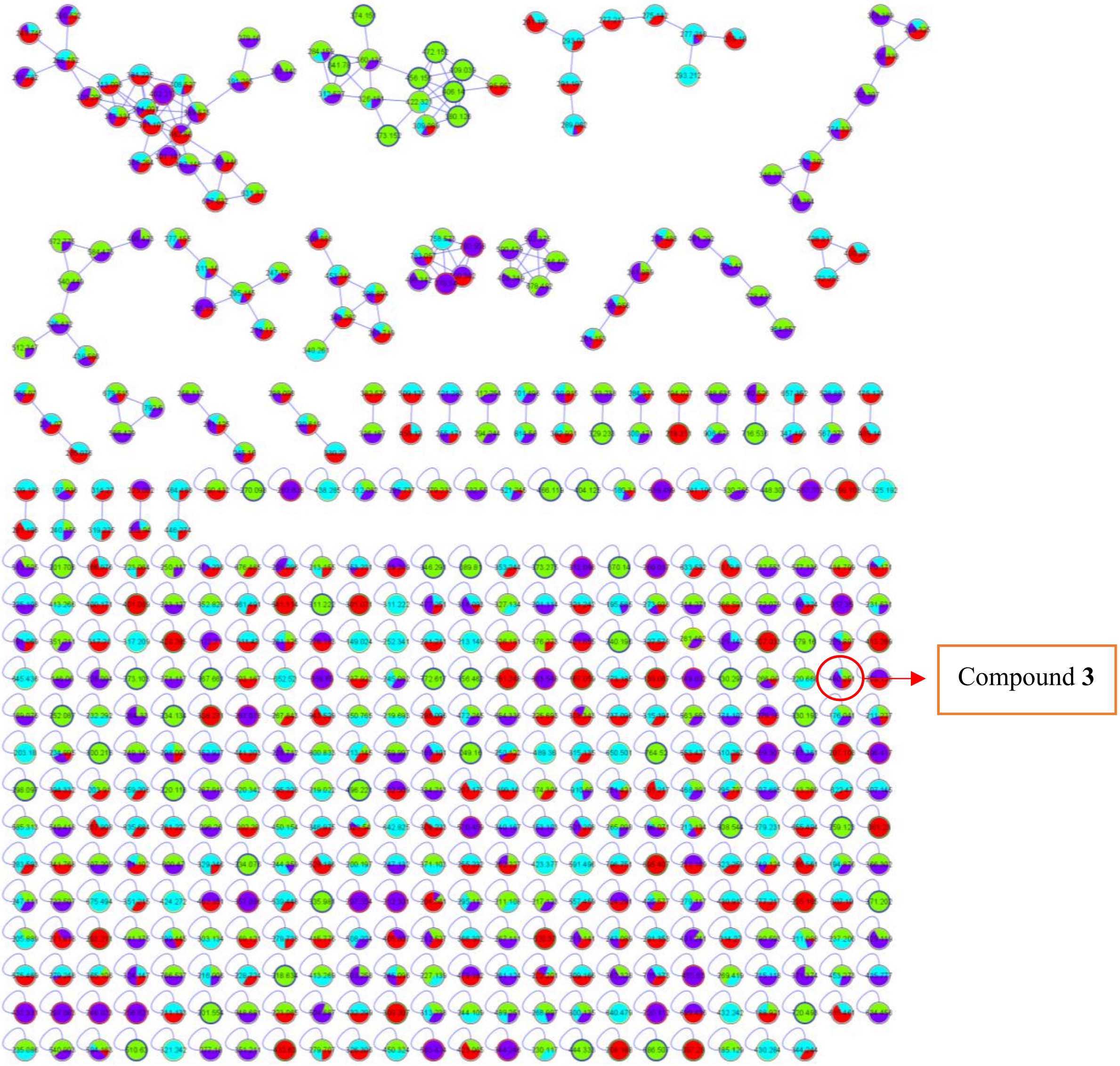

Supplement: Supplementary file 1 [file marinedrugs-20-00431-s001.zip › Supplemental data-revision.pdf]
